# Desorption properties of the R600, R134a and their mixtures in several MOF structures: A molecular dynamics study

**DOI:** 10.1016/j.heliyon.2023.e20774

**Published:** 2023-10-06

**Authors:** Wei Liu, Nan Wang, Jun Chen, Aijing Shen, Fu Liang

**Affiliations:** a State Power Investment Corporation Central Research Institute (SPICRI), Beijing, 100029, China; b Chongqing University, School of Graduate, Chongqing, 400030, China

**Keywords:** Metal organic framework, Refrigerants, Desorption, Molecular dynamics

## Abstract

The efficiency of organic Rankine cycle is expected to be enhanced via using metal organic heat carriers. Thus, it is important to investigate the properties of organic working fluid in metal organic frameworks (MOFs). In this paper, molecular dynamics method was employed to study the desorption properties of R600, R134a, and their mixtures in MOF-5, IRMOF-16, and MOF-200 structures. The results show that the desorption capacity of pure working fluids are negatively correlated with their molecular size. In mixed refrigerant systems, the desorption of the working medium is competitive, and the higher the proportion of fluorine atoms in the system, the greater the desorption heat in IRMOF-16. It is noteworthy that the behavior of the desorption heat in the other two MOFs is diametrically opposed to this result. The desorption capacity is positively correlated with the average pore size, specific surface area, and porosity of the MOF. The self-diffusion coefficient of the working medium in MOF is inversely proportional to its molecular weight, but directly proportional to its molecular size and to the pore size of MOF.

## 1 Introduction

The emergence of two global issues, the energy crisis and the greenhouse effect, is driving energy use in the direction of cleanliness and efficiency. There is a huge amount of waste heat resources that need to be recycled worldwide, such as turbine exhaust, internal combustion engine exhaust heat and steam cooling water, especially in developing countries with rapid economic growth such as India and China. Therefore, there is an urgent need to develop solutions for the efficient use of waste heat resources in order to improve energy efficiency and contribute to the advent of a zero-carbon era.

Organic Rankine Cycle (ORC) power generation technology with its simple system components is now widely used for the recovery of these low-grade energy sources [1,2]. The ORC system uses low boiling point organic refrigerants as the circulating medium. How to improve the efficiency of ORC has become a key issue in the utilization of low-grade energy [3,4]. McGrail et al. from the Pacific Northwest National Laboratory proposed to add a certain amount of nanoporous materials to the organic working medium to form a new type of metal-organic heat carrier (MOHC) nanofluid, which can achieve energy storage at the fluid-solid interface and thus improve the cycle performance [5]. In addition, the application of non-azeotropic mixed organic fluids to the organic Rankine cycle can also lead to an increase in system efficiency [6,7].

Compared with other nanoporous materials, MOFs are more promising as solid adsorbent materials for adsorption energy storage applications because of their large specific surface area and porosity and good thermal stability [8,9]. The search for MOFs with excellent energy storage performance is in a constant state of exploration [10]. Rezk et al. [11] experimentally investigated the adsorption performance of two MOFs, HKUST-1 (Cu-based) and MIL-100 (Fe-based), on water compared to silica gel RD-2060. The results showed that HKUST-1 had a 10.8% increase in storage energy density and nearly double the water adsorption capacity compared to silica RD-2060. Zheng et al. [12] successfully synthesized three pore size adjustable Ni-MOF-74 family members (Ni-MOF-74, Ni-MOF-74-BPP and Ni-MOF -74-TPP). They were found to perform well in the adsorption of water and R134a, with energy storage densities up to 62.6 kJ/mol and 50.6 kJ/mol, respectively. Henninger et al. [13,14] proposed a hydrophobic-hydrophilic water-stable 3D MOF (ISE-1), which was tested to have a water uptake of 210 g/kg at typical application temperatures and could be a candidate material for solid sorbents in refrigeration, heat pump and thermal storage conversion cycles. In addition, Elsayed et al. [15] conducted an experimental study on CPO-27(Ni), demonstrating its feasibility for application in energy storage.

Given the wide variety of MOFs available, experimental evaluation is an expensive, time-consuming, and tedious task. Molecular simulation [16] can be used as an efficient screening tool to identify potential adsorption energy storage working pairs and guide the experimental work of the most promising MOFs/refrigerant working pairs. So far, the reports on the adsorption energy storage of MOFs have mainly focused on the adsorption process and energy storage properties of organic workpiece [[17], [18], [19], [20], [21]], but less attention has been paid to the desorption behavior. Currently, the natural working fluid R600 (butane) is environmentally friendly with an Ozone Depletion Potential (ODP) of 0 and a Global Warming Potential (GWP) of only 4. Lu's work has shown that R600 has a high thermodynamic conversion capacity. The natural-like feedstock R134a (1,1,1,2-tetrafluoroethane) has been shown to have excellent cycling characteristics in ORC systems, with an ODP of 0 and a GWP of 1300. MOF-5 [22], as one of the simplest and earliest prepared iso-reticulated metal-organic frameworks (IRMOFs), has good thermal and chemical stability at no higher than 300 °C and is well suited to meet the requirements for low-grade energy sources in ORC. IRMOF-16, the largest pore size IRMOF, and MOF-200, another large pore size MOF, have been shown to be good candidates for Cold Thermal Energy Storage (CTES) devices [10]. The metal-organic frameworks MOF-5, IRMOF-16 and MOF-200 are therefore used to form the appropriate adsorption working pairs with the organic substances R600, R134a and their mixtures. Therefore, this paper studies the desorption processes of refrigerants in porous materials through molecular dynamics simulations.

## 2 Simulation details

### 2.1 Simulation model

**Fig. 1:**
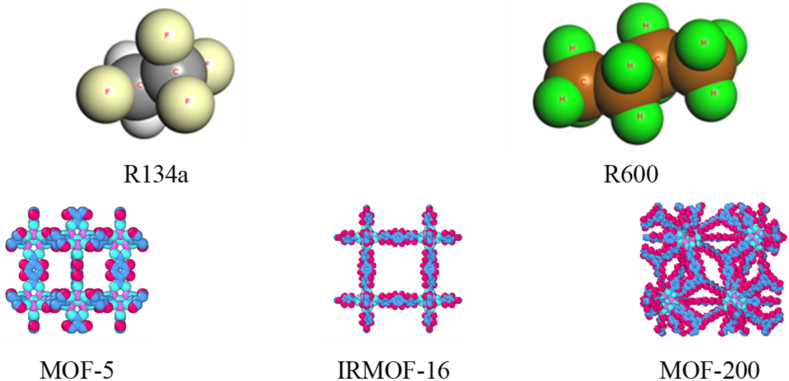
Selected organic working fluid and MOF structure diagram.

**Fig. 2:**
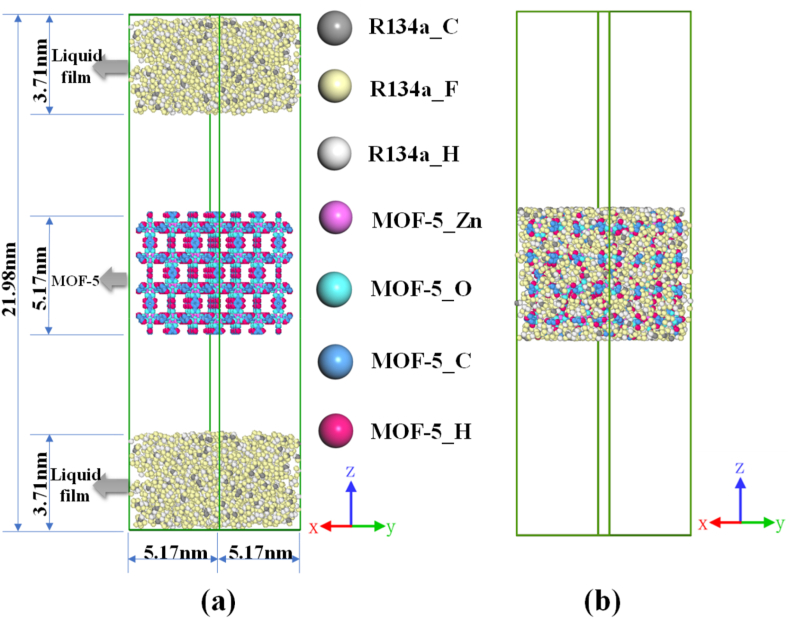
The initial configuration of adsorption and desorption of the R134a/MOF-5 system: (a) adsorption; (b) desorption.

In this paper, the selected organic working substances are the environmentally friendly natural working substance R600 and the natural-like working substance R134a [23,24], and three MOFs [10,25], namely MOF-5, IRMOF-16 and MOF-200 (as shown in Fig. 1). The desorption of pure R134a and R600 and their blends [R134a + R600 (1:1 M ratio), R134a + R600 (1:3 M ratio) and R134a + R600 (3:1 M ratio)] in MOF-5, IRMOF-16 and MOF-200 were simulated, respectively. The initial configuration of the adsorption system (shown in Fig. 2(a), using the R134a/MOF-5 system as an example) was obtained to complete the adsorption of the working fluid in the MOF before the desorption simulation. This model comprises a MOF-5 super-cell (2 × 2 × 2) and two R134a liquid films, which are 5.17 nm × 5.17 nm × 3.71 nm in the x, y and z directions. Two vacuum layers separate the MOF and liquid film for independent thermodynamic relaxation before the simulation. The MOF-5 super-cell was placed in the center of the system along the z direction and contains 3392 atoms (including 1536 C, 768 H, 832 O and 256 Zn). Two liquid films, both comprising 723 R^13^4a molecules, were arranged on each side of the system, and the mass fraction of MOF-5 in the whole system is 25%. A flexible force field was used in this study for a more accurate description of the interactions between the atoms in the MOF. In this study, the 12-6 Lennard-Jones (LJ) force field was used to describe the non-bonded interactions of the atoms of the MOFs, the specific LJ force field parameters are shown in Table 1 [26]. And the bonded interaction force parameters were taken from the work of Bureekaew et al. [27]. Their point charges were calculated using the charge equilibration (QEq) method [28]. In addition, the potential parameters for refrigerants were taken from previous works [[29], [30], [31]]. The Lorentz-Berthelot combination rule [32] was applied to calculate the parameters of non-bonded interactions between different kinds of atoms. The cut-off distance for intermolecular interactions is 1.2 nm. The long-range correction was applied to the LJ interactions. Long-distance Coulomb interactions were calculated by the particle-particle/particle grid (PPPM) method. Periodic boundary conditions were used in the x and y directions in the simulation box. The initial model for desorption regeneration was obtained from the model at the end of adsorption by deleting the un-adsorbed substances that were outside the MOF (as shown in Fig. 2(b)).

**Table 1:** The non-bonded force field parameters used for MOF-5, IRMOF-16 and MOF-200 in this study.

LJ parameters	MOF_Zn	MOF_C	MOF_O	MOF_H
σ (nm)	0.2462	0.3431	0.3118	0.2571
ε/kB (K)	62.40	52.84	30.19	22.14

### 2.2 Simulation parameters

The MD simulations were performed by the Large Scale Atomic/Molecular Parallel Simulator (LAMMPS) [33]. The adsorption process in the system was visualized by OVITO [34]. The whole simulation process can be divided into four steps: energy minimization, relaxation equilibrium, adsorption, and desorption. The energy minimization of the system was performed first after the initial model was constructed to get a stable molecular site pattern. Then, during the relaxation equilibrium, a constant temperature and volume system (NVT system) was utilized and the system temperature was controlled at 290 K. The relaxation equilibrium time step and simulation time is 1 fs (10^−15^ s) and 1 ns (10^−9^ s) respectively. The initial liquid phase densities of R134a and R600 pure and their blends at this temperature were referenced to the experimental data [35]. Therefore, the initial simulation box contains no gas phase, and the system is temperature and energy stable. The adsorption temperature was set to 300 K and the desorption temperatures were set to 320 K, 340 K, 360 K and 380 K. The simulations were performed at these five temperatures using the NVT system synthesis. The temperature was controlled by the Nose-Hoover method [36,37] and the Verlet algorithm was used to solve the atomic equations of motion. Both adsorption and desorption simulations were performed for 3 ns to ensure the completion of the adsorption and desorption process, with 2,000,000 steps (2ns) for equilibration and 1000,000 steps (1ns) for statistics. Macroscopic physical quantities (e.g. the amount of desorption and the heat of desorption) can be obtained by coefficient averaging of the physical quantities during the microscopic simulations. In addition, information on the atoms was output once every 10^4^ steps.

## 3 Results and discussion

**Fig. 3:**
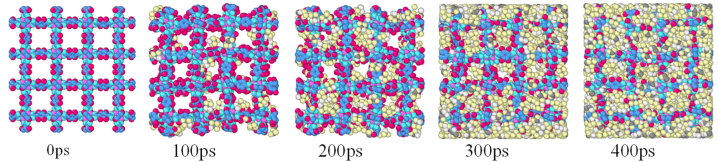
Snapshots of the adsorption simulation of R134a in MOF-5 at 300K.

Fig. 3 demonstrates snapshots of the adsorption simulation of R134a in MOF-5 at 300 K. It can be observed that the adsorption process was relatively rapid, reaching its peak at about 400 ps (10^−12^ s).

### 3.1 Desorption capacity

**Fig. 4:**
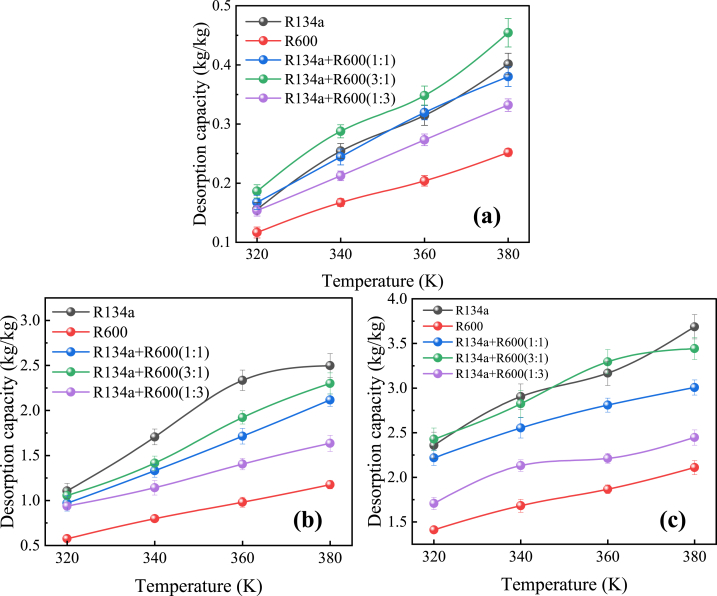
Desorption capacity of R134a, R600 and their mixed working fluids in each MOF at different temperatures: (a) MOF-5 (25 wt%); (b) IRMOF-16 (7.5 wt%); (c) MOF-200 (10 wt%).

At each temperature studied in the adsorption simulations, the desorption capacities of R134a, R600 pure and their blends in MOF-5, IRMOF-16 and MOF-200 are shown in Fig. 4. In this paper, the desorption capacity is defined as the mass of workpiece desorbed per unit mass of MOF, and its specific calculation formula is as follows:

(1) $$m_{desorption}=\frac{\left[M_{T0}-{\left(n_{R134a}m_{R134a}+n_{R600}m_{R600}\right)}_{T1}\right]}{M_{MOF}}$$

where *M*_*T*0_ and *M*_*MOF*_ are the absolute adsorption capacity of the workpiece at 300 K in the simulation model in this paper (in kg) and the absolute mass of the MOF portion shown (in kg), respectively. *n*_*R134a*_ and *n*_*R600*_ are the number of moles of R134a and R600 in the MOF at the end of the desorption simulation, respectively. In addition, *m*_*R134a*_ and *m*_*R600*_ are the molar masses of R134a and R600, respectively.

It can be found that the desorption capacities of the studied pure and mixed working substances in each MOF gradually increase with increasing temperature. This is due to the increase in thermodynamic energy of the fluids caused by the increase in temperature, which makes it easier to overcome the adsorption surface energy in contact with the MOF.

For the same adsorbent/MOF, the desorption capacity is proportional to the temperature. The molecular size of the pure adsorbent is negatively related to its desorption in MOFs. The molecular size of pure R600 is larger compared to pure R134a and the desorption capacity in each MOF is smaller at the same temperature. In addition, for the mixed medium system, there is a competition between the desorption behavior of the two substances, with the larger molecules inhibiting the desorption of the smaller molecules, and the inhibition becomes stronger with a higher proportion of the larger molecular.

**Fig. 5:**
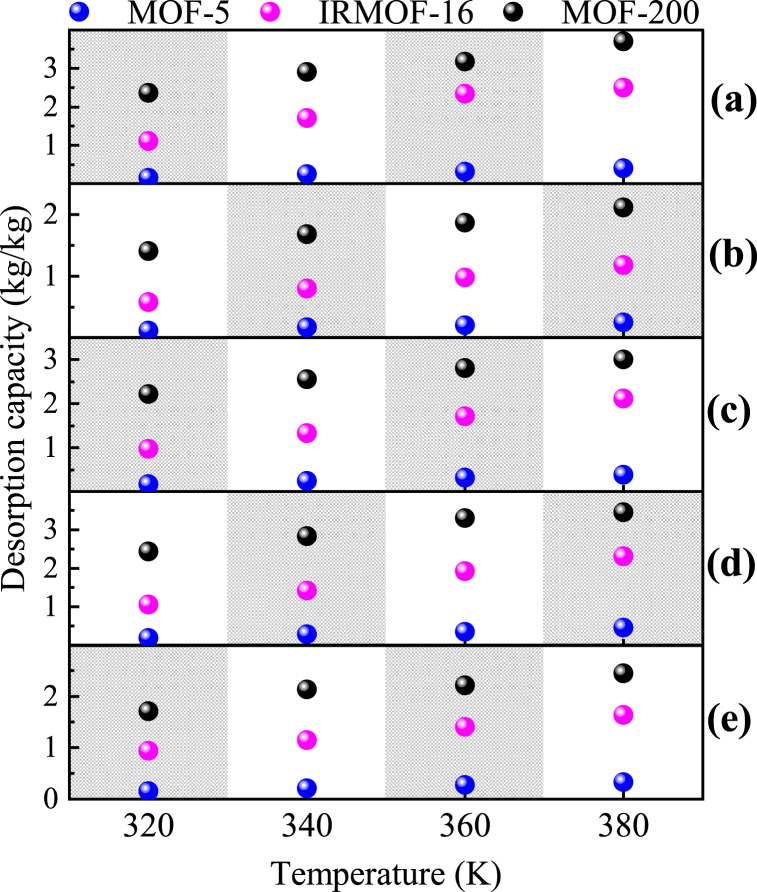
Comparison of desorption capacity of the same working fluid in MOF-5, IRMOF-16, and MOF-200 at different temperatures ((a) R134a, (b) R600, (c) R134a + R600(1:1), (d) R134a + R600(3:1), (e) R134a + R600(1:3)).

A comparison of the desorption capacities of the same working substances in MOF-5, IRMOF-16 and MOF-200 at different temperatures is displayed in Fig. 5. The adsorption amount in MOF-5 is the lowest. From the above observations and combined with the structural data of each MOF in Table 2, it can be analyzed that the desorption of the same fluid at the same temperature is positively correlated with the mean pore size, specific surface area and porosity of the MOF in a non-equal proportion, which is because as the mean pore size and porosity of the MOF increases, the adsorbate is more easily desorbed from the MOF.

**Table 2:** Comparison of tree MOFs selected in this study [10,[38], [39], [40]].

Items	MOF-5	IRMOF-16	MOF-200
Central metal ion	Zn^2+^	Zn^2+^	Zn^2+^
Molecular formula	C192H96O104Zn32	C480H288O104Zn32	C720H432O104Zn32
Molecular weight (g/mol)	6160.44	9823.65	12839.53
Specific surface area (m^2^/g)	3683.56	6045.79	6194.97
Hole volume (cm^3^/g)	1.3537	4.4801	4.2780
Average pore size (nm)	1.24	2.12	2.80
Porosity	0.8033	0.9194	0.9197

### 3.2 Desorption heat

The desorption heat is the thermal effect of the desorption process. In the desorption process, the thermodynamic energy of a gas or liquid molecule is significantly increased when it breaks away from the surface of the porous medium, completing the conversion between surface energy and thermodynamic energy. The desorption heat of per unit mass of MOF can be calculated using the following equation [41]：

(2) $$\Delta E=\frac{\left[E_{adsorbent+adsorbate}-\left(E_{adsorbent}+E_{adsorbate}\right)\right]}{M_{MOF}}$$

where *E*_*adsorbent + adsorbate*_ is the energy of the stable configuration formed prior to the desorption of organic workmates from the metal-organic frameworks, *E*_*adsorbent*_ denotes the energy after desorption of the metal-organic frameworks, and *E*_*adsorbate*_ denotes the energy after desorption of the organic working fluids. In addition, *M*_*MOF*_ is the absolute mass of the MOF portion shown.

**Fig. 6:**
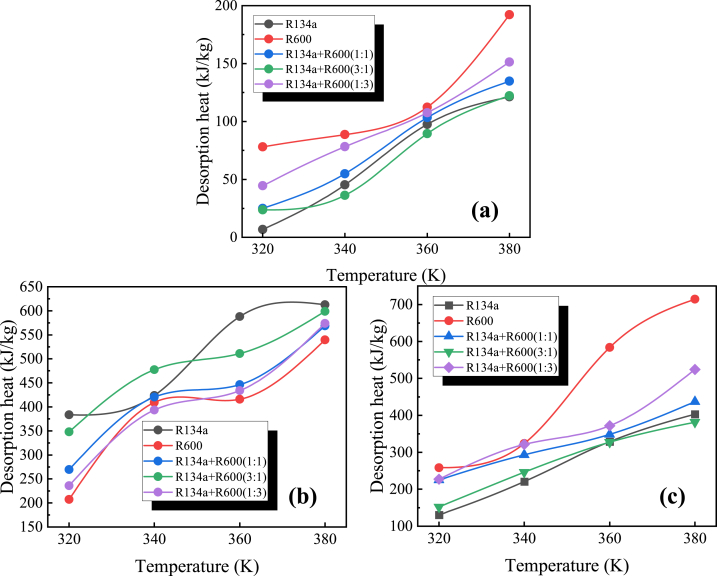
Desorption heat of R134a, R600 and their mixed working fluids in each MOF at different temperatures: (a) MOF-5 (25 wt%); (b) IRMOF-16 (7.5 wt%); (c) MOF-200 (10 wt%).

The desorption heat curves of R134a, R600 and their blends in MOF-5, IRMOF-16 and MOF-200 at different temperatures are shown in Fig. 6. It can be found that the heat of desorption in all three MOFs gradually increases with increasing simulation temperature for each system. Comparing the heat of desorption in MOF-5 and MOF-200 at the same temperature for different refrigerants, the relationship is most likely R134a < [R134a + R600 (3:1)]<[R134a + R600 (1:1)]<[ R134a + R600 (1:3)]<R600. It is noteworthy that the behavior of the desorption heat in IRMOF-16 is diametrically opposed to this result.

The above analysis leads to the conclusion that the molecular volume of the pure adsorbent is inversely related to the heat of desorption per unit mass of MOF in IRMOF-16. For mixed substances in this MOF, the higher the proportion of fluorine atoms, the higher the heat of desorption in the same MOF at the same temperature, which is probably because the fluorine atoms are more strongly interacting with other atoms during desorption. In the other two MOFs, weak fluorine interactions predominate.

**Fig. 7:**
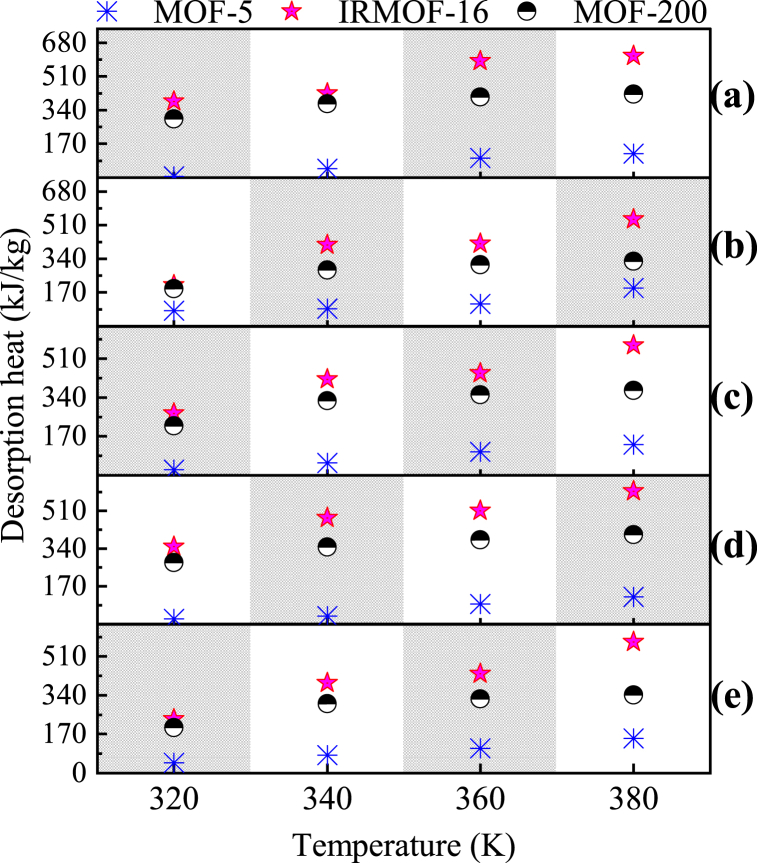
Comparison of desorption heat of the same working fluid in MOF-5, IRMOF-16 and MOF-200 at different temperatures ((a) R134a, (b) R600, (c) R134a + R600(1:1), (d) R134a + R600(3:1), (e) R134a + R600(1:3)).

The desorption heat of the same species in MOF-5, IRMOF-16 and MOF-200 at different temperatures is presented in Fig. 7. The desorption heat for the five adsorbents in this study is appeared to be IRMOF-16> MOF-200> MOF-5 at each temperature.

Based on the above findings, combined with the MOF structure data in Tables 2 and it can be determined that the desorption heat per unit mass of MOF is related to the topological structure of MOF at same temperatures. Both IRMOF-16 and MOF-200 have larger specific surface area, specific pore volume and pore size than MOF-5. Their heat of desorption at all temperatures is much higher than that of MOF-5. MOF-200 is an irregular three-dimensional curved surface, with the largest desorption heat of each substance at the same temperature. The pore of both MOF-5 and IRMOF-16 are regular rectangles. IRMOF-16 has a simpler pore shape than MOF-200, which gives it a superior heat of desorption.

### 3.3 Diffusion properties

The desorption of organic substances in MOFs is accompanied by diffusion behavior. The self-diffusion coefficient is a very important property of the diffusion properties, which reflects the degree of diffusion of the substances. The self-diffusion coefficient (D) can be calculated from the Mean Squared Displacement (MSD) [42]：

(3) $$MSD=\left\langle {\left|r\left(t\right)-r\left(0\right)\right|}^{2}\right\rangle$$

(4) $$D=\lim_{t\to \infty }\frac{1}{6t}MSD$$

where r(t) and r(0) represent the position vectors of the atoms at time t and the initial time respectively, represents the system average.

**Fig. 8:**
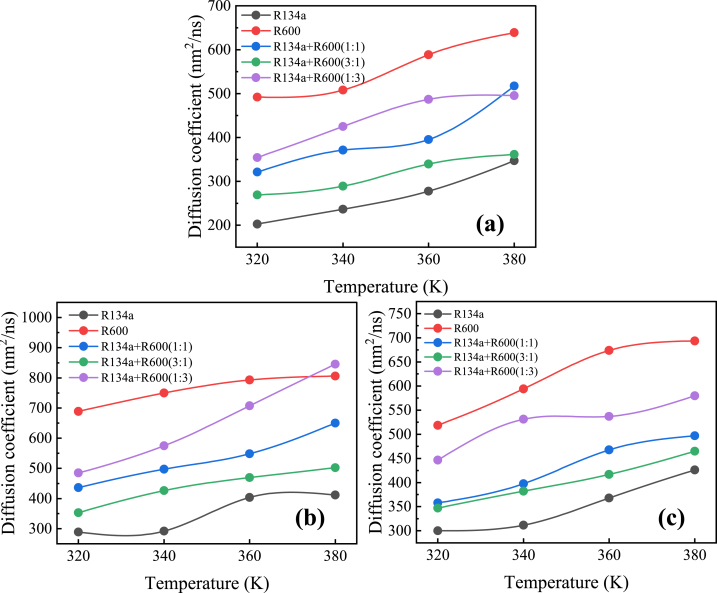
Diffusion coefficients of R134a, R600 and their mixed working fluids in each MOF at 320K: (a) MOF-5 (25 wt%); (b) IRMOF-16 (7.5 wt%); (c) MOF-200 (10 wt%).

The diffusion coefficients of R134a, R600, and their mixtures in each MOF at different temperatures are shown in Fig. 8. The raw MSD curves for the determination of these diffusion coefficients are shown in Figs. S1–S12 in the Supplemental files. As the temperature rises, the self-diffusion coefficients of all research systems increase, as an increase in temperature amplifies changes in the kinetic energy and position of molecules. Comparing the slope of the MSD curve over time for the same MOF for each system, the relationship between the magnitude of the self-diffusion coefficient is R600>[R134a + R600 (1:3)]>[R134a + R600 (1:1)]>[L134a + R6000 (3:1)]>R134a.

This indicates that at the same temperature, the self-diffusion coefficient of organic working compounds in MOF is inversely proportional to their molecular weight and positively correlated to their molecular size. R600 has a larger molecular size and smaller molecular weight than R134a, and its self-diffusion coefficient in each MOF is greater than R134a regardless of temperature. When macromolecules are added to a small molecule system, the self-diffusion coefficient increases, and the enhancement becomes more apparent as the proportion of macromolecules added increases.

**Fig. 9:**
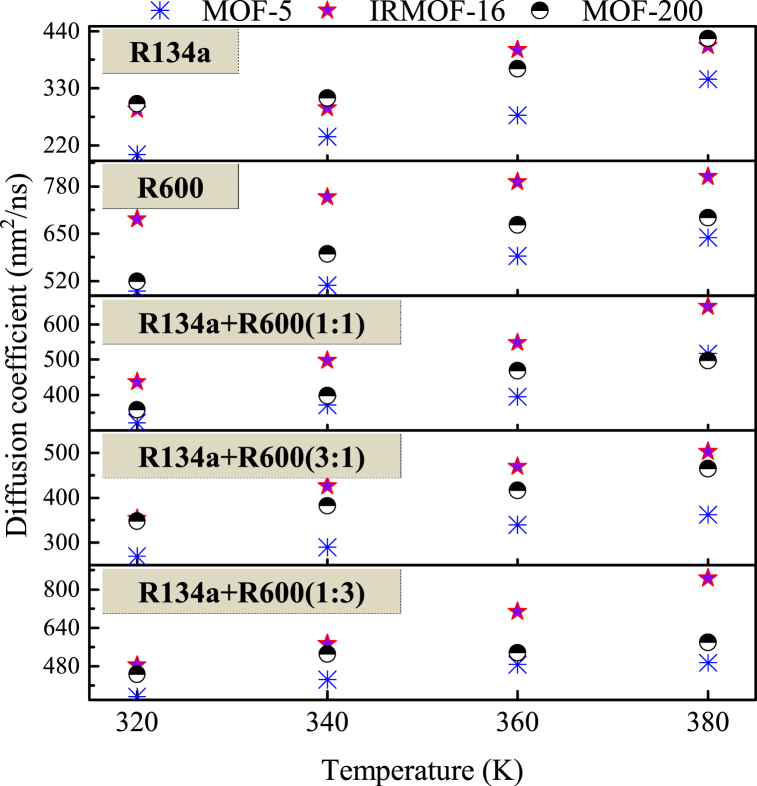
Comparison of diffusion coefficients of the same working fluid in MOF-5, IRMOF-16, and MOF-200 at different temperatures.

The comparison of diffusion coefficients curves for the same fluid at different temperatures (320 K, 340 K, 360 K, and 380 K) in MOF-5, IRMOF-16, and MOF-200 is shown in Fig. 9. It can be found that for almost all studied systems, the relationship between the magnitude of self-diffusion coefficients in the three MOFs at each temperature is IRMOF16> MOF-200> MOF-5.

The pore volume of IRMOF-16 is the largest among the three MOFs, while the pore volume of MOF-5 is the smallest. It can be inferred that for the same refrigerant, the self-diffusion coefficients of different MOFs at the same temperature are related to the pore volume of the MOF. The larger the pore volume, the greater the self-diffusion coefficient.

## 4 Conclusions

In this paper, the organic working substances R600, R134a and their blends were selected to investigate their desorption characteristics in MOF-5, IRMOF-16 and MOF-200, respectively, using the MD method. The main conclusions are as follows:

(1) For the same adsorbent/MOF, the desorption capacity is proportional to temperature. The molecular volume of pure adsorbent material is negatively correlated with its desorption in MOF. For a mixed mass adsorbent system, there is competition between the desorption behavior of the refrigerants, and the inhibition increases with the proportion of large volume molecular components.
(2) In IRMOF-16, The molecular volume of a pure adsorbent is inversely proportional to the desorption heat per unit mass of MOF. In a mixed system, the higher the proportion of fluorine atoms, the higher the desorption heat in IRMOF-16 at the same temperature. The desorption heat behavior of the systems in MOF-5 and MOF-200 are opposite to those of IRMOF-16.
(3) At the same temperature, the desorption capacity of R134a, R600, and their mixed working substances are positively correlated with the average pore size, specific surface area, and porosity of the MOF. The desorption heat per unit mass of MOF of the same substance is related to the specific pore volume and the shape and area of the MOF pore.
(4) At the same temperature, the self-diffusion coefficient of organic substances in MOF is inversely proportional to their molecular weight and directly proportional to their molecular size for the MOF/fluids combinations studied in this paper. Besides, the self-diffusion coefficients of R134a, R600, and their mixtures in different MOFs are related to the pore volume of the MOF. The larger the pore volume, the greater the self-diffusion coefficient.

## Author contributions

Conceived and designed the experiments, W.L. and N.W.; Performed the experiments, W.L. and J.C; Analyzed and interpreted the data, N.W., J.C. and A.S.; Contributed reagents, materials, analysis tools or data, W.L., J.C and F.L.; Wrote the paper, W.L.

## Funding

This research was funded by 10.13039/501100012477Fundamental Research Funds for the Key Research Program of Chongqing Science and Technology Commission (No. cstc2020jcyj-msxmX0840).

## Declaration of competing interest

The authors declare the following financial interests/personal relationships which may be considered as potential competing interests:Wei Liu reports financial support was provided by 10.13039/501100012477Fundamental Research Funds for the Key Research Program of Chongqing Science and Technology Commission (No. cstc2020jcyj-msxmX0840).
