# Supplementary material for: Desorption properties of the R600, R134a and their mixtures in several MOF structures: A molecular dynamics study
Source: Heliyon. 2023 Oct 6;9(10):e20774. doi: 10.1016/j.heliyon.2023.e20774 (PMC10568097; doi:10.1016/j.heliyon.2023.e20774)
Supplement: Multimedia component 1 [file mmc1.docx]

|   **Fig. S1.** MSD of the systems in MOF-5 at 320K. |   **Fig. S2.** MSD of the systems in MOF-5 at 340K. |
| --- | --- |
|   **Fig. S3.** MSD of the systems in MOF-5 at 360K. |   **Fig. S4.** MSD of the systems in MOF-5 at 380K. |
|   **Fig. S5.** MSD of the systems in IRMOF-16 at 320K. |   **Fig. S6.** MSD of the systems in IRMOF-16 at 340K. |
|   **Fig. S7.** MSD of the systems in IRMOF-16 at 360K. |   **Fig. S8.** MSD of the systems in IRMOF-16 at 380K. |
|   **Fig. S9.** MSD of the systems in MOF-200 at 320K. |   **Fig. S10.** MSD of the systems in MOF-200 at 340K. |
|   **Fig. S11.** MSD of the systems in MOF-200 at 360K. |   **Fig. S12.** MSD of the systems in MOF-200 at 380K. |

**1.#data file for MOF-5**

424 atoms

5 atom types

-0.000000000 25.832000000 xlo xhi

-0.000000000 25.832000000 ylo yhi

-0.000000000 25.832000000 zlo zhi

Atoms # full (The fourth column is charge)

1 1 1 0.4579 7.57911 5.33689 5.33689 0 0 0 # Zn3+2

2 1 1 0.4579 7.57911 18.25289 18.25289 0 0 0 # Zn3+2

3 1 1 0.4579 18.25289 20.49511 5.33689 0 0 0 # Zn3+2

4 1 1 0.4579 18.25289 7.57911 18.25289 0 0 0 # Zn3+2

5 1 1 0.4579 5.33689 7.57911 5.33689 0 0 0 # Zn3+2

6 1 1 0.4579 18.25289 5.33689 20.49511 0 0 0 # Zn3+2

7 1 1 0.4579 18.25289 18.25289 7.57911 0 0 0 # Zn3+2

8 1 1 0.4579 5.33689 5.33689 7.57911 0 0 0 # Zn3+2

9 1 1 0.4579 7.57911 20.49511 20.49511 0 0 0 # Zn3+2

10 1 1 0.4579 7.57911 7.57911 7.57911 0 0 0 # Zn3+2

11 1 1 0.4579 5.33689 18.25289 20.49511 0 0 0 # Zn3+2

12 1 1 0.4579 20.49511 18.25289 5.33689 0 0 0 # Zn3+2

13 1 1 0.4579 20.49511 7.57911 20.49511 0 0 0 # Zn3+2

14 1 1 0.4579 20.49511 5.33689 18.25289 0 0 0 # Zn3+2

15 1 1 0.4579 5.33689 20.49511 18.25289 0 0 0 # Zn3+2

16 1 1 0.4579 20.49511 20.49511 7.57911 0 0 0 # Zn3+2

17 1 1 0.4579 5.33689 7.57911 20.49511 0 0 0 # Zn3+2

18 1 1 0.4579 20.49511 18.25289 20.49511 0 0 0 # Zn3+2

19 1 1 0.4579 20.49511 5.33689 7.57911 0 0 0 # Zn3+2

20 1 1 0.4579 7.57911 5.33689 20.49511 0 0 0 # Zn3+2

21 1 1 0.4579 5.33689 18.25289 5.33689 0 0 0 # Zn3+2

22 1 1 0.4579 20.49511 7.57911 5.33689 0 0 0 # Zn3+2

23 1 1 0.4579 7.57911 7.57911 18.25289 0 0 0 # Zn3+2

24 1 1 0.4579 7.57911 18.25289 7.57911 0 0 0 # Zn3+2

25 1 1 0.4579 18.25289 5.33689 5.33689 0 0 0 # Zn3+2

26 1 1 0.4579 18.25289 18.25289 18.25289 0 0 0 # Zn3+2

27 1 1 0.4579 18.25289 20.49511 20.49511 0 0 0 # Zn3+2

28 1 1 0.4579 18.25289 7.57911 7.57911 0 0 0 # Zn3+2

29 1 1 0.4579 5.33689 20.49511 7.57911 0 0 0 # Zn3+2

30 1 1 0.4579 7.57911 20.49511 5.33689 0 0 0 # Zn3+2

31 1 1 0.4579 5.33689 5.33689 18.25289 0 0 0 # Zn3+2

32 1 1 0.4579 20.49511 20.49511 18.25289 0 0 0 # Zn3+2

33 1 2 -0.2317 6.458 6.458 6.458 0 0 0 # O_3

34 1 2 -0.2317 19.374 19.374 6.458 0 0 0 # O_3

35 1 2 -0.2317 19.374 6.458 19.374 0 0 0 # O_3

36 1 2 -0.2317 6.458 19.374 19.374 0 0 0 # O_3

37 1 2 -0.2317 6.458 6.458 19.374 0 0 0 # O_3

38 1 2 -0.2317 19.374 19.374 19.374 0 0 0 # O_3

39 1 2 -0.2317 19.374 6.458 6.458 0 0 0 # O_3

40 1 2 -0.2317 6.458 19.374 6.458 0 0 0 # O_3

41 1 3 -0.4677 7.28204 5.63396 3.46149 0 0 0 # O_2

42 1 4 -0.1413 7.30787 5.60813 0.69488 0 0 0 # C_R

43 1 5 0.1652 7.87618 5.03982 1.15727 0 0 0 # H_

44 1 3 -0.4677 7.28204 18.54996 16.37749 0 0 0 # O_2

45 1 4 -0.1413 7.30787 18.52413 13.61088 0 0 0 # C_R

46 1 5 0.1652 7.87618 17.95582 14.07327 0 0 0 # H_

47 1 3 -0.4677 18.54996 20.19804 3.46149 0 0 0 # O_2

48 1 4 -0.1413 18.52413 20.22387 0.69488 0 0 0 # C_R

49 1 5 0.1652 17.95582 20.79218 1.15727 0 0 0 # H_

50 1 3 -0.4677 18.54996 7.28204 16.37749 0 0 0 # O_2

51 1 4 -0.1413 18.52413 7.30787 13.61088 0 0 0 # C_R

52 1 5 0.1652 17.95582 7.87618 14.07327 0 0 0 # H_

53 1 3 -0.4677 5.63396 7.28204 3.46149 0 0 0 # O_2

54 1 4 -0.1413 5.60813 7.30787 0.69488 0 0 0 # C_R

55 1 5 0.1652 5.03982 7.87618 1.15727 0 0 0 # H_

56 1 3 -0.4677 18.54996 5.63396 22.37051 0 0 0 # O_2

57 1 4 -0.1413 18.52413 5.60813 25.13712 0 0 0 # C_R

58 1 5 0.1652 17.95582 5.03982 24.67473 0 0 0 # H_

59 1 3 -0.4677 18.54996 18.54996 9.45451 0 0 0 # O_2

60 1 4 -0.1413 18.52413 18.52413 12.22112 0 0 0 # C_R

61 1 5 0.1652 17.95582 17.95582 11.75873 0 0 0 # H_

62 1 3 -0.4677 5.63396 5.63396 9.45451 0 0 0 # O_2

63 1 4 -0.1413 5.60813 5.60813 12.22112 0 0 0 # C_R

64 1 5 0.1652 5.03982 5.03982 11.75873 0 0 0 # H_

65 1 3 -0.4677 7.28204 20.19804 22.37051 0 0 0 # O_2

66 1 4 -0.1413 7.30787 20.22387 25.13712 0 0 0 # C_R

67 1 5 0.1652 7.87618 20.79218 24.67473 0 0 0 # H_

68 1 3 -0.4677 7.28204 7.28204 9.45451 0 0 0 # O_2

69 1 4 -0.1413 7.30787 7.30787 12.22112 0 0 0 # C_R

70 1 5 0.1652 7.87618 7.87618 11.75873 0 0 0 # H_

71 1 3 -0.4677 3.46149 7.28204 5.63396 0 0 0 # O_2

72 1 4 -0.1413 0.69488 7.30787 5.60813 0 0 0 # C_R

73 1 5 0.1652 1.15727 7.87618 5.03982 0 0 0 # H_

74 1 3 -0.4677 16.37749 7.28204 18.54996 0 0 0 # O_2

75 1 4 -0.1413 13.61088 7.30787 18.52413 0 0 0 # C_R

76 1 5 0.1652 14.07327 7.87618 17.95582 0 0 0 # H_

77 1 3 -0.4677 3.46149 18.54996 20.19804 0 0 0 # O_2

78 1 4 -0.1413 0.69488 18.52413 20.22387 0 0 0 # C_R

79 1 5 0.1652 1.15727 17.95582 20.79218 0 0 0 # H_

80 1 3 -0.4677 3.46149 5.63396 7.28204 0 0 0 # O_2

81 1 4 -0.1413 0.69488 5.60813 7.30787 0 0 0 # C_R

82 1 5 0.1652 1.15727 5.03982 7.87618 0 0 0 # H_

83 1 3 -0.4677 16.37749 18.54996 7.28204 0 0 0 # O_2

84 1 4 -0.1413 13.61088 18.52413 7.30787 0 0 0 # C_R

85 1 5 0.1652 14.07327 17.95582 7.87618 0 0 0 # H_

86 1 3 -0.4677 22.37051 18.54996 5.63396 0 0 0 # O_2

87 1 4 -0.1413 25.13712 18.52413 5.60813 0 0 0 # C_R

88 1 5 0.1652 24.67473 17.95582 5.03982 0 0 0 # H_

89 1 3 -0.4677 9.45451 18.54996 18.54996 0 0 0 # O_2

90 1 4 -0.1413 12.22112 18.52413 18.52413 0 0 0 # C_R

91 1 5 0.1652 11.75873 17.95582 17.95582 0 0 0 # H_

92 1 3 -0.4677 9.45451 5.63396 5.63396 0 0 0 # O_2

93 1 4 -0.1413 12.22112 5.60813 5.60813 0 0 0 # C_R

94 1 5 0.1652 11.75873 5.03982 5.03982 0 0 0 # H_

95 1 3 -0.4677 22.37051 7.28204 20.19804 0 0 0 # O_2

96 1 4 -0.1413 25.13712 7.30787 20.22387 0 0 0 # C_R

97 1 5 0.1652 24.67473 7.87618 20.79218 0 0 0 # H_

98 1 3 -0.4677 9.45451 7.28204 7.28204 0 0 0 # O_2

99 1 4 -0.1413 12.22112 7.30787 7.30787 0 0 0 # C_R

100 1 5 0.1652 11.75873 7.87618 7.87618 0 0 0 # H_

101 1 3 -0.4677 5.63396 3.46149 7.28204 0 0 0 # O_2

102 1 4 -0.1413 5.60813 0.69488 7.30787 0 0 0 # C_R

103 1 5 0.1652 5.03982 1.15727 7.87618 0 0 0 # H_

104 1 3 -0.4677 5.63396 16.37749 20.19804 0 0 0 # O_2

105 1 4 -0.1413 5.60813 13.61088 20.22387 0 0 0 # C_R

106 1 5 0.1652 5.03982 14.07327 20.79218 0 0 0 # H_

107 1 3 -0.4677 20.19804 3.46149 18.54996 0 0 0 # O_2

108 1 4 -0.1413 20.22387 0.69488 18.52413 0 0 0 # C_R

109 1 5 0.1652 20.79218 1.15727 17.95582 0 0 0 # H_

110 1 3 -0.4677 20.19804 16.37749 5.63396 0 0 0 # O_2

111 1 4 -0.1413 20.22387 13.61088 5.60813 0 0 0 # C_R

112 1 5 0.1652 20.79218 14.07327 5.03982 0 0 0 # H_

113 1 3 -0.4677 7.28204 3.46149 5.63396 0 0 0 # O_2

114 1 4 -0.1413 7.30787 0.69488 5.60813 0 0 0 # C_R

115 1 5 0.1652 7.87618 1.15727 5.03982 0 0 0 # H_

116 1 3 -0.4677 5.63396 22.37051 18.54996 0 0 0 # O_2

117 1 4 -0.1413 5.60813 25.13712 18.52413 0 0 0 # C_R

118 1 5 0.1652 5.03982 24.67473 17.95582 0 0 0 # H_

119 1 3 -0.4677 5.63396 9.45451 5.63396 0 0 0 # O_2

120 1 4 -0.1413 5.60813 12.22112 5.60813 0 0 0 # C_R

121 1 5 0.1652 5.03982 11.75873 5.03982 0 0 0 # H_

122 1 3 -0.4677 20.19804 22.37051 7.28204 0 0 0 # O_2

123 1 4 -0.1413 20.22387 25.13712 7.30787 0 0 0 # C_R

124 1 5 0.1652 20.79218 24.67473 7.87618 0 0 0 # H_

125 1 3 -0.4677 20.19804 9.45451 20.19804 0 0 0 # O_2

126 1 4 -0.1413 20.22387 12.22112 20.22387 0 0 0 # C_R

127 1 5 0.1652 20.79218 11.75873 20.79218 0 0 0 # H_

128 1 3 -0.4677 7.28204 9.45451 7.28204 0 0 0 # O_2

129 1 4 -0.1413 7.30787 12.22112 7.30787 0 0 0 # C_R

130 1 5 0.1652 7.87618 11.75873 7.87618 0 0 0 # H_

131 1 3 -0.4677 5.63396 7.28204 22.37051 0 0 0 # O_2

132 1 4 -0.1413 5.60813 7.30787 25.13712 0 0 0 # C_R

133 1 5 0.1652 5.03982 7.87618 24.67473 0 0 0 # H_

134 1 3 -0.4677 5.63396 20.19804 9.45451 0 0 0 # O_2

135 1 4 -0.1413 5.60813 20.22387 12.22112 0 0 0 # C_R

136 1 5 0.1652 5.03982 20.79218 11.75873 0 0 0 # H_

137 1 3 -0.4677 20.19804 18.54996 22.37051 0 0 0 # O_2

138 1 4 -0.1413 20.22387 18.52413 25.13712 0 0 0 # C_R

139 1 5 0.1652 20.79218 17.95582 24.67473 0 0 0 # H_

140 1 3 -0.4677 20.19804 5.63396 9.45451 0 0 0 # O_2

141 1 4 -0.1413 20.22387 5.60813 12.22112 0 0 0 # C_R

142 1 5 0.1652 20.79218 5.03982 11.75873 0 0 0 # H_

143 1 3 -0.4677 7.28204 5.63396 22.37051 0 0 0 # O_2

144 1 4 -0.1413 7.30787 5.60813 25.13712 0 0 0 # C_R

145 1 5 0.1652 7.87618 5.03982 24.67473 0 0 0 # H_

146 1 3 -0.4677 5.63396 18.54996 3.46149 0 0 0 # O_2

147 1 4 -0.1413 5.60813 18.52413 0.69488 0 0 0 # C_R

148 1 5 0.1652 5.03982 17.95582 1.15727 0 0 0 # H_

149 1 3 -0.4677 5.63396 5.63396 16.37749 0 0 0 # O_2

150 1 4 -0.1413 5.60813 5.60813 13.61088 0 0 0 # C_R

151 1 5 0.1652 5.03982 5.03982 14.07327 0 0 0 # H_

152 1 3 -0.4677 20.19804 7.28204 3.46149 0 0 0 # O_2

153 1 4 -0.1413 20.22387 7.30787 0.69488 0 0 0 # C_R

154 1 5 0.1652 20.79218 7.87618 1.15727 0 0 0 # H_

155 1 3 -0.4677 20.19804 20.19804 16.37749 0 0 0 # O_2

156 1 4 -0.1413 20.22387 20.22387 13.61088 0 0 0 # C_R

157 1 5 0.1652 20.79218 20.79218 14.07327 0 0 0 # H_

158 1 3 -0.4677 7.28204 7.28204 16.37749 0 0 0 # O_2

159 1 4 -0.1413 7.30787 7.30787 13.61088 0 0 0 # C_R

160 1 5 0.1652 7.87618 7.87618 14.07327 0 0 0 # H_

161 1 3 -0.4677 7.28204 3.46149 20.19804 0 0 0 # O_2

162 1 4 -0.1413 7.30787 0.69488 20.22387 0 0 0 # C_R

163 1 5 0.1652 7.87618 1.15727 20.79218 0 0 0 # H_

164 1 3 -0.4677 7.28204 16.37749 7.28204 0 0 0 # O_2

165 1 4 -0.1413 7.30787 13.61088 7.30787 0 0 0 # C_R

166 1 5 0.1652 7.87618 14.07327 7.87618 0 0 0 # H_

167 1 3 -0.4677 18.54996 3.46149 5.63396 0 0 0 # O_2

168 1 4 -0.1413 18.52413 0.69488 5.60813 0 0 0 # C_R

169 1 5 0.1652 17.95582 1.15727 5.03982 0 0 0 # H_

170 1 3 -0.4677 18.54996 16.37749 18.54996 0 0 0 # O_2

171 1 4 -0.1413 18.52413 13.61088 18.52413 0 0 0 # C_R

172 1 5 0.1652 17.95582 14.07327 17.95582 0 0 0 # H_

173 1 3 -0.4677 5.63396 16.37749 5.63396 0 0 0 # O_2

174 1 4 -0.1413 5.60813 13.61088 5.60813 0 0 0 # C_R

175 1 5 0.1652 5.03982 14.07327 5.03982 0 0 0 # H_

176 1 3 -0.4677 18.54996 22.37051 20.19804 0 0 0 # O_2

177 1 4 -0.1413 18.52413 25.13712 20.22387 0 0 0 # C_R

178 1 5 0.1652 17.95582 24.67473 20.79218 0 0 0 # H_

179 1 3 -0.4677 18.54996 9.45451 7.28204 0 0 0 # O_2

180 1 4 -0.1413 18.52413 12.22112 7.30787 0 0 0 # C_R

181 1 5 0.1652 17.95582 11.75873 7.87618 0 0 0 # H_

182 1 3 -0.4677 5.63396 22.37051 7.28204 0 0 0 # O_2

183 1 4 -0.1413 5.60813 25.13712 7.30787 0 0 0 # C_R

184 1 5 0.1652 5.03982 24.67473 7.87618 0 0 0 # H_

185 1 3 -0.4677 7.28204 22.37051 5.63396 0 0 0 # O_2

186 1 4 -0.1413 7.30787 25.13712 5.60813 0 0 0 # C_R

187 1 5 0.1652 7.87618 24.67473 5.03982 0 0 0 # H_

188 1 3 -0.4677 7.28204 9.45451 18.54996 0 0 0 # O_2

189 1 4 -0.1413 7.30787 12.22112 18.52413 0 0 0 # C_R

190 1 5 0.1652 7.87618 11.75873 17.95582 0 0 0 # H_

191 1 3 -0.4677 3.46149 5.63396 18.54996 0 0 0 # O_2

192 1 4 -0.1413 0.69488 5.60813 18.52413 0 0 0 # C_R

193 1 5 0.1652 1.15727 5.03982 17.95582 0 0 0 # H_

194 1 3 -0.4677 16.37749 5.63396 5.63396 0 0 0 # O_2

195 1 4 -0.1413 13.61088 5.60813 5.60813 0 0 0 # C_R

196 1 5 0.1652 14.07327 5.03982 5.03982 0 0 0 # H_

197 1 3 -0.4677 3.46149 20.19804 7.28204 0 0 0 # O_2

198 1 4 -0.1413 0.69488 20.22387 7.30787 0 0 0 # C_R

199 1 5 0.1652 1.15727 20.79218 7.87618 0 0 0 # H_

200 1 3 -0.4677 16.37749 20.19804 20.19804 0 0 0 # O_2

201 1 4 -0.1413 13.61088 20.22387 20.22387 0 0 0 # C_R

202 1 5 0.1652 14.07327 20.79218 20.79218 0 0 0 # H_

203 1 3 -0.4677 16.37749 7.28204 7.28204 0 0 0 # O_2

204 1 4 -0.1413 13.61088 7.30787 7.30787 0 0 0 # C_R

205 1 5 0.1652 14.07327 7.87618 7.87618 0 0 0 # H_

206 1 3 -0.4677 22.37051 5.63396 7.28204 0 0 0 # O_2

207 1 4 -0.1413 25.13712 5.60813 7.30787 0 0 0 # C_R

208 1 5 0.1652 24.67473 5.03982 7.87618 0 0 0 # H_

209 1 3 -0.4677 9.45451 5.63396 20.19804 0 0 0 # O_2

210 1 4 -0.1413 12.22112 5.60813 20.22387 0 0 0 # C_R

211 1 5 0.1652 11.75873 5.03982 20.79218 0 0 0 # H_

212 1 3 -0.4677 22.37051 20.19804 18.54996 0 0 0 # O_2

213 1 4 -0.1413 25.13712 20.22387 18.52413 0 0 0 # C_R

214 1 5 0.1652 24.67473 20.79218 17.95582 0 0 0 # H_

215 1 3 -0.4677 22.37051 7.28204 5.63396 0 0 0 # O_2

216 1 4 -0.1413 25.13712 7.30787 5.60813 0 0 0 # C_R

217 1 5 0.1652 24.67473 7.87618 5.03982 0 0 0 # H_

218 1 3 -0.4677 9.45451 20.19804 5.63396 0 0 0 # O_2

219 1 4 -0.1413 12.22112 20.22387 5.60813 0 0 0 # C_R

220 1 5 0.1652 11.75873 20.79218 5.03982 0 0 0 # H_

221 1 3 -0.4677 18.54996 20.19804 22.37051 0 0 0 # O_2

222 1 4 -0.1413 18.52413 20.22387 25.13712 0 0 0 # C_R

223 1 5 0.1652 17.95582 20.79218 24.67473 0 0 0 # H_

224 1 3 -0.4677 18.54996 7.28204 9.45451 0 0 0 # O_2

225 1 4 -0.1413 18.52413 7.30787 12.22112 0 0 0 # C_R

226 1 5 0.1652 17.95582 7.87618 11.75873 0 0 0 # H_

227 1 3 -0.4677 7.28204 18.54996 9.45451 0 0 0 # O_2

228 1 4 -0.1413 7.30787 18.52413 12.22112 0 0 0 # C_R

229 1 5 0.1652 7.87618 17.95582 11.75873 0 0 0 # H_

230 1 3 -0.4677 7.28204 20.19804 3.46149 0 0 0 # O_2

231 1 4 -0.1413 7.30787 20.22387 0.69488 0 0 0 # C_R

232 1 5 0.1652 7.87618 20.79218 1.15727 0 0 0 # H_

233 1 3 -0.4677 18.54996 5.63396 3.46149 0 0 0 # O_2

234 1 4 -0.1413 18.52413 5.60813 0.69488 0 0 0 # C_R

235 1 5 0.1652 17.95582 5.03982 1.15727 0 0 0 # H_

236 1 3 -0.4677 18.54996 18.54996 16.37749 0 0 0 # O_2

237 1 4 -0.1413 18.52413 18.52413 13.61088 0 0 0 # C_R

238 1 5 0.1652 17.95582 17.95582 14.07327 0 0 0 # H_

239 1 3 -0.4677 22.37051 18.54996 20.19804 0 0 0 # O_2

240 1 4 -0.1413 25.13712 18.52413 20.22387 0 0 0 # C_R

241 1 5 0.1652 24.67473 17.95582 20.79218 0 0 0 # H_

242 1 3 -0.4677 9.45451 18.54996 7.28204 0 0 0 # O_2

243 1 4 -0.1413 12.22112 18.52413 7.30787 0 0 0 # C_R

244 1 5 0.1652 11.75873 17.95582 7.87618 0 0 0 # H_

245 1 3 -0.4677 9.45451 7.28204 18.54996 0 0 0 # O_2

246 1 4 -0.1413 12.22112 7.30787 18.52413 0 0 0 # C_R

247 1 5 0.1652 11.75873 7.87618 17.95582 0 0 0 # H_

248 1 3 -0.4677 3.46149 7.28204 20.19804 0 0 0 # O_2

249 1 4 -0.1413 0.69488 7.30787 20.22387 0 0 0 # C_R

250 1 5 0.1652 1.15727 7.87618 20.79218 0 0 0 # H_

251 1 3 -0.4677 3.46149 18.54996 5.63396 0 0 0 # O_2

252 1 4 -0.1413 0.69488 18.52413 5.60813 0 0 0 # C_R

253 1 5 0.1652 1.15727 17.95582 5.03982 0 0 0 # H_

254 1 3 -0.4677 16.37749 18.54996 18.54996 0 0 0 # O_2

255 1 4 -0.1413 13.61088 18.52413 18.52413 0 0 0 # C_R

256 1 5 0.1652 14.07327 17.95582 17.95582 0 0 0 # H_

257 1 3 -0.4677 20.19804 22.37051 18.54996 0 0 0 # O_2

258 1 4 -0.1413 20.22387 25.13712 18.52413 0 0 0 # C_R

259 1 5 0.1652 20.79218 24.67473 17.95582 0 0 0 # H_

260 1 3 -0.4677 20.19804 9.45451 5.63396 0 0 0 # O_2

261 1 4 -0.1413 20.22387 12.22112 5.60813 0 0 0 # C_R

262 1 5 0.1652 20.79218 11.75873 5.03982 0 0 0 # H_

263 1 3 -0.4677 5.63396 9.45451 20.19804 0 0 0 # O_2

264 1 4 -0.1413 5.60813 12.22112 20.22387 0 0 0 # C_R

265 1 5 0.1652 5.03982 11.75873 20.79218 0 0 0 # H_

266 1 3 -0.4677 20.19804 3.46149 7.28204 0 0 0 # O_2

267 1 4 -0.1413 20.22387 0.69488 7.30787 0 0 0 # C_R

268 1 5 0.1652 20.79218 1.15727 7.87618 0 0 0 # H_

269 1 3 -0.4677 20.19804 16.37749 20.19804 0 0 0 # O_2

270 1 4 -0.1413 20.22387 13.61088 20.22387 0 0 0 # C_R

271 1 5 0.1652 20.79218 14.07327 20.79218 0 0 0 # H_

272 1 3 -0.4677 5.63396 3.46149 18.54996 0 0 0 # O_2

273 1 4 -0.1413 5.60813 0.69488 18.52413 0 0 0 # C_R

274 1 5 0.1652 5.03982 1.15727 17.95582 0 0 0 # H_

275 1 3 -0.4677 20.19804 18.54996 3.46149 0 0 0 # O_2

276 1 4 -0.1413 20.22387 18.52413 0.69488 0 0 0 # C_R

277 1 5 0.1652 20.79218 17.95582 1.15727 0 0 0 # H_

278 1 3 -0.4677 20.19804 5.63396 16.37749 0 0 0 # O_2

279 1 4 -0.1413 20.22387 5.60813 13.61088 0 0 0 # C_R

280 1 5 0.1652 20.79218 5.03982 14.07327 0 0 0 # H_

281 1 3 -0.4677 5.63396 20.19804 16.37749 0 0 0 # O_2

282 1 4 -0.1413 5.60813 20.22387 13.61088 0 0 0 # C_R

283 1 5 0.1652 5.03982 20.79218 14.07327 0 0 0 # H_

284 1 3 -0.4677 20.19804 7.28204 22.37051 0 0 0 # O_2

285 1 4 -0.1413 20.22387 7.30787 25.13712 0 0 0 # C_R

286 1 5 0.1652 20.79218 7.87618 24.67473 0 0 0 # H_

287 1 3 -0.4677 20.19804 20.19804 9.45451 0 0 0 # O_2

288 1 4 -0.1413 20.22387 20.22387 12.22112 0 0 0 # C_R

289 1 5 0.1652 20.79218 20.79218 11.75873 0 0 0 # H_

290 1 3 -0.4677 5.63396 18.54996 22.37051 0 0 0 # O_2

291 1 4 -0.1413 5.60813 18.52413 25.13712 0 0 0 # C_R

292 1 5 0.1652 5.03982 17.95582 24.67473 0 0 0 # H_

293 1 3 -0.4677 18.54996 22.37051 5.63396 0 0 0 # O_2

294 1 4 -0.1413 18.52413 25.13712 5.60813 0 0 0 # C_R

295 1 5 0.1652 17.95582 24.67473 5.03982 0 0 0 # H_

296 1 3 -0.4677 18.54996 9.45451 18.54996 0 0 0 # O_2

297 1 4 -0.1413 18.52413 12.22112 18.52413 0 0 0 # C_R

298 1 5 0.1652 17.95582 11.75873 17.95582 0 0 0 # H_

299 1 3 -0.4677 7.28204 22.37051 20.19804 0 0 0 # O_2

300 1 4 -0.1413 7.30787 25.13712 20.22387 0 0 0 # C_R

301 1 5 0.1652 7.87618 24.67473 20.79218 0 0 0 # H_

302 1 3 -0.4677 7.28204 16.37749 18.54996 0 0 0 # O_2

303 1 4 -0.1413 7.30787 13.61088 18.52413 0 0 0 # C_R

304 1 5 0.1652 7.87618 14.07327 17.95582 0 0 0 # H_

305 1 3 -0.4677 18.54996 3.46149 20.19804 0 0 0 # O_2

306 1 4 -0.1413 18.52413 0.69488 20.22387 0 0 0 # C_R

307 1 5 0.1652 17.95582 1.15727 20.79218 0 0 0 # H_

308 1 3 -0.4677 18.54996 16.37749 7.28204 0 0 0 # O_2

309 1 4 -0.1413 18.52413 13.61088 7.30787 0 0 0 # C_R

310 1 5 0.1652 17.95582 14.07327 7.87618 0 0 0 # H_

311 1 3 -0.4677 22.37051 20.19804 7.28204 0 0 0 # O_2

312 1 4 -0.1413 25.13712 20.22387 7.30787 0 0 0 # C_R

313 1 5 0.1652 24.67473 20.79218 7.87618 0 0 0 # H_

314 1 3 -0.4677 9.45451 20.19804 20.19804 0 0 0 # O_2

315 1 4 -0.1413 12.22112 20.22387 20.22387 0 0 0 # C_R

316 1 5 0.1652 11.75873 20.79218 20.79218 0 0 0 # H_

317 1 3 -0.4677 22.37051 5.63396 18.54996 0 0 0 # O_2

318 1 4 -0.1413 25.13712 5.60813 18.52413 0 0 0 # C_R

319 1 5 0.1652 24.67473 5.03982 17.95582 0 0 0 # H_

320 1 3 -0.4677 3.46149 20.19804 18.54996 0 0 0 # O_2

321 1 4 -0.1413 0.69488 20.22387 18.52413 0 0 0 # C_R

322 1 5 0.1652 1.15727 20.79218 17.95582 0 0 0 # H_

323 1 3 -0.4677 16.37749 20.19804 5.63396 0 0 0 # O_2

324 1 4 -0.1413 13.61088 20.22387 5.60813 0 0 0 # C_R

325 1 5 0.1652 14.07327 20.79218 5.03982 0 0 0 # H_

326 1 3 -0.4677 16.37749 5.63396 20.19804 0 0 0 # O_2

327 1 4 -0.1413 13.61088 5.60813 20.22387 0 0 0 # C_R

328 1 5 0.1652 14.07327 5.03982 20.79218 0 0 0 # H_

329 1 4 0.5937 6.458 6.458 2.8751 0 0 0 # C_R

330 1 4 0.0273 6.458 6.458 1.38976 0 0 0 # C_R

331 1 4 0.5937 6.458 19.374 15.7911 0 0 0 # C_R

332 1 4 0.0273 6.458 19.374 14.30576 0 0 0 # C_R

333 1 4 0.5937 19.374 19.374 2.8751 0 0 0 # C_R

334 1 4 0.0273 19.374 19.374 1.38976 0 0 0 # C_R

335 1 4 0.5937 19.374 6.458 15.7911 0 0 0 # C_R

336 1 4 0.0273 19.374 6.458 14.30576 0 0 0 # C_R

337 1 4 0.5937 19.374 6.458 22.9569 0 0 0 # C_R

338 1 4 0.0273 19.374 6.458 24.44224 0 0 0 # C_R

339 1 4 0.5937 19.374 19.374 10.0409 0 0 0 # C_R

340 1 4 0.0273 19.374 19.374 11.52624 0 0 0 # C_R

341 1 4 0.5937 6.458 6.458 10.0409 0 0 0 # C_R

342 1 4 0.0273 6.458 6.458 11.52624 0 0 0 # C_R

343 1 4 0.5937 6.458 19.374 22.9569 0 0 0 # C_R

344 1 4 0.0273 6.458 19.374 24.44224 0 0 0 # C_R

345 1 4 0.5937 2.8751 6.458 6.458 0 0 0 # C_R

346 1 4 0.0273 1.38976 6.458 6.458 0 0 0 # C_R

347 1 4 0.5937 15.7911 6.458 19.374 0 0 0 # C_R

348 1 4 0.0273 14.30576 6.458 19.374 0 0 0 # C_R

349 1 4 0.5937 2.8751 19.374 19.374 0 0 0 # C_R

350 1 4 0.0273 1.38976 19.374 19.374 0 0 0 # C_R

351 1 4 0.5937 15.7911 19.374 6.458 0 0 0 # C_R

352 1 4 0.0273 14.30576 19.374 6.458 0 0 0 # C_R

353 1 4 0.5937 22.9569 19.374 6.458 0 0 0 # C_R

354 1 4 0.0273 24.44224 19.374 6.458 0 0 0 # C_R

355 1 4 0.5937 10.0409 19.374 19.374 0 0 0 # C_R

356 1 4 0.0273 11.52624 19.374 19.374 0 0 0 # C_R

357 1 4 0.5937 10.0409 6.458 6.458 0 0 0 # C_R

358 1 4 0.0273 11.52624 6.458 6.458 0 0 0 # C_R

359 1 4 0.5937 22.9569 6.458 19.374 0 0 0 # C_R

360 1 4 0.0273 24.44224 6.458 19.374 0 0 0 # C_R

361 1 4 0.5937 6.458 2.8751 6.458 0 0 0 # C_R

362 1 4 0.0273 6.458 1.38976 6.458 0 0 0 # C_R

363 1 4 0.5937 6.458 15.7911 19.374 0 0 0 # C_R

364 1 4 0.0273 6.458 14.30576 19.374 0 0 0 # C_R

365 1 4 0.5937 19.374 2.8751 19.374 0 0 0 # C_R

366 1 4 0.0273 19.374 1.38976 19.374 0 0 0 # C_R

367 1 4 0.5937 19.374 15.7911 6.458 0 0 0 # C_R

368 1 4 0.0273 19.374 14.30576 6.458 0 0 0 # C_R

369 1 4 0.5937 6.458 22.9569 19.374 0 0 0 # C_R

370 1 4 0.0273 6.458 24.44224 19.374 0 0 0 # C_R

371 1 4 0.5937 6.458 10.0409 6.458 0 0 0 # C_R

372 1 4 0.0273 6.458 11.52624 6.458 0 0 0 # C_R

373 1 4 0.5937 19.374 22.9569 6.458 0 0 0 # C_R

374 1 4 0.0273 19.374 24.44224 6.458 0 0 0 # C_R

375 1 4 0.5937 19.374 10.0409 19.374 0 0 0 # C_R

376 1 4 0.0273 19.374 11.52624 19.374 0 0 0 # C_R

377 1 4 0.5937 6.458 6.458 22.9569 0 0 0 # C_R

378 1 4 0.0273 6.458 6.458 24.44224 0 0 0 # C_R

379 1 4 0.5937 6.458 19.374 10.0409 0 0 0 # C_R

380 1 4 0.0273 6.458 19.374 11.52624 0 0 0 # C_R

381 1 4 0.5937 19.374 19.374 22.9569 0 0 0 # C_R

382 1 4 0.0273 19.374 19.374 24.44224 0 0 0 # C_R

383 1 4 0.5937 19.374 6.458 10.0409 0 0 0 # C_R

384 1 4 0.0273 19.374 6.458 11.52624 0 0 0 # C_R

385 1 4 0.5937 6.458 19.374 2.8751 0 0 0 # C_R

386 1 4 0.0273 6.458 19.374 1.38976 0 0 0 # C_R

387 1 4 0.5937 6.458 6.458 15.7911 0 0 0 # C_R

388 1 4 0.0273 6.458 6.458 14.30576 0 0 0 # C_R

389 1 4 0.5937 19.374 6.458 2.8751 0 0 0 # C_R

390 1 4 0.0273 19.374 6.458 1.38976 0 0 0 # C_R

391 1 4 0.5937 19.374 19.374 15.7911 0 0 0 # C_R

392 1 4 0.0273 19.374 19.374 14.30576 0 0 0 # C_R

393 1 4 0.5937 6.458 2.8751 19.374 0 0 0 # C_R

394 1 4 0.0273 6.458 1.38976 19.374 0 0 0 # C_R

395 1 4 0.5937 6.458 15.7911 6.458 0 0 0 # C_R

396 1 4 0.0273 6.458 14.30576 6.458 0 0 0 # C_R

397 1 4 0.5937 19.374 2.8751 6.458 0 0 0 # C_R

398 1 4 0.0273 19.374 1.38976 6.458 0 0 0 # C_R

399 1 4 0.5937 19.374 15.7911 19.374 0 0 0 # C_R

400 1 4 0.0273 19.374 14.30576 19.374 0 0 0 # C_R

401 1 4 0.5937 19.374 22.9569 19.374 0 0 0 # C_R

402 1 4 0.0273 19.374 24.44224 19.374 0 0 0 # C_R

403 1 4 0.5937 19.374 10.0409 6.458 0 0 0 # C_R

404 1 4 0.0273 19.374 11.52624 6.458 0 0 0 # C_R

405 1 4 0.5937 6.458 22.9569 6.458 0 0 0 # C_R

406 1 4 0.0273 6.458 24.44224 6.458 0 0 0 # C_R

407 1 4 0.5937 6.458 10.0409 19.374 0 0 0 # C_R

408 1 4 0.0273 6.458 11.52624 19.374 0 0 0 # C_R

409 1 4 0.5937 2.8751 6.458 19.374 0 0 0 # C_R

410 1 4 0.0273 1.38976 6.458 19.374 0 0 0 # C_R

411 1 4 0.5937 15.7911 6.458 6.458 0 0 0 # C_R

412 1 4 0.0273 14.30576 6.458 6.458 0 0 0 # C_R

413 1 4 0.5937 2.8751 19.374 6.458 0 0 0 # C_R

414 1 4 0.0273 1.38976 19.374 6.458 0 0 0 # C_R

415 1 4 0.5937 15.7911 19.374 19.374 0 0 0 # C_R

416 1 4 0.0273 14.30576 19.374 19.374 0 0 0 # C_R

417 1 4 0.5937 22.9569 6.458 6.458 0 0 0 # C_R

418 1 4 0.0273 24.44224 6.458 6.458 0 0 0 # C_R

419 1 4 0.5937 10.0409 6.458 19.374 0 0 0 # C_R

420 1 4 0.0273 11.52624 6.458 19.374 0 0 0 # C_R

421 1 4 0.5937 22.9569 19.374 19.374 0 0 0 # C_R

422 1 4 0.0273 24.44224 19.374 19.374 0 0 0 # C_R

423 1 4 0.5937 10.0409 19.374 6.458 0 0 0 # C_R

424 1 4 0.0273 11.52624 19.374 6.458 0 0 0 # C_R

**2.#data file for IRMOF-16**

904 atoms

5 atom types

0.000119338 42.980719338 xlo xhi

0.000121240 42.980721240 ylo yhi

0.000119338 42.980719338 zlo zhi

Atoms # full (The fourth column is charge)

1 1 1 0.4584 9.62808 9.62808 9.62808 0 0 0 # Zn3+2

2 1 2 -0.1879 10.74515 10.74515 10.74515 0 0 0 # O_3

3 1 3 -0.4663 10.01018 10.01018 7.77906 0 0 0 # O_2

4 1 4 0.5957 10.74515 10.74515 7.11114 0 0 0 # C_R

5 1 4 0.0296 10.74515 10.74515 5.73103 0 0 0 # C_R

6 1 4 -0.1412 9.88812 9.88812 5.04807 0 0 0 # C_R

7 1 4 -0.1382 9.88984 9.88984 3.6512 0 0 0 # C_R

8 1 4 0.0277 10.74515 10.74515 2.94202 0 0 0 # C_R

9 1 4 0.0292 10.74515 10.74515 1.41621 0 0 0 # C_R

10 1 1 0.4583 11.86308 11.86308 9.62808 0 0 0 # Zn3+2

11 1 3 -0.4663 11.48012 11.48012 7.77906 0 0 0 # O_2

12 1 4 -0.1416 11.60304 11.60304 5.04807 0 0 0 # C_R

13 1 4 -0.138 11.60003 11.60003 3.6512 0 0 0 # C_R

14 1 1 0.4583 11.86308 9.62808 11.86308 0 0 0 # Zn3+2

15 1 3 -0.4664 11.48012 10.01018 13.71081 0 0 0 # O_2

16 1 4 0.5955 10.74515 10.74515 14.37916 0 0 0 # C_R

17 1 4 0.0296 10.74515 10.74515 15.75884 0 0 0 # C_R

18 1 4 -0.1415 11.60304 9.88812 16.4418 0 0 0 # C_R

19 1 4 -0.1384 11.60003 9.88984 17.8391 0 0 0 # C_R

20 1 4 0.0277 10.74515 10.74515 18.54785 0 0 0 # C_R

21 1 4 0.0291 10.74515 10.74515 20.07409 0 0 0 # C_R

22 1 4 -0.1279 11.9572 10.74515 20.788 0 0 0 # C_R

23 1 1 0.4583 9.62808 11.86308 11.86308 0 0 0 # Zn3+2

24 1 3 -0.4662 10.01018 11.48012 13.71081 0 0 0 # O_2

25 1 4 -0.1414 9.88812 11.60304 16.4418 0 0 0 # C_R

26 1 4 -0.1379 9.88984 11.60003 17.8391 0 0 0 # C_R

27 1 4 -0.1277 9.5331 10.74515 20.788 0 0 0 # C_R

28 1 3 -0.4662 7.77906 10.01018 10.01018 0 0 0 # O_2

29 1 4 0.5957 7.11114 10.74515 10.74515 0 0 0 # C_R

30 1 4 0.0296 5.73103 10.74515 10.74515 0 0 0 # C_R

31 1 4 -0.141 5.04807 9.88812 9.88812 0 0 0 # C_R

32 1 4 -0.1381 3.6512 9.88984 9.88984 0 0 0 # C_R

33 1 4 0.0277 2.94202 10.74515 10.74515 0 0 0 # C_R

34 1 4 0.0292 1.41621 10.74515 10.74515 0 0 0 # C_R

35 1 4 -0.1282 0.70316 9.5331 10.74515 0 0 0 # C_R

36 1 3 -0.4664 7.77906 11.48012 11.48012 0 0 0 # O_2

37 1 4 -0.1418 5.04807 11.60304 11.60304 0 0 0 # C_R

38 1 4 -0.1381 3.6512 11.60003 11.60003 0 0 0 # C_R

39 1 4 -0.1281 0.70316 11.9572 10.74515 0 0 0 # C_R

40 1 3 -0.4663 13.71081 11.48012 10.01018 0 0 0 # O_2

41 1 4 0.5955 14.37916 10.74515 10.74515 0 0 0 # C_R

42 1 4 0.0296 15.75884 10.74515 10.74515 0 0 0 # C_R

43 1 4 -0.1413 16.4418 11.60304 9.88812 0 0 0 # C_R

44 1 4 -0.1383 17.8391 11.60003 9.88984 0 0 0 # C_R

45 1 4 0.0277 18.54785 10.74515 10.74515 0 0 0 # C_R

46 1 4 0.0291 20.07409 10.74515 10.74515 0 0 0 # C_R

47 1 3 -0.4662 13.71081 10.01018 11.48012 0 0 0 # O_2

48 1 4 -0.1416 16.4418 9.88812 11.60304 0 0 0 # C_R

49 1 4 -0.138 17.8391 9.88984 11.60003 0 0 0 # C_R

50 1 3 -0.4664 10.01018 7.77906 10.01018 0 0 0 # O_2

51 1 4 0.5957 10.74515 7.11114 10.74515 0 0 0 # C_R

52 1 4 0.0296 10.74515 5.73103 10.74515 0 0 0 # C_R

53 1 4 -0.1414 9.88812 5.04807 9.88812 0 0 0 # C_R

54 1 4 -0.1384 9.88984 3.6512 9.88984 0 0 0 # C_R

55 1 4 0.0277 10.74515 2.94202 10.74515 0 0 0 # C_R

56 1 4 0.0292 10.74515 1.41621 10.74515 0 0 0 # C_R

57 1 4 -0.1283 10.74515 0.70316 9.5331 0 0 0 # C_R

58 1 3 -0.4662 11.48012 7.77906 11.48012 0 0 0 # O_2

59 1 4 -0.1414 11.60304 5.04807 11.60304 0 0 0 # C_R

60 1 4 -0.1378 11.60003 3.6512 11.60003 0 0 0 # C_R

61 1 4 -0.128 10.74515 0.70316 11.9572 0 0 0 # C_R

62 1 3 -0.4663 10.01018 13.71081 11.48012 0 0 0 # O_2

63 1 4 0.5955 10.74515 14.37916 10.74515 0 0 0 # C_R

64 1 4 0.0296 10.74515 15.75884 10.74515 0 0 0 # C_R

65 1 4 -0.1413 9.88812 16.4418 11.60304 0 0 0 # C_R

66 1 4 -0.1382 9.88984 17.8391 11.60003 0 0 0 # C_R

67 1 4 0.0277 10.74515 18.54785 10.74515 0 0 0 # C_R

68 1 4 0.0291 10.74515 20.07409 10.74515 0 0 0 # C_R

69 1 3 -0.4662 11.48012 13.71081 10.01018 0 0 0 # O_2

70 1 4 -0.1416 11.60304 16.4418 9.88812 0 0 0 # C_R

71 1 4 -0.1381 11.60003 17.8391 9.88984 0 0 0 # C_R

72 1 4 -0.128 10.74515 11.9572 0.70316 0 0 0 # C_R

73 1 4 -0.1283 10.74515 9.5331 0.70316 0 0 0 # C_R

74 1 4 -0.1277 11.9572 20.788 10.74515 0 0 0 # C_R

75 1 4 -0.128 9.5331 20.788 10.74515 0 0 0 # C_R

76 1 4 -0.1278 20.788 10.74515 9.5331 0 0 0 # C_R

77 1 4 -0.1279 20.788 10.74515 11.9572 0 0 0 # C_R

78 1 2 -0.1878 10.74515 10.74515 32.23545 0 0 0 # O_3

79 1 4 0.5954 10.74515 10.74515 28.60101 0 0 0 # C_R

80 1 4 0.0297 10.74515 10.74515 27.22219 0 0 0 # C_R

81 1 4 0.0278 10.74515 10.74515 24.43189 0 0 0 # C_R

82 1 4 0.0289 10.74515 10.74515 22.90694 0 0 0 # C_R

83 1 4 -0.1281 9.5331 10.74515 22.19303 0 0 0 # C_R

84 1 4 -0.1281 11.9572 10.74515 22.19303 0 0 0 # C_R

85 1 4 0.5957 10.74515 10.74515 35.86903 0 0 0 # C_R

86 1 4 0.0297 10.74515 10.74515 37.24914 0 0 0 # C_R

87 1 4 0.028 10.74515 10.74515 40.03901 0 0 0 # C_R

88 1 4 0.0294 10.74515 10.74515 41.56396 0 0 0 # C_R

89 1 4 0.5957 7.11114 10.74515 32.23545 0 0 0 # C_R

90 1 4 0.0296 5.73103 10.74515 32.23545 0 0 0 # C_R

91 1 4 0.028 2.94202 10.74515 32.23545 0 0 0 # C_R

92 1 4 0.0294 1.41621 10.74515 32.23545 0 0 0 # C_R

93 1 4 -0.1281 0.70316 9.5331 32.23545 0 0 0 # C_R

94 1 4 -0.1281 0.70316 11.9572 32.23545 0 0 0 # C_R

95 1 4 0.5955 14.37916 10.74515 32.23545 0 0 0 # C_R

96 1 4 0.0296 15.75884 10.74515 32.23545 0 0 0 # C_R

97 1 4 0.028 18.54785 10.74515 32.23545 0 0 0 # C_R

98 1 4 0.0293 20.07409 10.74515 32.23545 0 0 0 # C_R

99 1 4 0.5957 10.74515 7.11114 32.23545 0 0 0 # C_R

100 1 4 0.0296 10.74515 5.73103 32.23545 0 0 0 # C_R

101 1 4 0.028 10.74515 2.94202 32.23545 0 0 0 # C_R

102 1 4 0.0294 10.74515 1.41621 32.23545 0 0 0 # C_R

103 1 4 -0.1281 10.74515 0.70316 31.02297 0 0 0 # C_R

104 1 4 -0.1281 10.74515 0.70316 33.44793 0 0 0 # C_R

105 1 4 0.5955 10.74515 14.37916 32.23545 0 0 0 # C_R

106 1 4 0.0296 10.74515 15.75884 32.23545 0 0 0 # C_R

107 1 4 0.028 10.74515 18.54785 32.23545 0 0 0 # C_R

108 1 4 0.0293 10.74515 20.07409 32.23545 0 0 0 # C_R

109 1 1 0.4584 9.62808 9.62808 33.35295 0 0 0 # Zn3+2

110 1 3 -0.4663 10.01018 10.01018 35.20111 0 0 0 # O_2

111 1 4 -0.1415 9.88812 9.88812 37.93296 0 0 0 # C_R

112 1 4 -0.1382 9.88984 9.88984 39.32897 0 0 0 # C_R

113 1 4 -0.1281 10.74515 9.5331 42.27787 0 0 0 # C_R

114 1 1 0.4582 11.86308 11.86308 33.35295 0 0 0 # Zn3+2

115 1 3 -0.4663 11.48012 11.48012 35.20111 0 0 0 # O_2

116 1 4 -0.1417 11.60304 11.60304 37.93296 0 0 0 # C_R

117 1 4 -0.1381 11.60003 11.60003 39.32897 0 0 0 # C_R

118 1 4 -0.1279 10.74515 11.9572 42.27787 0 0 0 # C_R

119 1 1 0.4584 9.62808 11.86308 31.11795 0 0 0 # Zn3+2

120 1 3 -0.466 10.01018 11.48012 29.26979 0 0 0 # O_2

121 1 4 -0.1414 9.88812 11.60304 26.53794 0 0 0 # C_R

122 1 4 -0.138 9.88984 11.60003 25.14193 0 0 0 # C_R

123 1 1 0.4584 11.86308 9.62808 31.11795 0 0 0 # Zn3+2

124 1 3 -0.4662 11.48012 10.01018 29.26979 0 0 0 # O_2

125 1 4 -0.1418 11.60304 9.88812 26.53794 0 0 0 # C_R

126 1 4 -0.1384 11.60003 9.88984 25.14193 0 0 0 # C_R

127 1 3 -0.4665 10.01018 7.77906 32.96999 0 0 0 # O_2

128 1 4 -0.1417 9.88812 5.04807 33.09291 0 0 0 # C_R

129 1 4 -0.1384 9.88984 3.6512 33.09119 0 0 0 # C_R

130 1 3 -0.4662 11.48012 7.77906 31.50005 0 0 0 # O_2

131 1 4 -0.1414 11.60304 5.04807 31.37799 0 0 0 # C_R

132 1 4 -0.1379 11.60003 3.6512 31.38014 0 0 0 # C_R

133 1 3 -0.4663 11.48012 13.71081 32.96999 0 0 0 # O_2

134 1 4 -0.1416 11.60304 16.4418 33.09291 0 0 0 # C_R

135 1 4 -0.1382 11.60003 17.8391 33.09119 0 0 0 # C_R

136 1 4 -0.1277 11.9572 20.788 32.23545 0 0 0 # C_R

137 1 3 -0.4662 10.01018 13.71081 31.50005 0 0 0 # O_2

138 1 4 -0.1415 9.88812 16.4418 31.37799 0 0 0 # C_R

139 1 4 -0.1381 9.88984 17.8391 31.38014 0 0 0 # C_R

140 1 4 -0.1279 9.5331 20.788 32.23545 0 0 0 # C_R

141 1 3 -0.4663 7.77906 10.01018 32.96999 0 0 0 # O_2

142 1 4 -0.1413 5.04807 9.88812 33.09291 0 0 0 # C_R

143 1 4 -0.1381 3.6512 9.88984 33.09119 0 0 0 # C_R

144 1 3 -0.4664 7.77906 11.48012 31.50005 0 0 0 # O_2

145 1 4 -0.1418 5.04807 11.60304 31.37799 0 0 0 # C_R

146 1 4 -0.1383 3.6512 11.60003 31.38014 0 0 0 # C_R

147 1 3 -0.4662 13.71081 10.01018 31.50005 0 0 0 # O_2

148 1 4 -0.1415 16.4418 9.88812 31.37799 0 0 0 # C_R

149 1 4 -0.1381 17.8391 9.88984 31.38014 0 0 0 # C_R

150 1 4 -0.1279 20.788 10.74515 31.02297 0 0 0 # C_R

151 1 3 -0.4663 13.71081 11.48012 32.96999 0 0 0 # O_2

152 1 4 -0.1416 16.4418 11.60304 33.09291 0 0 0 # C_R

153 1 4 -0.1382 17.8391 11.60003 33.09119 0 0 0 # C_R

154 1 4 -0.1278 20.788 10.74515 33.44793 0 0 0 # C_R

155 1 2 -0.1878 10.74515 32.23545 10.74515 0 0 0 # O_3

156 1 4 0.5957 10.74515 32.23545 7.11114 0 0 0 # C_R

157 1 4 0.0296 10.74515 32.23545 5.73103 0 0 0 # C_R

158 1 4 0.028 10.74515 32.23545 2.94202 0 0 0 # C_R

159 1 4 0.0294 10.74515 32.23545 1.41621 0 0 0 # C_R

160 1 4 0.5955 10.74515 32.23545 14.37916 0 0 0 # C_R

161 1 4 0.0296 10.74515 32.23545 15.75884 0 0 0 # C_R

162 1 4 0.028 10.74515 32.23545 18.54785 0 0 0 # C_R

163 1 4 0.0293 10.74515 32.23545 20.07409 0 0 0 # C_R

164 1 4 -0.1279 11.9572 32.23545 20.788 0 0 0 # C_R

165 1 4 -0.1277 9.5331 32.23545 20.788 0 0 0 # C_R

166 1 4 0.5957 7.11114 32.23545 10.74515 0 0 0 # C_R

167 1 4 0.0296 5.73103 32.23545 10.74515 0 0 0 # C_R

168 1 4 0.028 2.94202 32.23545 10.74515 0 0 0 # C_R

169 1 4 0.0294 1.41621 32.23545 10.74515 0 0 0 # C_R

170 1 4 -0.1282 0.70316 31.02297 10.74515 0 0 0 # C_R

171 1 4 -0.128 0.70316 33.44793 10.74515 0 0 0 # C_R

172 1 4 0.5955 14.37916 32.23545 10.74515 0 0 0 # C_R

173 1 4 0.0296 15.75884 32.23545 10.74515 0 0 0 # C_R

174 1 4 0.028 18.54785 32.23545 10.74515 0 0 0 # C_R

175 1 4 0.0293 20.07409 32.23545 10.74515 0 0 0 # C_R

176 1 4 0.5954 10.74515 28.60101 10.74515 0 0 0 # C_R

177 1 4 0.0297 10.74515 27.22219 10.74515 0 0 0 # C_R

178 1 4 0.0278 10.74515 24.43189 10.74515 0 0 0 # C_R

179 1 4 0.0289 10.74515 22.90694 10.74515 0 0 0 # C_R

180 1 4 0.5957 10.74515 35.86903 10.74515 0 0 0 # C_R

181 1 4 0.0297 10.74515 37.24914 10.74515 0 0 0 # C_R

182 1 4 0.028 10.74515 40.03901 10.74515 0 0 0 # C_R

183 1 4 0.0294 10.74515 41.56396 10.74515 0 0 0 # C_R

184 1 4 -0.128 10.74515 42.27787 11.9572 0 0 0 # C_R

185 1 4 -0.1281 10.74515 42.27787 9.5331 0 0 0 # C_R

186 1 1 0.4584 9.62808 31.11795 11.86308 0 0 0 # Zn3+2

187 1 3 -0.4661 10.01018 31.50005 13.71081 0 0 0 # O_2

188 1 4 -0.1413 9.88812 31.37799 16.4418 0 0 0 # C_R

189 1 4 -0.138 9.88984 31.38014 17.8391 0 0 0 # C_R

190 1 1 0.4582 11.86308 33.35295 11.86308 0 0 0 # Zn3+2

191 1 3 -0.4664 11.48012 32.96999 13.71081 0 0 0 # O_2

192 1 4 -0.1418 11.60304 33.09291 16.4418 0 0 0 # C_R

193 1 4 -0.1383 11.60003 33.09119 17.8391 0 0 0 # C_R

194 1 1 0.4584 9.62808 33.35295 9.62808 0 0 0 # Zn3+2

195 1 3 -0.4664 10.01018 32.96999 7.77906 0 0 0 # O_2

196 1 4 -0.1415 9.88812 33.09291 5.04807 0 0 0 # C_R

197 1 4 -0.1382 9.88984 33.09119 3.6512 0 0 0 # C_R

198 1 4 -0.1282 10.74515 33.44793 0.70316 0 0 0 # C_R

199 1 1 0.4584 11.86308 31.11795 9.62808 0 0 0 # Zn3+2

200 1 3 -0.4663 11.48012 31.50005 7.77906 0 0 0 # O_2

201 1 4 -0.1416 11.60304 31.37799 5.04807 0 0 0 # C_R

202 1 4 -0.1382 11.60003 31.38014 3.6512 0 0 0 # C_R

203 1 4 -0.128 10.74515 31.02297 0.70316 0 0 0 # C_R

204 1 3 -0.4661 10.01018 29.26979 11.48012 0 0 0 # O_2

205 1 4 -0.1416 9.88812 26.53794 11.60304 0 0 0 # C_R

206 1 4 -0.1381 9.88984 25.14193 11.60003 0 0 0 # C_R

207 1 4 -0.1282 9.5331 22.19303 10.74515 0 0 0 # C_R

208 1 3 -0.4661 11.48012 29.26979 10.01018 0 0 0 # O_2

209 1 4 -0.1416 11.60304 26.53794 9.88812 0 0 0 # C_R

210 1 4 -0.1382 11.60003 25.14193 9.88984 0 0 0 # C_R

211 1 4 -0.128 11.9572 22.19303 10.74515 0 0 0 # C_R

212 1 3 -0.4662 11.48012 35.20111 11.48012 0 0 0 # O_2

213 1 4 -0.1415 11.60304 37.93296 11.60304 0 0 0 # C_R

214 1 4 -0.1379 11.60003 39.32897 11.60003 0 0 0 # C_R

215 1 3 -0.4664 10.01018 35.20111 10.01018 0 0 0 # O_2

216 1 4 -0.1417 9.88812 37.93296 9.88812 0 0 0 # C_R

217 1 4 -0.1384 9.88984 39.32897 9.88984 0 0 0 # C_R

218 1 3 -0.4664 7.77906 31.50005 11.48012 0 0 0 # O_2

219 1 4 -0.1418 5.04807 31.37799 11.60304 0 0 0 # C_R

220 1 4 -0.1383 3.6512 31.38014 11.60003 0 0 0 # C_R

221 1 3 -0.4663 7.77906 32.96999 10.01018 0 0 0 # O_2

222 1 4 -0.1413 5.04807 33.09291 9.88812 0 0 0 # C_R

223 1 4 -0.1381 3.6512 33.09119 9.88984 0 0 0 # C_R

224 1 3 -0.4662 13.71081 31.50005 10.01018 0 0 0 # O_2

225 1 4 -0.1415 16.4418 31.37799 9.88812 0 0 0 # C_R

226 1 4 -0.1382 17.8391 31.38014 9.88984 0 0 0 # C_R

227 1 4 -0.1277 20.788 32.23545 9.5331 0 0 0 # C_R

228 1 3 -0.4663 13.71081 32.96999 11.48012 0 0 0 # O_2

229 1 4 -0.1416 16.4418 33.09291 11.60304 0 0 0 # C_R

230 1 4 -0.1381 17.8391 33.09119 11.60003 0 0 0 # C_R

231 1 4 -0.1279 20.788 32.23545 11.9572 0 0 0 # C_R

232 1 1 0.4585 9.62808 31.11795 31.11795 0 0 0 # Zn3+2

233 1 2 -0.1878 10.74515 32.23545 32.23545 0 0 0 # O_3

234 1 3 -0.466 10.01018 31.50005 29.26979 0 0 0 # O_2

235 1 4 0.5954 10.74515 32.23545 28.60101 0 0 0 # C_R

236 1 4 0.0296 10.74515 32.23545 27.22219 0 0 0 # C_R

237 1 4 -0.141 9.88812 31.37799 26.53794 0 0 0 # C_R

238 1 4 -0.1381 9.88984 31.38014 25.14193 0 0 0 # C_R

239 1 4 0.0276 10.74515 32.23545 24.43189 0 0 0 # C_R

240 1 4 0.0287 10.74515 32.23545 22.90694 0 0 0 # C_R

241 1 4 -0.1282 9.5331 32.23545 22.19303 0 0 0 # C_R

242 1 1 0.4583 11.86308 33.35295 31.11795 0 0 0 # Zn3+2

243 1 3 -0.4663 11.48012 32.96999 29.26979 0 0 0 # O_2

244 1 4 -0.1418 11.60304 33.09291 26.53794 0 0 0 # C_R

245 1 4 -0.1383 11.60003 33.09119 25.14193 0 0 0 # C_R

246 1 4 -0.1281 11.9572 32.23545 22.19303 0 0 0 # C_R

247 1 1 0.4583 11.86308 31.11795 33.35295 0 0 0 # Zn3+2

248 1 3 -0.4663 11.48012 31.50005 35.20111 0 0 0 # O_2

249 1 4 0.5957 10.74515 32.23545 35.86903 0 0 0 # C_R

250 1 4 0.0296 10.74515 32.23545 37.24914 0 0 0 # C_R

251 1 4 -0.1413 11.60304 31.37799 37.93296 0 0 0 # C_R

252 1 4 -0.1383 11.60003 31.38014 39.32897 0 0 0 # C_R

253 1 4 0.0278 10.74515 32.23545 40.03901 0 0 0 # C_R

254 1 4 0.0293 10.74515 32.23545 41.56396 0 0 0 # C_R

255 1 1 0.4583 9.62808 33.35295 33.35295 0 0 0 # Zn3+2

256 1 3 -0.4664 10.01018 32.96999 35.20111 0 0 0 # O_2

257 1 4 -0.1415 9.88812 33.09291 37.93296 0 0 0 # C_R

258 1 4 -0.1381 9.88984 33.09119 39.32897 0 0 0 # C_R

259 1 3 -0.4663 7.77906 31.50005 31.50005 0 0 0 # O_2

260 1 4 0.5957 7.11114 32.23545 32.23545 0 0 0 # C_R

261 1 4 0.0296 5.73103 32.23545 32.23545 0 0 0 # C_R

262 1 4 -0.1415 5.04807 31.37799 31.37799 0 0 0 # C_R

263 1 4 -0.1384 3.6512 31.38014 31.38014 0 0 0 # C_R

264 1 4 0.0278 2.94202 32.23545 32.23545 0 0 0 # C_R

265 1 4 0.0293 1.41621 32.23545 32.23545 0 0 0 # C_R

266 1 4 -0.1283 0.70316 31.02297 32.23545 0 0 0 # C_R

267 1 3 -0.4663 7.77906 32.96999 32.96999 0 0 0 # O_2

268 1 4 -0.1413 5.04807 33.09291 33.09291 0 0 0 # C_R

269 1 4 -0.138 3.6512 33.09119 33.09119 0 0 0 # C_R

270 1 4 -0.128 0.70316 33.44793 32.23545 0 0 0 # C_R

271 1 3 -0.4663 13.71081 32.96999 31.50005 0 0 0 # O_2

272 1 4 0.5955 14.37916 32.23545 32.23545 0 0 0 # C_R

273 1 4 0.0295 15.75884 32.23545 32.23545 0 0 0 # C_R

274 1 4 -0.1413 16.4418 33.09291 31.37799 0 0 0 # C_R

275 1 4 -0.1382 17.8391 33.09119 31.38014 0 0 0 # C_R

276 1 4 0.0278 18.54785 32.23545 32.23545 0 0 0 # C_R

277 1 4 0.0292 20.07409 32.23545 32.23545 0 0 0 # C_R

278 1 3 -0.4662 13.71081 31.50005 32.96999 0 0 0 # O_2

279 1 4 -0.1416 16.4418 31.37799 33.09291 0 0 0 # C_R

280 1 4 -0.1382 17.8391 31.38014 33.09119 0 0 0 # C_R

281 1 3 -0.4661 10.01018 29.26979 31.50005 0 0 0 # O_2

282 1 4 0.5954 10.74515 28.60101 32.23545 0 0 0 # C_R

283 1 4 0.0296 10.74515 27.22219 32.23545 0 0 0 # C_R

284 1 4 -0.1412 9.88812 26.53794 31.37799 0 0 0 # C_R

285 1 4 -0.1383 9.88984 25.14193 31.38014 0 0 0 # C_R

286 1 4 0.0276 10.74515 24.43189 32.23545 0 0 0 # C_R

287 1 4 0.0287 10.74515 22.90694 32.23545 0 0 0 # C_R

288 1 3 -0.4662 11.48012 29.26979 32.96999 0 0 0 # O_2

289 1 4 -0.1416 11.60304 26.53794 33.09291 0 0 0 # C_R

290 1 4 -0.1381 11.60003 25.14193 33.09119 0 0 0 # C_R

291 1 3 -0.4665 10.01018 35.20111 32.96999 0 0 0 # O_2

292 1 4 0.5957 10.74515 35.86903 32.23545 0 0 0 # C_R

293 1 4 0.0296 10.74515 37.24914 32.23545 0 0 0 # C_R

294 1 4 -0.1414 9.88812 37.93296 33.09291 0 0 0 # C_R

295 1 4 -0.1385 9.88984 39.32897 33.09119 0 0 0 # C_R

296 1 4 0.0278 10.74515 40.03901 32.23545 0 0 0 # C_R

297 1 4 0.0293 10.74515 41.56396 32.23545 0 0 0 # C_R

298 1 4 -0.1282 10.74515 42.27787 33.44793 0 0 0 # C_R

299 1 3 -0.4662 11.48012 35.20111 31.50005 0 0 0 # O_2

300 1 4 -0.1414 11.60304 37.93296 31.37799 0 0 0 # C_R

301 1 4 -0.1379 11.60003 39.32897 31.38014 0 0 0 # C_R

302 1 4 -0.128 10.74515 42.27787 31.02297 0 0 0 # C_R

303 1 4 -0.1281 10.74515 31.02297 42.27787 0 0 0 # C_R

304 1 4 -0.1281 10.74515 33.44793 42.27787 0 0 0 # C_R

305 1 4 -0.1283 9.5331 22.19303 32.23545 0 0 0 # C_R

306 1 4 -0.128 11.9572 22.19303 32.23545 0 0 0 # C_R

307 1 4 -0.128 20.788 32.23545 31.02297 0 0 0 # C_R

308 1 4 -0.1277 20.788 32.23545 33.44793 0 0 0 # C_R

309 1 2 -0.1878 32.23545 10.74515 10.74515 0 0 0 # O_3

310 1 4 0.5957 32.23545 10.74515 7.11114 0 0 0 # C_R

311 1 4 0.0296 32.23545 10.74515 5.73103 0 0 0 # C_R

312 1 4 0.028 32.23545 10.74515 2.94202 0 0 0 # C_R

313 1 4 0.0294 32.23545 10.74515 1.41621 0 0 0 # C_R

314 1 4 0.5955 32.23545 10.74515 14.37916 0 0 0 # C_R

315 1 4 0.0296 32.23545 10.74515 15.75884 0 0 0 # C_R

316 1 4 0.028 32.23545 10.74515 18.54785 0 0 0 # C_R

317 1 4 0.0293 32.23545 10.74515 20.07409 0 0 0 # C_R

318 1 4 -0.1278 33.44793 10.74515 20.788 0 0 0 # C_R

319 1 4 -0.1278 31.02297 10.74515 20.788 0 0 0 # C_R

320 1 4 0.5954 28.60101 10.74515 10.74515 0 0 0 # C_R

321 1 4 0.0297 27.22219 10.74515 10.74515 0 0 0 # C_R

322 1 4 0.0278 24.43189 10.74515 10.74515 0 0 0 # C_R

323 1 4 0.0289 22.90694 10.74515 10.74515 0 0 0 # C_R

324 1 4 0.5957 35.86903 10.74515 10.74515 0 0 0 # C_R

325 1 4 0.0297 37.24914 10.74515 10.74515 0 0 0 # C_R

326 1 4 0.028 40.03901 10.74515 10.74515 0 0 0 # C_R

327 1 4 0.0294 41.56396 10.74515 10.74515 0 0 0 # C_R

328 1 4 -0.1281 42.27787 11.9572 10.74515 0 0 0 # C_R

329 1 4 -0.128 42.27787 9.5331 10.74515 0 0 0 # C_R

330 1 4 0.5957 32.23545 7.11114 10.74515 0 0 0 # C_R

331 1 4 0.0296 32.23545 5.73103 10.74515 0 0 0 # C_R

332 1 4 0.028 32.23545 2.94202 10.74515 0 0 0 # C_R

333 1 4 0.0294 32.23545 1.41621 10.74515 0 0 0 # C_R

334 1 4 -0.1282 32.23545 0.70316 9.5331 0 0 0 # C_R

335 1 4 -0.128 32.23545 0.70316 11.9572 0 0 0 # C_R

336 1 4 0.5955 32.23545 14.37916 10.74515 0 0 0 # C_R

337 1 4 0.0296 32.23545 15.75884 10.74515 0 0 0 # C_R

338 1 4 0.028 32.23545 18.54785 10.74515 0 0 0 # C_R

339 1 4 0.0293 32.23545 20.07409 10.74515 0 0 0 # C_R

340 1 1 0.4584 31.11795 9.62808 11.86308 0 0 0 # Zn3+2

341 1 3 -0.4663 31.50005 10.01018 13.71081 0 0 0 # O_2

342 1 4 -0.1417 31.37799 9.88812 16.4418 0 0 0 # C_R

343 1 4 -0.1383 31.38014 9.88984 17.8391 0 0 0 # C_R

344 1 1 0.4582 33.35295 11.86308 11.86308 0 0 0 # Zn3+2

345 1 3 -0.4662 32.96999 11.48012 13.71081 0 0 0 # O_2

346 1 4 -0.1414 33.09291 11.60304 16.4418 0 0 0 # C_R

347 1 4 -0.1379 33.09119 11.60003 17.8391 0 0 0 # C_R

348 1 1 0.4584 31.11795 11.86308 9.62808 0 0 0 # Zn3+2

349 1 3 -0.4663 31.50005 11.48012 7.77906 0 0 0 # O_2

350 1 4 -0.1416 31.37799 11.60304 5.04807 0 0 0 # C_R

351 1 4 -0.1382 31.38014 11.60003 3.6512 0 0 0 # C_R

352 1 4 -0.128 32.23545 11.9572 0.70316 0 0 0 # C_R

353 1 1 0.4584 33.35295 9.62808 9.62808 0 0 0 # Zn3+2

354 1 3 -0.4664 32.96999 10.01018 7.77906 0 0 0 # O_2

355 1 4 -0.1415 33.09291 9.88812 5.04807 0 0 0 # C_R

356 1 4 -0.1382 33.09119 9.88984 3.6512 0 0 0 # C_R

357 1 4 -0.1282 32.23545 9.5331 0.70316 0 0 0 # C_R

358 1 3 -0.4662 31.50005 7.77906 11.48012 0 0 0 # O_2

359 1 4 -0.1414 31.37799 5.04807 11.60304 0 0 0 # C_R

360 1 4 -0.1379 31.38014 3.6512 11.60003 0 0 0 # C_R

361 1 3 -0.4665 32.96999 7.77906 10.01018 0 0 0 # O_2

362 1 4 -0.1417 33.09291 5.04807 9.88812 0 0 0 # C_R

363 1 4 -0.1384 33.09119 3.6512 9.88984 0 0 0 # C_R

364 1 3 -0.4663 32.96999 13.71081 11.48012 0 0 0 # O_2

365 1 4 -0.1416 33.09291 16.4418 11.60304 0 0 0 # C_R

366 1 4 -0.1381 33.09119 17.8391 11.60003 0 0 0 # C_R

367 1 4 -0.128 33.44793 20.788 10.74515 0 0 0 # C_R

368 1 3 -0.4662 31.50005 13.71081 10.01018 0 0 0 # O_2

369 1 4 -0.1415 31.37799 16.4418 9.88812 0 0 0 # C_R

370 1 4 -0.1382 31.38014 17.8391 9.88984 0 0 0 # C_R

371 1 4 -0.1277 31.02297 20.788 10.74515 0 0 0 # C_R

372 1 3 -0.4661 29.26979 10.01018 11.48012 0 0 0 # O_2

373 1 4 -0.1416 26.53794 9.88812 11.60304 0 0 0 # C_R

374 1 4 -0.1382 25.14193 9.88984 11.60003 0 0 0 # C_R

375 1 4 -0.1282 22.19303 10.74515 11.9572 0 0 0 # C_R

376 1 3 -0.4661 29.26979 11.48012 10.01018 0 0 0 # O_2

377 1 4 -0.1416 26.53794 11.60304 9.88812 0 0 0 # C_R

378 1 4 -0.1382 25.14193 11.60003 9.88984 0 0 0 # C_R

379 1 4 -0.128 22.19303 10.74515 9.5331 0 0 0 # C_R

380 1 3 -0.4662 35.20111 10.01018 10.01018 0 0 0 # O_2

381 1 4 -0.1413 37.93296 9.88812 9.88812 0 0 0 # C_R

382 1 4 -0.138 39.32897 9.88984 9.88984 0 0 0 # C_R

383 1 3 -0.4664 35.20111 11.48012 11.48012 0 0 0 # O_2

384 1 4 -0.1419 37.93296 11.60304 11.60304 0 0 0 # C_R

385 1 4 -0.1383 39.32897 11.60003 11.60003 0 0 0 # C_R

386 1 1 0.4585 31.11795 9.62808 31.11795 0 0 0 # Zn3+2

387 1 2 -0.1878 32.23545 10.74515 32.23545 0 0 0 # O_3

388 1 3 -0.4662 31.50005 10.01018 29.26979 0 0 0 # O_2

389 1 4 0.5954 32.23545 10.74515 28.60101 0 0 0 # C_R

390 1 4 0.0296 32.23545 10.74515 27.22219 0 0 0 # C_R

391 1 4 -0.1414 31.37799 9.88812 26.53794 0 0 0 # C_R

392 1 4 -0.1385 31.38014 9.88984 25.14193 0 0 0 # C_R

393 1 4 0.0276 32.23545 10.74515 24.43189 0 0 0 # C_R

394 1 4 0.0288 32.23545 10.74515 22.90694 0 0 0 # C_R

395 1 4 -0.1283 31.02297 10.74515 22.19303 0 0 0 # C_R

396 1 1 0.4583 33.35295 11.86308 31.11795 0 0 0 # Zn3+2

397 1 3 -0.4661 32.96999 11.48012 29.26979 0 0 0 # O_2

398 1 4 -0.1414 33.09291 11.60304 26.53794 0 0 0 # C_R

399 1 4 -0.1379 33.09119 11.60003 25.14193 0 0 0 # C_R

400 1 4 -0.128 33.44793 10.74515 22.19303 0 0 0 # C_R

401 1 1 0.4583 33.35295 9.62808 33.35295 0 0 0 # Zn3+2

402 1 3 -0.4664 32.96999 10.01018 35.20111 0 0 0 # O_2

403 1 4 0.5957 32.23545 10.74515 35.86903 0 0 0 # C_R

404 1 4 0.0296 32.23545 10.74515 37.24914 0 0 0 # C_R

405 1 4 -0.1412 33.09291 9.88812 37.93296 0 0 0 # C_R

406 1 4 -0.1383 33.09119 9.88984 39.32897 0 0 0 # C_R

407 1 4 0.0278 32.23545 10.74515 40.03901 0 0 0 # C_R

408 1 4 0.0292 32.23545 10.74515 41.56396 0 0 0 # C_R

409 1 1 0.4583 31.11795 11.86308 33.35295 0 0 0 # Zn3+2

410 1 3 -0.4663 31.50005 11.48012 35.20111 0 0 0 # O_2

411 1 4 -0.1416 31.37799 11.60304 37.93296 0 0 0 # C_R

412 1 4 -0.1381 31.38014 11.60003 39.32897 0 0 0 # C_R

413 1 3 -0.4661 29.26979 10.01018 31.50005 0 0 0 # O_2

414 1 4 0.5954 28.60101 10.74515 32.23545 0 0 0 # C_R

415 1 4 0.0296 27.22219 10.74515 32.23545 0 0 0 # C_R

416 1 4 -0.1412 26.53794 9.88812 31.37799 0 0 0 # C_R

417 1 4 -0.1383 25.14193 9.88984 31.38014 0 0 0 # C_R

418 1 4 0.0276 24.43189 10.74515 32.23545 0 0 0 # C_R

419 1 4 0.0288 22.90694 10.74515 32.23545 0 0 0 # C_R

420 1 3 -0.4662 29.26979 11.48012 32.96999 0 0 0 # O_2

421 1 4 -0.1415 26.53794 11.60304 33.09291 0 0 0 # C_R

422 1 4 -0.1381 25.14193 11.60003 33.09119 0 0 0 # C_R

423 1 3 -0.4664 35.20111 11.48012 31.50005 0 0 0 # O_2

424 1 4 0.5957 35.86903 10.74515 32.23545 0 0 0 # C_R

425 1 4 0.0296 37.24914 10.74515 32.23545 0 0 0 # C_R

426 1 4 -0.1415 37.93296 11.60304 31.37799 0 0 0 # C_R

427 1 4 -0.1384 39.32897 11.60003 31.38014 0 0 0 # C_R

428 1 4 0.0278 40.03901 10.74515 32.23545 0 0 0 # C_R

429 1 4 0.0292 41.56396 10.74515 32.23545 0 0 0 # C_R

430 1 4 -0.1282 42.27787 11.9572 32.23545 0 0 0 # C_R

431 1 3 -0.4663 35.20111 10.01018 32.96999 0 0 0 # O_2

432 1 4 -0.1413 37.93296 9.88812 33.09291 0 0 0 # C_R

433 1 4 -0.1379 39.32897 9.88984 33.09119 0 0 0 # C_R

434 1 4 -0.128 42.27787 9.5331 32.23545 0 0 0 # C_R

435 1 3 -0.4661 31.50005 7.77906 31.50005 0 0 0 # O_2

436 1 4 0.5957 32.23545 7.11114 32.23545 0 0 0 # C_R

437 1 4 0.0296 32.23545 5.73103 32.23545 0 0 0 # C_R

438 1 4 -0.1411 31.37799 5.04807 31.37799 0 0 0 # C_R

439 1 4 -0.1381 31.38014 3.6512 31.38014 0 0 0 # C_R

440 1 4 0.0278 32.23545 2.94202 32.23545 0 0 0 # C_R

441 1 4 0.0293 32.23545 1.41621 32.23545 0 0 0 # C_R

442 1 4 -0.1282 32.23545 0.70316 31.02297 0 0 0 # C_R

443 1 3 -0.4665 32.96999 7.77906 32.96999 0 0 0 # O_2

444 1 4 -0.1417 33.09291 5.04807 33.09291 0 0 0 # C_R

445 1 4 -0.1384 33.09119 3.6512 33.09119 0 0 0 # C_R

446 1 4 -0.1281 32.23545 0.70316 33.44793 0 0 0 # C_R

447 1 3 -0.4663 31.50005 13.71081 32.96999 0 0 0 # O_2

448 1 4 0.5955 32.23545 14.37916 32.23545 0 0 0 # C_R

449 1 4 0.0295 32.23545 15.75884 32.23545 0 0 0 # C_R

450 1 4 -0.1413 31.37799 16.4418 33.09291 0 0 0 # C_R

451 1 4 -0.1383 31.38014 17.8391 33.09119 0 0 0 # C_R

452 1 4 0.0278 32.23545 18.54785 32.23545 0 0 0 # C_R

453 1 4 0.0292 32.23545 20.07409 32.23545 0 0 0 # C_R

454 1 3 -0.4662 32.96999 13.71081 31.50005 0 0 0 # O_2

455 1 4 -0.1416 33.09291 16.4418 31.37799 0 0 0 # C_R

456 1 4 -0.1381 33.09119 17.8391 31.38014 0 0 0 # C_R

457 1 4 -0.1282 32.23545 9.5331 42.27787 0 0 0 # C_R

458 1 4 -0.1279 32.23545 11.9572 42.27787 0 0 0 # C_R

459 1 4 -0.1279 33.44793 20.788 32.23545 0 0 0 # C_R

460 1 4 -0.1278 31.02297 20.788 32.23545 0 0 0 # C_R

461 1 4 -0.128 22.19303 10.74515 33.44793 0 0 0 # C_R

462 1 4 -0.1283 22.19303 10.74515 31.02297 0 0 0 # C_R

463 1 1 0.4585 31.11795 31.11795 9.62808 0 0 0 # Zn3+2

464 1 2 -0.1878 32.23545 32.23545 10.74515 0 0 0 # O_3

465 1 3 -0.4662 31.50005 31.50005 7.77906 0 0 0 # O_2

466 1 4 0.5957 32.23545 32.23545 7.11114 0 0 0 # C_R

467 1 4 0.0296 32.23545 32.23545 5.73103 0 0 0 # C_R

468 1 4 -0.1413 31.37799 31.37799 5.04807 0 0 0 # C_R

469 1 4 -0.1383 31.38014 31.38014 3.6512 0 0 0 # C_R

470 1 4 0.0278 32.23545 32.23545 2.94202 0 0 0 # C_R

471 1 4 0.0293 32.23545 32.23545 1.41621 0 0 0 # C_R

472 1 1 0.4583 33.35295 33.35295 9.62808 0 0 0 # Zn3+2

473 1 3 -0.4664 32.96999 32.96999 7.77906 0 0 0 # O_2

474 1 4 -0.1415 33.09291 33.09291 5.04807 0 0 0 # C_R

475 1 4 -0.1381 33.09119 33.09119 3.6512 0 0 0 # C_R

476 1 1 0.4583 33.35295 31.11795 11.86308 0 0 0 # Zn3+2

477 1 3 -0.4662 32.96999 31.50005 13.71081 0 0 0 # O_2

478 1 4 0.5955 32.23545 32.23545 14.37916 0 0 0 # C_R

479 1 4 0.0295 32.23545 32.23545 15.75884 0 0 0 # C_R

480 1 4 -0.1411 33.09291 31.37799 16.4418 0 0 0 # C_R

481 1 4 -0.1381 33.09119 31.38014 17.8391 0 0 0 # C_R

482 1 4 0.0278 32.23545 32.23545 18.54785 0 0 0 # C_R

483 1 4 0.0292 32.23545 32.23545 20.07409 0 0 0 # C_R

484 1 4 -0.1279 33.44793 32.23545 20.788 0 0 0 # C_R

485 1 1 0.4583 31.11795 33.35295 11.86308 0 0 0 # Zn3+2

486 1 3 -0.4663 31.50005 32.96999 13.71081 0 0 0 # O_2

487 1 4 -0.1418 31.37799 33.09291 16.4418 0 0 0 # C_R

488 1 4 -0.1383 31.38014 33.09119 17.8391 0 0 0 # C_R

489 1 4 -0.1278 31.02297 32.23545 20.788 0 0 0 # C_R

490 1 3 -0.4661 29.26979 31.50005 10.01018 0 0 0 # O_2

491 1 4 0.5954 28.60101 32.23545 10.74515 0 0 0 # C_R

492 1 4 0.0296 27.22219 32.23545 10.74515 0 0 0 # C_R

493 1 4 -0.1412 26.53794 31.37799 9.88812 0 0 0 # C_R

494 1 4 -0.1384 25.14193 31.38014 9.88984 0 0 0 # C_R

495 1 4 0.0276 24.43189 32.23545 10.74515 0 0 0 # C_R

496 1 4 0.0287 22.90694 32.23545 10.74515 0 0 0 # C_R

497 1 3 -0.4662 29.26979 32.96999 11.48012 0 0 0 # O_2

498 1 4 -0.1416 26.53794 33.09291 11.60304 0 0 0 # C_R

499 1 4 -0.1381 25.14193 33.09119 11.60003 0 0 0 # C_R

500 1 3 -0.4663 35.20111 32.96999 10.01018 0 0 0 # O_2

501 1 4 0.5957 35.86903 32.23545 10.74515 0 0 0 # C_R

502 1 4 0.0296 37.24914 32.23545 10.74515 0 0 0 # C_R

503 1 4 -0.141 37.93296 33.09291 9.88812 0 0 0 # C_R

504 1 4 -0.1381 39.32897 33.09119 9.88984 0 0 0 # C_R

505 1 4 0.0278 40.03901 32.23545 10.74515 0 0 0 # C_R

506 1 4 0.0293 41.56396 32.23545 10.74515 0 0 0 # C_R

507 1 4 -0.1281 42.27787 33.44793 10.74515 0 0 0 # C_R

508 1 3 -0.4664 35.20111 31.50005 11.48012 0 0 0 # O_2

509 1 4 -0.1418 37.93296 31.37799 11.60304 0 0 0 # C_R

510 1 4 -0.1383 39.32897 31.38014 11.60003 0 0 0 # C_R

511 1 4 -0.1281 42.27787 31.02297 10.74515 0 0 0 # C_R

512 1 3 -0.4661 31.50005 29.26979 10.01018 0 0 0 # O_2

513 1 4 0.5954 32.23545 28.60101 10.74515 0 0 0 # C_R

514 1 4 0.0296 32.23545 27.22219 10.74515 0 0 0 # C_R

515 1 4 -0.1412 31.37799 26.53794 9.88812 0 0 0 # C_R

516 1 4 -0.1384 31.38014 25.14193 9.88984 0 0 0 # C_R

517 1 4 0.0276 32.23545 24.43189 10.74515 0 0 0 # C_R

518 1 4 0.0288 32.23545 22.90694 10.74515 0 0 0 # C_R

519 1 3 -0.4662 32.96999 29.26979 11.48012 0 0 0 # O_2

520 1 4 -0.1416 33.09291 26.53794 11.60304 0 0 0 # C_R

521 1 4 -0.138 33.09119 25.14193 11.60003 0 0 0 # C_R

522 1 3 -0.4662 31.50005 35.20111 11.48012 0 0 0 # O_2

523 1 4 0.5957 32.23545 35.86903 10.74515 0 0 0 # C_R

524 1 4 0.0296 32.23545 37.24914 10.74515 0 0 0 # C_R

525 1 4 -0.1411 31.37799 37.93296 11.60304 0 0 0 # C_R

526 1 4 -0.1381 31.38014 39.32897 11.60003 0 0 0 # C_R

527 1 4 0.0278 32.23545 40.03901 10.74515 0 0 0 # C_R

528 1 4 0.0292 32.23545 41.56396 10.74515 0 0 0 # C_R

529 1 4 -0.1281 32.23545 42.27787 11.9572 0 0 0 # C_R

530 1 3 -0.4665 32.96999 35.20111 10.01018 0 0 0 # O_2

531 1 4 -0.1417 33.09291 37.93296 9.88812 0 0 0 # C_R

532 1 4 -0.1383 33.09119 39.32897 9.88984 0 0 0 # C_R

533 1 4 -0.1281 32.23545 42.27787 9.5331 0 0 0 # C_R

534 1 4 -0.1282 32.23545 33.44793 0.70316 0 0 0 # C_R

535 1 4 -0.1281 32.23545 31.02297 0.70316 0 0 0 # C_R

536 1 4 -0.1281 31.02297 22.19303 10.74515 0 0 0 # C_R

537 1 4 -0.1282 33.44793 22.19303 10.74515 0 0 0 # C_R

538 1 4 -0.1282 22.19303 32.23545 11.9572 0 0 0 # C_R

539 1 4 -0.1281 22.19303 32.23545 9.5331 0 0 0 # C_R

540 1 2 -0.1878 32.23545 32.23545 32.23545 0 0 0 # O_3

541 1 4 0.5954 32.23545 32.23545 28.60101 0 0 0 # C_R

542 1 4 0.0297 32.23545 32.23545 27.22219 0 0 0 # C_R

543 1 4 0.0279 32.23545 32.23545 24.43189 0 0 0 # C_R

544 1 4 0.0289 32.23545 32.23545 22.90694 0 0 0 # C_R

545 1 4 -0.1282 31.02297 32.23545 22.19303 0 0 0 # C_R

546 1 4 -0.1281 33.44793 32.23545 22.19303 0 0 0 # C_R

547 1 4 0.5957 32.23545 32.23545 35.86903 0 0 0 # C_R

548 1 4 0.0297 32.23545 32.23545 37.24914 0 0 0 # C_R

549 1 4 0.0281 32.23545 32.23545 40.03901 0 0 0 # C_R

550 1 4 0.0294 32.23545 32.23545 41.56396 0 0 0 # C_R

551 1 4 0.5954 28.60101 32.23545 32.23545 0 0 0 # C_R

552 1 4 0.0297 27.22219 32.23545 32.23545 0 0 0 # C_R

553 1 4 0.0279 24.43189 32.23545 32.23545 0 0 0 # C_R

554 1 4 0.0289 22.90694 32.23545 32.23545 0 0 0 # C_R

555 1 4 0.5957 35.86903 32.23545 32.23545 0 0 0 # C_R

556 1 4 0.0296 37.24914 32.23545 32.23545 0 0 0 # C_R

557 1 4 0.0281 40.03901 32.23545 32.23545 0 0 0 # C_R

558 1 4 0.0294 41.56396 32.23545 32.23545 0 0 0 # C_R

559 1 4 -0.128 42.27787 33.44793 32.23545 0 0 0 # C_R

560 1 4 -0.1281 42.27787 31.02297 32.23545 0 0 0 # C_R

561 1 4 0.5954 32.23545 28.60101 32.23545 0 0 0 # C_R

562 1 4 0.0297 32.23545 27.22219 32.23545 0 0 0 # C_R

563 1 4 0.0279 32.23545 24.43189 32.23545 0 0 0 # C_R

564 1 4 0.0289 32.23545 22.90694 32.23545 0 0 0 # C_R

565 1 4 0.5957 32.23545 35.86903 32.23545 0 0 0 # C_R

566 1 4 0.0296 32.23545 37.24914 32.23545 0 0 0 # C_R

567 1 4 0.0281 32.23545 40.03901 32.23545 0 0 0 # C_R

568 1 4 0.0294 32.23545 41.56396 32.23545 0 0 0 # C_R

569 1 4 -0.1281 32.23545 42.27787 33.44793 0 0 0 # C_R

570 1 4 -0.128 32.23545 42.27787 31.02297 0 0 0 # C_R

571 1 1 0.4584 31.11795 31.11795 33.35295 0 0 0 # Zn3+2

572 1 3 -0.4662 31.50005 31.50005 35.20111 0 0 0 # O_2

573 1 4 -0.1416 31.37799 31.37799 37.93296 0 0 0 # C_R

574 1 4 -0.1383 31.38014 31.38014 39.32897 0 0 0 # C_R

575 1 4 -0.1279 32.23545 31.02297 42.27787 0 0 0 # C_R

576 1 1 0.4582 33.35295 33.35295 33.35295 0 0 0 # Zn3+2

577 1 3 -0.4664 32.96999 32.96999 35.20111 0 0 0 # O_2

578 1 4 -0.1416 33.09291 33.09291 37.93296 0 0 0 # C_R

579 1 4 -0.1381 33.09119 33.09119 39.32897 0 0 0 # C_R

580 1 4 -0.1281 32.23545 33.44793 42.27787 0 0 0 # C_R

581 1 1 0.4584 31.11795 33.35295 31.11795 0 0 0 # Zn3+2

582 1 3 -0.4662 31.50005 32.96999 29.26979 0 0 0 # O_2

583 1 4 -0.1417 31.37799 33.09291 26.53794 0 0 0 # C_R

584 1 4 -0.1384 31.38014 33.09119 25.14193 0 0 0 # C_R

585 1 1 0.4584 33.35295 31.11795 31.11795 0 0 0 # Zn3+2

586 1 3 -0.466 32.96999 31.50005 29.26979 0 0 0 # O_2

587 1 4 -0.1413 33.09291 31.37799 26.53794 0 0 0 # C_R

588 1 4 -0.1381 33.09119 31.38014 25.14193 0 0 0 # C_R

589 1 3 -0.4661 31.50005 29.26979 32.96999 0 0 0 # O_2

590 1 4 -0.1415 31.37799 26.53794 33.09291 0 0 0 # C_R

591 1 4 -0.1383 31.38014 25.14193 33.09119 0 0 0 # C_R

592 1 4 -0.128 31.02297 22.19303 32.23545 0 0 0 # C_R

593 1 3 -0.4661 32.96999 29.26979 31.50005 0 0 0 # O_2

594 1 4 -0.1415 33.09291 26.53794 31.37799 0 0 0 # C_R

595 1 4 -0.1382 33.09119 25.14193 31.38014 0 0 0 # C_R

596 1 4 -0.1282 33.44793 22.19303 32.23545 0 0 0 # C_R

597 1 3 -0.4665 32.96999 35.20111 32.96999 0 0 0 # O_2

598 1 4 -0.1417 33.09291 37.93296 33.09291 0 0 0 # C_R

599 1 4 -0.1383 33.09119 39.32897 33.09119 0 0 0 # C_R

600 1 3 -0.4662 31.50005 35.20111 31.50005 0 0 0 # O_2

601 1 4 -0.1414 31.37799 37.93296 31.37799 0 0 0 # C_R

602 1 4 -0.138 31.38014 39.32897 31.38014 0 0 0 # C_R

603 1 3 -0.4661 29.26979 31.50005 32.96999 0 0 0 # O_2

604 1 4 -0.1415 26.53794 31.37799 33.09291 0 0 0 # C_R

605 1 4 -0.1383 25.14193 31.38014 33.09119 0 0 0 # C_R

606 1 4 -0.128 22.19303 32.23545 33.44793 0 0 0 # C_R

607 1 3 -0.4661 29.26979 32.96999 31.50005 0 0 0 # O_2

608 1 4 -0.1415 26.53794 33.09291 31.37799 0 0 0 # C_R

609 1 4 -0.1382 25.14193 33.09119 31.38014 0 0 0 # C_R

610 1 4 -0.1282 22.19303 32.23545 31.02297 0 0 0 # C_R

611 1 3 -0.4663 35.20111 31.50005 31.50005 0 0 0 # O_2

612 1 4 -0.1418 37.93296 31.37799 31.37799 0 0 0 # C_R

613 1 4 -0.1384 39.32897 31.38014 31.38014 0 0 0 # C_R

614 1 3 -0.4663 35.20111 32.96999 32.96999 0 0 0 # O_2

615 1 4 -0.1414 37.93296 33.09291 33.09291 0 0 0 # C_R

616 1 4 -0.138 39.32897 33.09119 33.09119 0 0 0 # C_R

617 1 5 0.1507 9.3096 9.3096 5.5316 0 0 0 # H_

618 1 5 0.1244 9.30874 9.30874 3.17455 0 0 0 # H_

619 1 5 0.1506 12.18113 12.18113 5.53203 0 0 0 # H_

620 1 5 0.1243 12.18414 12.18414 3.18142 0 0 0 # H_

621 1 5 0.1506 12.18199 9.31046 15.95827 0 0 0 # H_

622 1 5 0.1243 12.1807 9.30874 18.31618 0 0 0 # H_

623 1 5 0.1206 12.78415 10.74515 20.31994 0 0 0 # H_

624 1 5 0.1507 9.31046 12.18199 15.95827 0 0 0 # H_

625 1 5 0.1245 9.3053 12.18371 18.30845 0 0 0 # H_

626 1 5 0.1209 8.70615 10.74515 20.32037 0 0 0 # H_

627 1 5 0.1508 5.5316 9.3096 9.3096 0 0 0 # H_

628 1 5 0.1246 3.17455 9.30874 9.30874 0 0 0 # H_

629 1 5 0.1208 1.17122 8.70658 10.74515 0 0 0 # H_

630 1 5 0.1505 5.53203 12.18113 12.18113 0 0 0 # H_

631 1 5 0.1241 3.18142 12.18414 12.18414 0 0 0 # H_

632 1 5 0.1206 1.17122 12.78372 10.74515 0 0 0 # H_

633 1 5 0.1507 15.95827 12.18199 9.31046 0 0 0 # H_

634 1 5 0.1244 18.31618 12.1807 9.30874 0 0 0 # H_

635 1 5 0.1506 15.95827 9.31046 12.18199 0 0 0 # H_

636 1 5 0.1244 18.30845 9.3053 12.18371 0 0 0 # H_

637 1 5 0.1505 9.3096 5.5316 9.3096 0 0 0 # H_

638 1 5 0.1242 9.30874 3.17455 9.30874 0 0 0 # H_

639 1 5 0.1206 10.74515 1.17122 8.70658 0 0 0 # H_

640 1 5 0.1508 12.18113 5.53203 12.18113 0 0 0 # H_

641 1 5 0.1245 12.18414 3.18142 12.18414 0 0 0 # H_

642 1 5 0.1209 10.74515 1.17122 12.78372 0 0 0 # H_

643 1 5 0.1507 9.31046 15.95827 12.18199 0 0 0 # H_

644 1 5 0.1245 9.30874 18.31618 12.1807 0 0 0 # H_

645 1 5 0.1506 12.18199 15.95827 9.31046 0 0 0 # H_

646 1 5 0.1243 12.18371 18.30845 9.3053 0 0 0 # H_

647 1 5 0.1209 10.74515 12.78372 1.17122 0 0 0 # H_

648 1 5 0.1205 10.74515 8.70658 1.17122 0 0 0 # H_

649 1 5 0.121 12.78415 20.31994 10.74515 0 0 0 # H_

650 1 5 0.1206 8.70615 20.32037 10.74515 0 0 0 # H_

651 1 5 0.1209 20.32037 10.74515 8.70615 0 0 0 # H_

652 1 5 0.1206 20.31994 10.74515 12.78415 0 0 0 # H_

653 1 5 0.121 8.71045 10.74515 22.66797 0 0 0 # H_

654 1 5 0.1207 12.77985 10.74515 22.66797 0 0 0 # H_

655 1 5 0.1209 1.17122 8.70658 32.23545 0 0 0 # H_

656 1 5 0.1206 1.17122 12.78372 32.23545 0 0 0 # H_

657 1 5 0.1209 10.74515 1.17122 30.19645 0 0 0 # H_

658 1 5 0.1206 10.74515 1.17165 34.27445 0 0 0 # H_

659 1 5 0.1506 9.3096 9.3096 37.44986 0 0 0 # H_

660 1 5 0.1243 9.30573 9.30573 39.79789 0 0 0 # H_

661 1 5 0.1206 10.74515 8.70615 41.81024 0 0 0 # H_

662 1 5 0.1506 12.18113 12.18113 37.44943 0 0 0 # H_

663 1 5 0.1243 12.18414 12.18414 39.79789 0 0 0 # H_

664 1 5 0.1209 10.74515 12.78415 41.80981 0 0 0 # H_

665 1 5 0.1507 9.31046 12.18199 27.02104 0 0 0 # H_

666 1 5 0.1244 9.30573 12.18414 24.67301 0 0 0 # H_

667 1 5 0.1505 12.18242 9.31089 27.02147 0 0 0 # H_

668 1 5 0.1241 12.18414 9.30573 24.67301 0 0 0 # H_

669 1 5 0.1505 9.31046 5.5316 33.67143 0 0 0 # H_

670 1 5 0.1242 9.30659 3.18142 33.6753 0 0 0 # H_

671 1 5 0.1507 12.18199 5.5316 30.80033 0 0 0 # H_

672 1 5 0.1245 12.18371 3.18185 30.7956 0 0 0 # H_

673 1 5 0.1506 12.18156 15.95784 33.67057 0 0 0 # H_

674 1 5 0.1243 12.18328 18.30888 33.67573 0 0 0 # H_

675 1 5 0.121 12.78415 20.31994 32.23545 0 0 0 # H_

676 1 5 0.1506 9.3096 15.95827 30.7999 0 0 0 # H_

677 1 5 0.1243 9.30573 18.30845 30.79603 0 0 0 # H_

678 1 5 0.1206 8.70615 20.32037 32.23545 0 0 0 # H_

679 1 5 0.1508 5.5316 9.31046 33.67143 0 0 0 # H_

680 1 5 0.1245 3.18142 9.30659 33.6753 0 0 0 # H_

681 1 5 0.1505 5.5316 12.18199 30.80033 0 0 0 # H_

682 1 5 0.1241 3.18185 12.18371 30.7956 0 0 0 # H_

683 1 5 0.1506 15.95827 9.3096 30.7999 0 0 0 # H_

684 1 5 0.1243 18.30845 9.30573 30.79603 0 0 0 # H_

685 1 5 0.1206 20.31994 10.74515 30.19602 0 0 0 # H_

686 1 5 0.1506 15.95784 12.18156 33.67057 0 0 0 # H_

687 1 5 0.1243 18.30888 12.18328 33.67573 0 0 0 # H_

688 1 5 0.121 20.31994 10.74515 34.27445 0 0 0 # H_

689 1 5 0.1207 12.78415 32.23545 20.31994 0 0 0 # H_

690 1 5 0.1209 8.70615 32.23545 20.32037 0 0 0 # H_

691 1 5 0.1206 1.17122 30.19645 10.74515 0 0 0 # H_

692 1 5 0.1209 1.17165 34.27445 10.74515 0 0 0 # H_

693 1 5 0.1208 10.74515 41.80981 12.78415 0 0 0 # H_

694 1 5 0.1206 10.74515 41.81024 8.70615 0 0 0 # H_

695 1 5 0.1507 9.3096 30.7999 15.95827 0 0 0 # H_

696 1 5 0.1245 9.30573 30.79603 18.30845 0 0 0 # H_

697 1 5 0.1505 12.18156 33.67057 15.95784 0 0 0 # H_

698 1 5 0.1241 12.18328 33.67573 18.30888 0 0 0 # H_

699 1 5 0.1506 9.31046 33.67143 5.5316 0 0 0 # H_

700 1 5 0.1244 9.30659 33.6753 3.18142 0 0 0 # H_

701 1 5 0.1205 10.74515 34.27445 1.17165 0 0 0 # H_

702 1 5 0.1506 12.18199 30.80033 5.5316 0 0 0 # H_

703 1 5 0.1242 12.18371 30.7956 3.18185 0 0 0 # H_

704 1 5 0.1209 10.74515 30.19645 1.17122 0 0 0 # H_

705 1 5 0.1506 9.31046 27.02104 12.18199 0 0 0 # H_

706 1 5 0.1243 9.3053 24.67301 12.18371 0 0 0 # H_

707 1 5 0.1207 8.71045 22.66797 10.74515 0 0 0 # H_

708 1 5 0.1506 12.18199 27.02104 9.31046 0 0 0 # H_

709 1 5 0.1242 12.18371 24.67301 9.3053 0 0 0 # H_

710 1 5 0.121 12.77985 22.66797 10.74515 0 0 0 # H_

711 1 5 0.1507 12.18113 37.44943 12.18113 0 0 0 # H_

712 1 5 0.1245 12.18414 39.79789 12.18414 0 0 0 # H_

713 1 5 0.1504 9.3096 37.44986 9.3096 0 0 0 # H_

714 1 5 0.1241 9.30573 39.79789 9.30573 0 0 0 # H_

715 1 5 0.1505 5.5316 30.80033 12.18199 0 0 0 # H_

716 1 5 0.1241 3.18185 30.7956 12.18371 0 0 0 # H_

717 1 5 0.1508 5.5316 33.67143 9.31046 0 0 0 # H_

718 1 5 0.1245 3.18142 33.6753 9.30659 0 0 0 # H_

719 1 5 0.1506 15.95827 30.7999 9.3096 0 0 0 # H_

720 1 5 0.1243 18.30845 30.79603 9.30573 0 0 0 # H_

721 1 5 0.121 20.32037 32.23545 8.70615 0 0 0 # H_

722 1 5 0.1506 15.95784 33.67057 12.18156 0 0 0 # H_

723 1 5 0.1244 18.30888 33.67573 12.18328 0 0 0 # H_

724 1 5 0.1206 20.31994 32.23545 12.78415 0 0 0 # H_

725 1 5 0.1508 9.3096 30.79947 27.02104 0 0 0 # H_

726 1 5 0.1245 9.30874 30.79904 24.66528 0 0 0 # H_

727 1 5 0.1209 8.71045 32.23545 22.66797 0 0 0 # H_

728 1 5 0.1505 12.18156 33.671 27.02147 0 0 0 # H_

729 1 5 0.1241 12.18371 33.67573 24.67258 0 0 0 # H_

730 1 5 0.1208 12.77985 32.23545 22.66797 0 0 0 # H_

731 1 5 0.1507 12.18199 30.80033 37.44986 0 0 0 # H_

732 1 5 0.1244 12.1807 30.79904 39.80605 0 0 0 # H_

733 1 5 0.1505 9.31003 33.67186 37.44986 0 0 0 # H_

734 1 5 0.1243 9.30573 33.6753 39.79832 0 0 0 # H_

735 1 5 0.1506 5.5316 30.7999 30.7999 0 0 0 # H_

736 1 5 0.1242 3.17455 30.79904 30.79904 0 0 0 # H_

737 1 5 0.1206 1.17122 30.19645 32.23545 0 0 0 # H_

738 1 5 0.1508 5.53203 33.671 33.671 0 0 0 # H_

739 1 5 0.1245 3.18142 33.67487 33.67487 0 0 0 # H_

740 1 5 0.1209 1.17165 34.27445 32.23545 0 0 0 # H_

741 1 5 0.1507 15.95827 33.67143 30.80033 0 0 0 # H_

742 1 5 0.1244 18.31575 33.67229 30.79904 0 0 0 # H_

743 1 5 0.1506 15.95827 30.80033 33.67143 0 0 0 # H_

744 1 5 0.1243 18.30845 30.79646 33.6753 0 0 0 # H_

745 1 5 0.1506 9.3096 27.02104 30.79947 0 0 0 # H_

746 1 5 0.1244 9.30874 24.66528 30.79904 0 0 0 # H_

747 1 5 0.1506 12.18156 27.02147 33.671 0 0 0 # H_

748 1 5 0.1243 12.18371 24.67258 33.67573 0 0 0 # H_

749 1 5 0.1505 9.31003 37.44986 33.67186 0 0 0 # H_

750 1 5 0.1242 9.30917 39.80562 33.67272 0 0 0 # H_

751 1 5 0.1205 10.74515 41.80981 34.27488 0 0 0 # H_

752 1 5 0.1507 12.18199 37.44986 30.80033 0 0 0 # H_

753 1 5 0.1245 12.18371 39.79789 30.7956 0 0 0 # H_

754 1 5 0.1209 10.74515 41.80981 30.19602 0 0 0 # H_

755 1 5 0.1208 10.74515 30.19602 41.80981 0 0 0 # H_

756 1 5 0.1205 10.74515 34.27488 41.80981 0 0 0 # H_

757 1 5 0.1206 8.71045 22.66797 32.23545 0 0 0 # H_

758 1 5 0.1211 12.77985 22.66797 32.23545 0 0 0 # H_

759 1 5 0.1206 20.31994 32.23545 30.19602 0 0 0 # H_

760 1 5 0.121 20.31994 32.23545 34.27445 0 0 0 # H_

761 1 5 0.1209 34.27445 10.74515 20.31994 0 0 0 # H_

762 1 5 0.1207 30.19602 10.74515 20.31994 0 0 0 # H_

763 1 5 0.1206 41.80981 12.78415 10.74515 0 0 0 # H_

764 1 5 0.1209 41.81024 8.70615 10.74515 0 0 0 # H_

765 1 5 0.1206 32.23545 1.17122 8.70658 0 0 0 # H_

766 1 5 0.1209 32.23545 1.17122 12.78372 0 0 0 # H_

767 1 5 0.1505 30.7999 9.3096 15.95827 0 0 0 # H_

768 1 5 0.1241 30.79603 9.30573 18.30845 0 0 0 # H_

769 1 5 0.1507 33.67057 12.18156 15.95784 0 0 0 # H_

770 1 5 0.1245 33.67573 12.18328 18.30888 0 0 0 # H_

771 1 5 0.1506 30.80033 12.18199 5.5316 0 0 0 # H_

772 1 5 0.1243 30.7956 12.18371 3.18185 0 0 0 # H_

773 1 5 0.1209 32.23545 12.78372 1.17122 0 0 0 # H_

774 1 5 0.1506 33.67143 9.31046 5.5316 0 0 0 # H_

775 1 5 0.1244 33.6753 9.30659 3.18142 0 0 0 # H_

776 1 5 0.1206 32.23545 8.70658 1.17122 0 0 0 # H_

777 1 5 0.1507 30.80033 5.5316 12.18199 0 0 0 # H_

778 1 5 0.1245 30.7956 3.18185 12.18371 0 0 0 # H_

779 1 5 0.1505 33.67143 5.5316 9.31046 0 0 0 # H_

780 1 5 0.1242 33.6753 3.18142 9.30659 0 0 0 # H_

781 1 5 0.1506 33.67057 15.95784 12.18156 0 0 0 # H_

782 1 5 0.1243 33.67573 18.30888 12.18328 0 0 0 # H_

783 1 5 0.1206 34.27445 20.31994 10.74515 0 0 0 # H_

784 1 5 0.1506 30.7999 15.95827 9.3096 0 0 0 # H_

785 1 5 0.1243 30.79603 18.30845 9.30573 0 0 0 # H_

786 1 5 0.121 30.19602 20.31994 10.74515 0 0 0 # H_

787 1 5 0.1506 27.02104 9.31046 12.18199 0 0 0 # H_

788 1 5 0.1243 24.67301 9.3053 12.18371 0 0 0 # H_

789 1 5 0.1207 22.66797 10.74515 12.77985 0 0 0 # H_

790 1 5 0.1506 27.02104 12.18199 9.31046 0 0 0 # H_

791 1 5 0.1242 24.67301 12.18371 9.3053 0 0 0 # H_

792 1 5 0.121 22.66797 10.74515 8.71045 0 0 0 # H_

793 1 5 0.1507 37.44986 9.3096 9.3096 0 0 0 # H_

794 1 5 0.1245 39.79789 9.30573 9.30573 0 0 0 # H_

795 1 5 0.1505 37.44943 12.18113 12.18113 0 0 0 # H_

796 1 5 0.1241 39.79789 12.18414 12.18414 0 0 0 # H_

797 1 5 0.1505 30.79947 9.3096 27.02104 0 0 0 # H_

798 1 5 0.1242 30.79904 9.30874 24.66528 0 0 0 # H_

799 1 5 0.1207 30.20032 10.74515 22.66797 0 0 0 # H_

800 1 5 0.1507 33.671 12.18156 27.02147 0 0 0 # H_

801 1 5 0.1244 33.67573 12.18371 24.67258 0 0 0 # H_

802 1 5 0.121 34.27058 10.74515 22.66797 0 0 0 # H_

803 1 5 0.1506 33.67186 9.31003 37.44986 0 0 0 # H_

804 1 5 0.1244 33.67272 9.30917 39.80562 0 0 0 # H_

805 1 5 0.1506 30.80033 12.18199 37.44986 0 0 0 # H_

806 1 5 0.1243 30.7956 12.18371 39.79789 0 0 0 # H_

807 1 5 0.1506 27.02104 9.3096 30.79947 0 0 0 # H_

808 1 5 0.1244 24.66528 9.30874 30.79904 0 0 0 # H_

809 1 5 0.1506 27.02147 12.18156 33.671 0 0 0 # H_

810 1 5 0.1242 24.67258 12.18371 33.67573 0 0 0 # H_

811 1 5 0.1505 37.44986 12.18199 30.80033 0 0 0 # H_

812 1 5 0.1243 39.80605 12.1807 30.79904 0 0 0 # H_

813 1 5 0.1205 41.80981 12.78415 32.23545 0 0 0 # H_

814 1 5 0.1507 37.44986 9.31003 33.67186 0 0 0 # H_

815 1 5 0.1245 39.79832 9.30573 33.6753 0 0 0 # H_

816 1 5 0.1209 41.81024 8.70615 32.23545 0 0 0 # H_

817 1 5 0.1509 30.7999 5.5316 30.7999 0 0 0 # H_

818 1 5 0.1246 30.79904 3.17455 30.79904 0 0 0 # H_

819 1 5 0.1208 32.23545 1.17122 30.19645 0 0 0 # H_

820 1 5 0.1505 33.671 5.53203 33.671 0 0 0 # H_

821 1 5 0.1242 33.67487 3.18142 33.67487 0 0 0 # H_

822 1 5 0.1206 32.23545 1.17165 34.27445 0 0 0 # H_

823 1 5 0.1507 30.80033 15.95827 33.67143 0 0 0 # H_

824 1 5 0.1244 30.79904 18.31575 33.67229 0 0 0 # H_

825 1 5 0.1506 33.67143 15.95827 30.80033 0 0 0 # H_

826 1 5 0.1244 33.6753 18.30845 30.79646 0 0 0 # H_

827 1 5 0.1205 32.23545 8.70615 41.81024 0 0 0 # H_

828 1 5 0.1209 32.23545 12.78415 41.80981 0 0 0 # H_

829 1 5 0.1207 34.27445 20.31994 32.23545 0 0 0 # H_

830 1 5 0.1209 30.19602 20.31994 32.23545 0 0 0 # H_

831 1 5 0.1211 22.66797 10.74515 34.27058 0 0 0 # H_

832 1 5 0.1206 22.66797 10.74515 30.20032 0 0 0 # H_

833 1 5 0.1507 30.7999 30.7999 5.5316 0 0 0 # H_

834 1 5 0.1244 30.79904 30.79904 3.17455 0 0 0 # H_

835 1 5 0.1506 33.671 33.671 5.53203 0 0 0 # H_

836 1 5 0.1244 33.67487 33.67487 3.18142 0 0 0 # H_

837 1 5 0.1508 33.67143 30.80033 15.95827 0 0 0 # H_

838 1 5 0.1246 33.67229 30.79904 18.31575 0 0 0 # H_

839 1 5 0.1209 34.27445 32.23545 20.31994 0 0 0 # H_

840 1 5 0.1505 30.80033 33.67143 15.95827 0 0 0 # H_

841 1 5 0.1242 30.79646 33.6753 18.30845 0 0 0 # H_

842 1 5 0.1207 30.19602 32.23545 20.31994 0 0 0 # H_

843 1 5 0.1506 27.02104 30.79947 9.3096 0 0 0 # H_

844 1 5 0.1243 24.66528 30.79904 9.30874 0 0 0 # H_

845 1 5 0.1506 27.02147 33.671 12.18156 0 0 0 # H_

846 1 5 0.1243 24.67258 33.67573 12.18371 0 0 0 # H_

847 1 5 0.1507 37.44986 33.67186 9.31003 0 0 0 # H_

848 1 5 0.1246 39.80562 33.67272 9.30917 0 0 0 # H_

849 1 5 0.1208 41.80981 34.27488 10.74515 0 0 0 # H_

850 1 5 0.1505 37.44986 30.80033 12.18199 0 0 0 # H_

851 1 5 0.1242 39.79789 30.7956 12.18371 0 0 0 # H_

852 1 5 0.1206 41.80981 30.19602 10.74515 0 0 0 # H_

853 1 5 0.1506 30.79947 27.02104 9.3096 0 0 0 # H_

854 1 5 0.1243 30.79904 24.66528 9.30874 0 0 0 # H_

855 1 5 0.1506 33.671 27.02147 12.18156 0 0 0 # H_

856 1 5 0.1243 33.67573 24.67258 12.18371 0 0 0 # H_

857 1 5 0.1508 30.80033 37.44986 12.18199 0 0 0 # H_

858 1 5 0.1246 30.79904 39.80605 12.1807 0 0 0 # H_

859 1 5 0.1208 32.23545 41.80981 12.78415 0 0 0 # H_

860 1 5 0.1504 33.67186 37.44986 9.31003 0 0 0 # H_

861 1 5 0.1241 33.6753 39.79832 9.30573 0 0 0 # H_

862 1 5 0.1206 32.23545 41.81024 8.70615 0 0 0 # H_

863 1 5 0.1206 32.23545 34.27445 1.17165 0 0 0 # H_

864 1 5 0.1209 32.23545 30.19645 1.17122 0 0 0 # H_

865 1 5 0.121 30.20032 22.66797 10.74515 0 0 0 # H_

866 1 5 0.1207 34.27058 22.66797 10.74515 0 0 0 # H_

867 1 5 0.1207 22.66797 32.23545 12.77985 0 0 0 # H_

868 1 5 0.121 22.66797 32.23545 8.71045 0 0 0 # H_

869 1 5 0.1207 30.20032 32.23545 22.66797 0 0 0 # H_

870 1 5 0.121 34.27058 32.23545 22.66797 0 0 0 # H_

871 1 5 0.1208 41.80981 34.27488 32.23545 0 0 0 # H_

872 1 5 0.1206 41.80981 30.19602 32.23545 0 0 0 # H_

873 1 5 0.1206 32.23545 41.80981 34.27488 0 0 0 # H_

874 1 5 0.1209 32.23545 41.80981 30.19602 0 0 0 # H_

875 1 5 0.1506 30.79947 30.79947 37.44986 0 0 0 # H_

876 1 5 0.1243 30.79603 30.79603 39.79789 0 0 0 # H_

877 1 5 0.1209 32.23545 30.19602 41.80981 0 0 0 # H_

878 1 5 0.1506 33.671 33.671 37.44943 0 0 0 # H_

879 1 5 0.1243 33.6753 33.6753 39.79875 0 0 0 # H_

880 1 5 0.1205 32.23545 34.27488 41.80981 0 0 0 # H_

881 1 5 0.1504 30.7999 33.67186 27.02104 0 0 0 # H_

882 1 5 0.1241 30.79603 33.6753 24.67258 0 0 0 # H_

883 1 5 0.1507 33.67186 30.7999 27.02104 0 0 0 # H_

884 1 5 0.1244 33.6753 30.79603 24.67258 0 0 0 # H_

885 1 5 0.1506 30.7999 27.02104 33.67186 0 0 0 # H_

886 1 5 0.1242 30.79603 24.67258 33.6753 0 0 0 # H_

887 1 5 0.1211 30.20032 22.66797 32.23545 0 0 0 # H_

888 1 5 0.1506 33.67186 27.02104 30.7999 0 0 0 # H_

889 1 5 0.1243 33.6753 24.67258 30.79603 0 0 0 # H_

890 1 5 0.1207 34.27058 22.66797 32.23545 0 0 0 # H_

891 1 5 0.1505 33.671 37.44943 33.671 0 0 0 # H_

892 1 5 0.1241 33.6753 39.79875 33.6753 0 0 0 # H_

893 1 5 0.1507 30.79947 37.44986 30.79947 0 0 0 # H_

894 1 5 0.1245 30.79603 39.79789 30.79603 0 0 0 # H_

895 1 5 0.1506 27.02104 30.7999 33.67186 0 0 0 # H_

896 1 5 0.1242 24.67258 30.79603 33.6753 0 0 0 # H_

897 1 5 0.121 22.66797 32.23545 34.27058 0 0 0 # H_

898 1 5 0.1506 27.02104 33.67186 30.7999 0 0 0 # H_

899 1 5 0.1243 24.67258 33.6753 30.79603 0 0 0 # H_

900 1 5 0.1207 22.66797 32.23545 30.20032 0 0 0 # H_

901 1 5 0.1505 37.44986 30.79947 30.79947 0 0 0 # H_

902 1 5 0.1241 39.79789 30.79603 30.79603 0 0 0 # H_

903 1 5 0.1507 37.44943 33.671 33.671 0 0 0 # H_

904 1 5 0.1245 39.79875 33.6753 33.6753 0 0 0 # H_

**3.#data file for MOF-200**

1288 atoms

5 atom types

-13.005499871 39.016500129 xlo xhi

0.000001014 45.052374570 ylo yhi

-0.003644087 42.312355913 zlo zhi

-26.011000000 0.000000000 0.000000000 xy xz yz

Atoms # full (The fourth column is charge)

1 1 1 0.4538 1.5404 16.0705 10.91702 0 0 0 # Zn3+2

2 1 1 0.439 -0.31733 13.83252 12.1471 0 0 0 # Zn3+2

3 1 2 -0.4846 0.74553 14.61589 13.6029 0 0 0 # O_2

4 1 2 -0.4778 2.0718 16.0832 12.81959 0 0 0 # O_2

5 1 2 -0.4782 11.99781 10.5368 30.96638 0 0 0 # O_2

6 1 2 -0.4833 11.19722 8.93749 29.62158 0 0 0 # O_2

7 1 2 -0.4788 0.08839 27.3558 29.8159 0 0 0 # O_2

8 1 2 -0.4955 2.10788 28.14692 30.0359 0 0 0 # O_2

9 1 2 -0.4881 1.0966 17.95882 10.51172 0 0 0 # O_2

10 1 2 -0.4895 -0.24271 11.97812 11.66229 0 0 0 # O_2

11 1 3 0.0043 4.61961 17.61638 23.15019 0 0 0 # C_R

12 1 3 -0.1147 5.42413 16.71668 23.83961 0 0 0 # C_R

13 1 4 0.1053 5.71719 15.93948 23.42191 0 0 0 # H_

14 1 3 0.007 5.79262 16.97929 25.15352 0 0 0 # C_R

15 1 3 -0.1135 5.35671 18.14169 25.77848 0 0 0 # C_R

16 1 4 0.1097 5.60282 18.3183 26.65908 0 0 0 # H_

17 1 3 0.0027 4.55219 19.04139 25.0892 0 0 0 # C_R

18 1 3 -0.118 4.18358 18.77869 23.7752 0 0 0 # C_R

19 1 4 0.1125 3.64672 19.38149 23.31188 0 0 0 # H_

20 1 3 0.0308 3.3273 16.7243 19.1306 0 0 0 # C_R

21 1 3 -0.1297 3.51671 15.68719 20.03709 0 0 0 # C_R

22 1 4 0.1208 3.36837 14.80872 19.77431 0 0 0 # H_

23 1 3 -0.1291 3.92428 15.96611 21.33568 0 0 0 # C_R

24 1 4 0.1178 4.05249 15.2728 21.9408 0 0 0 # H_

25 1 3 0.0297 4.14303 17.28119 21.72931 0 0 0 # C_R

26 1 3 -0.1294 3.95372 18.31739 20.82328 0 0 0 # C_R

27 1 4 0.1198 4.10188 19.19682 21.08611 0 0 0 # H_

28 1 3 -0.1289 3.54608 18.03942 19.5238 0 0 0 # C_R

29 1 4 0.1224 3.41777 18.73282 18.91948 0 0 0 # H_

30 1 3 0.04 2.04551 15.81248 15.1047 0 0 0 # C_R

31 1 3 -0.1384 1.55098 15.10471 16.19391 0 0 0 # C_R

32 1 4 0.1534 0.92079 14.43478 16.0632 0 0 0 # H_

33 1 3 -0.141 1.99741 15.4021 17.47651 0 0 0 # C_R

34 1 4 0.1247 1.66468 14.9304 18.2043 0 0 0 # H_

35 1 3 0.0284 2.93771 16.40722 17.66951 0 0 0 # C_R

36 1 3 -0.1418 3.43218 17.1149 16.5803 0 0 0 # C_R

37 1 4 0.1259 4.06289 17.78672 16.71059 0 0 0 # H_

38 1 3 -0.135 2.98632 16.8176 15.2977 0 0 0 # C_R

39 1 4 0.1562 3.319 17.2911 14.5694 0 0 0 # H_

40 1 3 0.6177 1.41011 15.59668 13.74419 0 0 0 # C_R

41 1 3 0.0327 6.64971 16.05892 25.9249 0 0 0 # C_R

42 1 3 -0.1237 6.74098 14.76911 25.41499 0 0 0 # C_R

43 1 4 0.1101 6.30507 14.55192 24.62368 0 0 0 # H_

44 1 3 -0.1229 7.48391 13.8068 26.0887 0 0 0 # C_R

45 1 4 0.1154 7.54582 12.9435 25.74929 0 0 0 # H_

46 1 3 0.0349 8.13523 14.13379 27.2722 0 0 0 # C_R

47 1 3 -0.1241 8.0439 15.42368 27.7821 0 0 0 # C_R

48 1 4 0.1075 8.47959 15.64218 28.57599 0 0 0 # H_

49 1 3 -0.1254 7.30079 16.3864 27.10852 0 0 0 # C_R

50 1 4 0.112 7.23889 17.2506 27.45039 0 0 0 # H_

51 1 3 0.0333 8.99541 12.9936 27.86589 0 0 0 # C_R

52 1 3 -0.1428 9.1051 11.6528 27.51678 0 0 0 # C_R

53 1 4 0.1189 8.65649 11.32621 26.7691 0 0 0 # H_

54 1 3 -0.1406 9.88652 10.7968 28.28359 0 0 0 # C_R

55 1 4 0.1641 9.95961 9.89801 28.05132 0 0 0 # H_

56 1 3 0.033 10.55839 11.28111 29.39989 0 0 0 # C_R

57 1 3 -0.1304 10.44844 12.62228 29.74899 0 0 0 # C_R

58 1 4 0.15 10.89858 12.9481 30.4971 0 0 0 # H_

59 1 3 -0.1293 9.66699 13.47832 28.98219 0 0 0 # C_R

60 1 4 0.1131 9.59551 14.37621 29.21501 0 0 0 # H_

61 1 3 0.6131 11.3101 10.13678 30.04652 0 0 0 # C_R

62 1 3 0.0268 4.11731 20.37759 25.75352 0 0 0 # C_R

63 1 3 -0.1278 4.96027 21.04401 26.63369 0 0 0 # C_R

64 1 4 0.1188 5.81348 20.71062 26.803 0 0 0 # H_

65 1 3 -0.1257 4.53372 22.20631 27.2642 0 0 0 # C_R

66 1 4 0.1093 5.09818 22.65229 27.85239 0 0 0 # H_

67 1 3 0.0395 3.25923 22.70189 27.01449 0 0 0 # C_R

68 1 3 -0.1228 2.41637 22.03512 26.13009 0 0 0 # C_R

69 1 4 0.1108 1.56331 22.3685 25.96091 0 0 0 # H_

70 1 3 -0.1278 2.84818 20.87281 25.49958 0 0 0 # C_R

71 1 4 0.1213 2.28119 20.4313 24.91139 0 0 0 # H_

72 1 3 0.0356 2.72959 23.81378 27.71571 0 0 0 # C_R

73 1 3 -0.1346 3.5898 24.5207 28.54849 0 0 0 # C_R

74 1 4 0.1147 4.45311 24.2111 28.69448 0 0 0 # H_

75 1 3 -0.1429 3.15719 25.69071 29.16161 0 0 0 # C_R

76 1 4 0.1434 3.73258 26.16191 29.71848 0 0 0 # H_

77 1 3 0.0421 1.86522 26.1543 28.94199 0 0 0 # C_R

78 1 3 -0.1243 1.00559 25.44738 28.10921 0 0 0 # C_R

79 1 4 0.1595 0.14309 25.7564 27.96241 0 0 0 # H_

80 1 3 -0.1378 1.43789 24.27692 27.49651 0 0 0 # C_R

81 1 4 0.114 0.86359 23.80572 26.93841 0 0 0 # H_

82 1 3 0.6171 1.42017 27.3107 29.80739 0 0 0 # C_R

83 1 3 -0.1343 1.14082 20.68891 10.47071 0 0 0 # C_R

84 1 4 0.1568 1.94557 20.22852 10.39281 0 0 0 # H_

85 1 3 -0.136 1.16451 22.07071 10.4686 0 0 0 # C_R

86 1 4 0.1178 1.98201 22.50821 10.39281 0 0 0 # H_

87 1 3 -0.123 1.13101 25.1167 10.36742 0 0 0 # C_R

88 1 4 0.115 1.91441 24.67068 10.15161 0 0 0 # H_

89 1 3 -0.1224 1.14321 26.48489 10.4647 0 0 0 # C_R

90 1 4 0.1102 1.95077 26.94132 10.44782 0 0 0 # H_

91 1 3 -0.1376 0.20028 9.2943 9.42839 0 0 0 # C_R

92 1 4 0.1583 0.36678 9.7628 8.6452 0 0 0 # H_

93 1 3 -0.1394 0.17169 7.89322 9.42758 0 0 0 # C_R

94 1 4 0.1324 0.2627 7.4381 8.624 0 0 0 # H_

95 1 3 -0.1283 -1.10859 4.93319 10.9133 0 0 0 # C_R

96 1 4 0.1291 -1.9014 5.3838 11.08679 0 0 0 # H_

97 1 3 -0.1296 -1.0769 3.5772 10.9937 0 0 0 # C_R

98 1 4 0.1161 -1.80521 3.1086 11.3365 1 0 0 # H_

99 1 1 0.4538 11.32342 38.3511 10.91702 0 0 0 # Zn3+2

100 1 1 0.439 14.19041 37.86129 12.1471 0 0 0 # Zn3+2

101 1 2 -0.4846 12.9805 38.38998 13.6029 0 0 0 # O_2

102 1 2 -0.4778 11.04659 38.80501 12.81959 0 0 0 # O_2

103 1 2 -0.4782 -15.1241 5.122 30.96638 1 0 0 # O_2

104 1 2 -0.4833 -13.33867 5.22828 29.62158 1 0 0 # O_2

105 1 2 -0.4788 2.27599 31.45111 29.8159 0 0 0 # O_2

106 1 2 -0.4955 0.58111 32.80439 30.0359 0 0 0 # O_2

107 1 2 -0.4881 9.90993 37.02269 10.51172 0 0 0 # O_2

108 1 2 -0.4895 15.759 38.85321 11.66229 0 0 0 # O_2

109 1 3 0.0043 8.44502 40.24479 23.15019 0 0 0 # C_R

110 1 3 -0.1147 8.82189 41.39142 23.83961 0 0 0 # C_R

111 1 4 0.1053 9.34838 42.03391 23.42191 0 0 0 # H_

112 1 3 0.007 8.41008 41.57929 25.15352 0 0 0 # C_R

113 1 3 -0.1135 7.62151 40.62062 25.77848 0 0 0 # C_R

114 1 4 0.1097 7.34548 40.74541 26.65908 0 0 0 # H_

115 1 3 0.0027 7.24458 39.47399 25.0892 0 0 0 # C_R

116 1 3 -0.118 7.65629 39.28612 23.7752 0 0 0 # C_R

117 1 4 0.1125 7.40273 38.51978 23.31188 0 0 0 # H_

118 1 3 0.0308 9.86361 39.5718 19.1306 0 0 0 # C_R

119 1 3 -0.1297 10.66711 40.2543 20.03709 0 0 0 # C_R

120 1 4 0.1208 11.50209 40.5652 19.77431 0 0 0 # H_

121 1 3 -0.1291 10.22178 40.4678 21.33568 0 0 0 # C_R

122 1 4 0.1178 10.75818 40.92562 21.9408 0 0 0 # H_

123 1 3 0.0297 8.97351 39.9997 21.72931 0 0 0 # C_R

124 1 3 -0.1294 8.17081 39.3177 20.82328 0 0 0 # C_R

125 1 4 0.1198 7.33508 39.0063 21.08611 0 0 0 # H_

126 1 3 -0.1289 8.61539 39.1037 19.5238 0 0 0 # C_R

127 1 4 0.1224 8.07899 38.64588 18.91948 0 0 0 # H_

128 1 3 0.04 11.29418 38.91759 15.1047 0 0 0 # C_R

129 1 3 -0.1384 12.15439 38.8433 16.19391 0 0 0 # C_R

130 1 4 0.1534 13.04972 38.63241 16.0632 0 0 0 # H_

131 1 3 -0.141 11.67371 39.08109 17.47651 0 0 0 # C_R

132 1 4 0.1247 12.24861 39.02892 18.2043 0 0 0 # H_

133 1 3 0.0284 10.33308 39.39289 17.66951 0 0 0 # C_R

134 1 3 -0.1418 9.47292 39.46719 16.5803 0 0 0 # C_R

135 1 4 0.1259 8.5758 39.67758 16.71059 0 0 0 # H_

136 1 3 -0.135 9.95342 39.2298 15.2977 0 0 0 # C_R

137 1 4 0.1562 9.37699 39.28121 14.5694 0 0 0 # H_

138 1 3 0.6177 11.7988 38.47518 13.74419 0 0 0 # C_R

139 1 3 0.0327 8.77869 42.78169 25.9249 0 0 0 # C_R

140 1 3 -0.1237 9.85013 43.50568 25.41499 0 0 0 # C_R

141 1 4 0.1101 10.25611 43.23681 24.62368 0 0 0 # H_

142 1 3 -0.1229 10.31209 44.63019 26.0887 0 0 0 # C_R

143 1 4 0.1154 37.0397 0.0631 25.74929 0 0 0 # H_

144 1 3 0.0349 9.70312 45.0307 27.2722 0 0 0 # C_R

145 1 3 -0.1241 8.63178 44.3068 27.7821 0 0 0 # C_R

146 1 4 0.1075 8.22468 44.57482 28.57599 0 0 0 # H_

147 1 3 -0.1254 8.16948 43.1818 27.10852 0 0 0 # C_R

148 1 4 0.112 7.45223 42.69609 27.45039 0 0 0 # H_

149 1 3 0.0333 36.27159 1.2935 27.86589 0 0 0 # C_R

150 1 3 -0.1428 37.3778 2.0589 27.51678 0 0 0 # C_R

151 1 4 0.1189 37.88499 1.8336 26.7691 0 0 0 # H_

152 1 3 -0.1405 -14.29358 3.1636 28.28359 1 0 0 # C_R

153 1 4 0.1641 -13.55169 3.6763 28.05132 1 0 0 # H_

154 1 3 0.033 36.97311 3.5033 29.39989 0 0 0 # C_R

155 1 3 -0.1304 35.86661 2.7374 29.74899 0 0 0 # C_R

156 1 4 0.15 35.35938 2.9644 30.4971 0 0 0 # H_

157 1 3 -0.1293 35.51589 1.6327 28.98219 0 0 0 # C_R

158 1 4 0.1131 34.77411 1.1218 29.21501 0 0 0 # H_

159 1 3 0.613 -14.43379 4.7264 30.04652 1 0 0 # C_R

160 1 3 0.0268 6.30483 38.42918 25.75352 0 0 0 # C_R

161 1 3 -0.1278 5.30622 38.82609 26.63369 0 0 0 # C_R

162 1 4 0.1188 5.16839 39.73169 26.803 0 0 0 # H_

163 1 3 -0.1257 4.51288 37.87549 27.2642 0 0 0 # C_R

164 1 4 0.1093 3.8444 38.14129 27.85239 0 0 0 # H_

165 1 3 0.0394 4.72102 36.524 27.01449 0 0 0 # C_R

166 1 3 -0.1228 5.71982 36.1275 26.13009 0 0 0 # C_R

167 1 4 0.1108 5.8577 35.2219 25.96091 0 0 0 # H_

168 1 3 -0.1278 6.51061 37.08261 25.49958 0 0 0 # C_R

169 1 4 0.1213 7.17638 36.81229 24.91139 0 0 0 # H_

170 1 3 0.0356 4.02291 35.50938 27.71571 0 0 0 # C_R

171 1 3 -0.1346 2.9806 35.90088 28.54849 0 0 0 # C_R

172 1 4 0.1147 2.81699 36.80328 28.69448 0 0 0 # H_

173 1 3 -0.1429 2.1836 34.94131 29.16161 0 0 0 # C_R

174 1 4 0.1434 1.4878 35.20388 29.71848 0 0 0 # H_

175 1 3 0.0421 2.42807 33.5906 28.94199 0 0 0 # C_R

176 1 3 -0.1243 3.4701 33.1995 28.10921 0 0 0 # C_R

177 1 4 0.1595 3.63371 32.298 27.96241 0 0 0 # H_

178 1 3 -0.1378 4.2676 34.15921 27.49651 0 0 0 # C_R

179 1 4 0.114 4.9629 33.89741 26.93841 0 0 0 # H_

180 1 3 0.6171 1.64912 32.62688 29.80739 0 0 0 # C_R

181 1 3 -0.1343 7.52342 35.6959 10.47071 0 0 0 # C_R

182 1 4 0.1568 7.51981 36.62312 10.39281 0 0 0 # H_

183 1 3 -0.1359 6.315 35.02552 10.4686 0 0 0 # C_R

184 1 4 0.1178 5.52729 35.51479 10.39281 0 0 0 # H_

185 1 3 -0.123 3.6938 33.47351 10.36742 0 0 0 # C_R

186 1 4 0.115 3.68839 34.37501 10.15161 0 0 0 # H_

187 1 3 -0.1223 2.5028 32.79988 10.4647 0 0 0 # C_R

188 1 4 0.1102 1.70372 33.27118 10.44782 0 0 0 # H_

189 1 3 -0.1376 -34.16022 40.57872 9.42839 1 0 0 # C_R

190 1 4 0.1583 -34.64933 40.48861 8.6452 1 0 0 # H_

191 1 3 -0.1394 -32.9325 41.2545 9.42758 1 0 0 # C_R

192 1 4 0.1324 -32.58403 41.56081 8.624 1 0 0 # H_

193 1 3 -0.1283 -29.72901 41.62569 10.9133 1 0 0 # C_R

194 1 4 0.129 -29.7228 40.71378 11.08679 1 0 0 # H_

195 1 3 -0.1296 -28.57048 42.33121 10.9937 1 0 0 # C_R

196 1 4 0.1161 -27.80058 41.9347 11.3365 1 0 0 # H_

197 1 1 0.4538 -12.86379 35.6831 10.91702 0 0 0 # Zn3+2

198 1 1 0.439 -13.87299 38.41089 12.1471 0 0 0 # Zn3+2

199 1 2 -0.4846 -13.72598 37.09878 13.6029 0 0 0 # O_2

200 1 2 -0.4778 -13.11841 35.21649 12.81959 0 0 0 # O_2

201 1 2 -0.4782 -22.88469 29.39352 30.96638 1 0 0 # O_2

202 1 2 -0.4833 -23.86949 30.8866 29.62158 1 0 0 # O_2

203 1 2 -0.4788 -2.3644 31.29788 29.8159 0 0 0 # O_2

204 1 2 -0.4955 -2.68902 29.15339 30.0359 0 0 0 # O_2

205 1 2 -0.4881 -11.00661 35.12328 10.51172 0 0 0 # O_2

206 1 2 -0.4895 -15.51629 39.27351 11.66229 0 0 0 # O_2

207 1 3 0.0043 -13.06452 32.24349 23.15019 0 0 0 # C_R

208 1 3 -0.1147 -14.246 31.9966 23.83961 0 0 0 # C_R

209 1 4 0.1053 -15.0656 32.1314 23.42191 0 0 0 # H_

210 1 3 0.007 -14.20281 31.54612 25.15352 0 0 0 # C_R

211 1 3 -0.1135 -12.97819 31.34249 25.77848 0 0 0 # C_R

212 1 4 0.1097 -12.94829 31.04109 26.65908 0 0 0 # H_

213 1 3 0.0027 -11.79679 31.58942 25.0892 0 0 0 # C_R

214 1 3 -0.118 -11.8399 32.0399 23.7752 0 0 0 # C_R

215 1 4 0.1125 -11.0495 32.20339 23.31188 0 0 0 # H_

216 1 3 0.0308 -13.19098 33.8087 19.1306 0 0 0 # C_R

217 1 3 -0.1296 -14.1838 34.16321 20.03709 0 0 0 # C_R

218 1 4 0.1208 -14.87051 34.73092 19.77431 0 0 0 # H_

219 1 3 -0.1291 -14.14608 33.67079 21.33568 0 0 0 # C_R

220 1 4 0.1178 -14.81071 33.90642 21.9408 0 0 0 # H_

221 1 3 0.0297 -13.11662 32.82381 21.72931 0 0 0 # C_R

222 1 3 -0.1294 -12.12451 32.4697 20.82328 0 0 0 # C_R

223 1 4 0.1198 -11.43698 31.90159 21.08611 0 0 0 # H_

224 1 3 -0.1289 -12.16142 32.96171 19.5238 0 0 0 # C_R

225 1 4 0.1224 -11.49689 32.726 18.91948 0 0 0 # H_

226 1 3 0.04 -13.33972 35.37472 15.1047 0 0 0 # C_R

227 1 3 -0.1384 -13.70551 36.15678 16.19391 0 0 0 # C_R

228 1 4 0.1534 -13.97048 37.0376 16.0632 0 0 0 # H_

229 1 3 -0.141 -13.6711 35.62161 17.47651 0 0 0 # C_R

230 1 4 0.1247 -13.91334 36.14552 18.2043 0 0 0 # H_

231 1 3 0.0284 -13.27081 34.30468 17.66951 0 0 0 # C_R

232 1 3 -0.1418 -12.90512 33.52262 16.5803 0 0 0 # C_R

233 1 4 0.1259 -12.63872 32.6404 16.71059 0 0 0 # H_

234 1 3 -0.135 -12.93972 34.0573 15.2977 0 0 0 # C_R

235 1 4 0.1562 -12.69597 33.53248 14.5694 0 0 0 # H_

236 1 3 0.6177 -13.20891 36.03289 13.74419 0 0 0 # C_R

237 1 3 0.0327 -15.4284 31.26409 25.9249 0 0 0 # C_R

238 1 3 -0.1237 -16.5911 31.83 25.41499 0 0 0 # C_R

239 1 4 0.1101 -16.56121 32.31611 24.62368 0 0 0 # H_

240 1 3 -0.1229 -17.7959 31.66781 26.0887 0 0 0 # C_R

241 1 4 0.1154 -18.57448 32.0458 25.74929 1 0 0 # H_

242 1 3 0.0349 -17.83831 30.94021 27.2722 0 0 0 # C_R

243 1 3 -0.1241 -16.6757 30.37431 27.7821 0 0 0 # C_R

244 1 4 0.1075 -16.7043 29.8877 28.57599 0 0 0 # H_

245 1 3 -0.1254 -15.4703 30.5365 27.10852 0 0 0 # C_R

246 1 4 0.112 -14.691 30.1581 27.45039 0 0 0 # H_

247 1 3 0.0332 -19.25592 30.76541 27.86589 1 0 0 # C_R

248 1 3 -0.1428 -20.47201 31.34068 27.51678 1 0 0 # C_R

249 1 4 0.1189 -20.53051 31.89262 26.7691 1 0 0 # H_

250 1 3 -0.1406 -21.60401 31.09199 28.28359 1 0 0 # C_R

251 1 4 0.1641 -22.41888 31.47809 28.05132 1 0 0 # H_

252 1 3 0.033 -21.52051 30.26799 29.39989 1 0 0 # C_R

253 1 3 -0.1304 -20.3039 29.69271 29.74899 1 0 0 # C_R

254 1 4 0.15 -20.24699 29.13992 30.4971 1 0 0 # H_

255 1 3 -0.1293 -19.1719 29.9414 28.98219 1 0 0 # C_R

256 1 4 0.1131 -18.35859 29.5544 29.21501 1 0 0 # H_

257 1 3 0.613 -22.88731 30.1891 30.04652 1 0 0 # C_R

258 1 3 0.0268 -10.42209 31.29788 25.75352 0 0 0 # C_R

259 1 3 -0.1278 -10.26652 30.2346 26.63369 0 0 0 # C_R

260 1 4 0.1188 -10.98179 29.66248 26.803 0 0 0 # H_

261 1 3 -0.1257 -9.04663 30.0229 27.2642 0 0 0 # C_R

262 1 4 0.1093 -8.94261 29.31112 27.85239 0 0 0 # H_

263 1 3 0.0394 -7.98017 30.8789 27.01449 0 0 0 # C_R

264 1 3 -0.1228 -8.13621 31.94209 26.13009 0 0 0 # C_R

265 1 4 0.1108 -7.42089 32.5143 25.96091 0 0 0 # H_

266 1 3 -0.1278 -9.35884 32.14942 25.49958 0 0 0 # C_R

267 1 4 0.1213 -9.4576 32.8612 24.91139 0 0 0 # H_

268 1 3 0.0356 -6.75251 30.78158 27.71571 0 0 0 # C_R

269 1 3 -0.1346 -6.57038 29.68321 28.54849 0 0 0 # C_R

270 1 4 0.1147 -7.27013 29.09032 28.69448 0 0 0 # H_

271 1 3 -0.1429 -5.34079 29.47281 29.16161 0 0 0 # C_R

272 1 4 0.1434 -5.22041 28.73891 29.71848 0 0 0 # H_

273 1 3 0.0421 -4.29338 30.35989 28.94199 0 0 0 # C_R

274 1 3 -0.1243 -4.47571 31.45782 28.10921 0 0 0 # C_R

275 1 4 0.1595 -3.77682 32.0503 27.96241 0 0 0 # H_

276 1 3 -0.1378 -5.70549 31.66871 27.49651 0 0 0 # C_R

277 1 4 0.114 -5.82649 32.40171 26.93841 0 0 0 # H_

278 1 3 0.6171 -3.06932 30.16711 29.80739 0 0 0 # C_R

279 1 3 -0.1343 -8.66432 33.7199 10.47071 0 0 0 # C_R

280 1 4 0.1568 -9.46538 33.2532 10.39281 0 0 0 # H_

281 1 3 -0.136 -7.47949 33.00848 10.4686 0 0 0 # C_R

282 1 4 0.1178 -7.50938 32.0818 10.39281 0 0 0 # H_

283 1 3 -0.1229 -4.82478 31.51459 10.36742 0 0 0 # C_R

284 1 4 0.115 -5.60282 31.05911 10.15161 0 0 0 # H_

285 1 3 -0.1224 -3.64596 30.81988 10.4647 0 0 0 # C_R

286 1 4 0.1102 -3.65452 29.89229 10.44782 0 0 0 # H_

287 1 3 -0.1376 -18.06201 40.23181 9.42839 0 0 0 # C_R

288 1 4 0.1583 -17.73948 39.85328 8.6452 0 0 0 # H_

289 1 3 -0.1394 -19.26111 40.95711 9.42758 0 0 0 # C_R

290 1 4 0.1324 -19.70069 41.10579 8.624 0 0 0 # H_

291 1 3 -0.1283 -21.18441 43.54582 10.9133 0 0 0 # C_R

292 1 4 0.129 -20.3978 44.0072 11.08679 0 0 0 # H_

293 1 3 -0.1296 -22.37469 44.19638 10.9937 0 0 0 # C_R

294 1 4 0.1161 3.5947 0.009 11.3365 0 0 0 # H_

295 1 1 0.4538 12.86379 35.6831 10.24098 0 0 0 # Zn3+2

296 1 1 0.439 13.87299 38.41089 9.0109 0 0 0 # Zn3+2

297 1 1 0.4482 -1.12682 28.601 30.81028 0 0 0 # Zn3+2

298 1 2 -0.4846 13.72603 37.09878 7.5551 0 0 0 # O_2

299 1 2 -0.4777 13.11841 35.21649 8.33841 0 0 0 # O_2

300 1 2 -0.4782 -29.13731 29.39352 32.50762 1 0 0 # O_2

301 1 2 -0.4833 -28.15251 30.8866 33.85242 1 0 0 # O_2

302 1 2 -0.451 1.92299 30.13959 34.23699 0 0 0 # O_2

303 1 2 -0.4521 3.01728 29.77511 32.43729 0 0 0 # O_2

304 1 2 -0.4881 11.00661 35.12328 10.64628 0 0 0 # O_2

305 1 2 -0.4895 15.51629 39.27351 9.49571 0 0 0 # O_2

306 1 3 0.0043 13.06452 32.24349 40.32381 0 0 0 # C_R

307 1 3 -0.1146 14.24599 31.9966 39.63439 0 0 0 # C_R

308 1 4 0.1053 15.0656 32.1314 40.05209 0 0 0 # H_

309 1 3 0.007 14.20279 31.54612 38.32048 0 0 0 # C_R

310 1 3 -0.1135 12.97819 31.34249 37.69552 0 0 0 # C_R

311 1 4 0.1098 12.94833 31.04109 36.81492 0 0 0 # H_

312 1 3 0.0027 11.79679 31.58942 38.3848 0 0 0 # C_R

313 1 3 -0.118 11.8399 32.0399 39.6988 0 0 0 # C_R

314 1 4 0.1125 11.0495 32.20339 40.16212 0 0 0 # H_

315 1 3 0.0308 13.19098 33.8087 2.0274 0 0 0 # C_R

316 1 3 -0.1296 14.1838 34.16321 1.121 0 0 0 # C_R

317 1 4 0.1208 14.87046 34.73092 1.3837 0 0 0 # H_

318 1 3 -0.1291 14.14608 33.67079 42.13832 0 0 0 # C_R

319 1 4 0.1178 14.81072 33.90642 41.5332 0 0 0 # H_

320 1 3 0.0297 13.11662 32.82381 41.74469 0 0 0 # C_R

321 1 3 -0.1293 12.12451 32.4697 0.3347 0 0 0 # C_R

322 1 4 0.1199 11.43698 31.90159 0.0719 0 0 0 # H_

323 1 3 -0.1289 12.16142 32.96171 1.6342 0 0 0 # C_R

324 1 4 0.1224 11.49689 32.726 2.2385 0 0 0 # H_

325 1 3 0.04 13.33972 35.37472 6.0533 0 0 0 # C_R

326 1 3 -0.1384 13.70551 36.15678 4.96409 0 0 0 # C_R

327 1 4 0.1534 13.97048 37.0376 5.0948 0 0 0 # H_

328 1 3 -0.141 13.6711 35.62161 3.6815 0 0 0 # C_R

329 1 4 0.1247 13.91328 36.14552 2.9537 0 0 0 # H_

330 1 3 0.0284 13.27081 34.30468 3.4885 0 0 0 # C_R

331 1 3 -0.1417 12.90507 33.52262 4.5777 0 0 0 # C_R

332 1 4 0.1259 12.63872 32.6404 4.44741 0 0 0 # H_

333 1 3 -0.135 12.93972 34.0573 5.8603 0 0 0 # C_R

334 1 4 0.1563 12.69602 33.53248 6.5886 0 0 0 # H_

335 1 3 0.6177 13.20891 36.03289 7.41381 0 0 0 # C_R

336 1 3 0.0327 15.42842 31.26409 37.5491 0 0 0 # C_R

337 1 3 -0.1237 16.59109 31.83 38.05901 0 0 0 # C_R

338 1 4 0.1101 16.56118 32.31611 38.85032 0 0 0 # H_

339 1 3 -0.1229 17.79592 31.66781 37.3853 0 0 0 # C_R

340 1 4 0.1154 18.57448 32.0458 37.72471 0 0 0 # H_

341 1 3 0.0349 17.83832 30.94021 36.2018 0 0 0 # C_R

342 1 3 -0.1241 16.6757 30.37431 35.6919 0 0 0 # C_R

343 1 4 0.1075 16.70429 29.8877 34.89801 0 0 0 # H_

344 1 3 -0.1253 15.4703 30.5365 36.36548 0 0 0 # C_R

345 1 4 0.1121 14.69099 30.1581 36.02361 0 0 0 # H_

346 1 3 0.0332 19.25592 30.76541 35.60811 0 0 0 # C_R

347 1 3 -0.1428 20.47201 31.34068 35.95722 0 0 0 # C_R

348 1 4 0.1189 20.53051 31.89262 36.7049 0 0 0 # H_

349 1 3 -0.1406 -30.41799 31.09199 35.19041 1 0 0 # C_R

350 1 4 0.1641 -29.60312 31.47809 35.42268 1 0 0 # H_

351 1 3 0.033 21.52051 30.26799 34.07411 0 0 0 # C_R

352 1 3 -0.1304 20.3039 29.69271 33.72501 0 0 0 # C_R

353 1 4 0.15 20.24699 29.13992 32.9769 0 0 0 # H_

354 1 3 -0.1293 19.1719 29.9414 34.49181 0 0 0 # C_R

355 1 4 0.1131 18.35859 29.5544 34.25899 0 0 0 # H_

356 1 3 0.613 -29.13469 30.1891 33.42748 1 0 0 # C_R

357 1 3 0.0269 10.42209 31.29788 37.72048 0 0 0 # C_R

358 1 3 -0.1276 10.26652 30.2346 36.84031 0 0 0 # C_R

359 1 4 0.1188 10.98179 29.66248 36.671 0 0 0 # H_

360 1 3 -0.1256 9.04657 30.0229 36.2098 0 0 0 # C_R

361 1 4 0.1094 8.94261 29.31112 35.62161 0 0 0 # H_

362 1 3 0.0383 7.98023 30.8789 36.45951 0 0 0 # C_R

363 1 3 -0.1229 8.13621 31.94209 37.34391 0 0 0 # C_R

364 1 4 0.1104 7.42089 32.5143 37.51309 0 0 0 # H_

365 1 3 -0.1276 9.35878 32.14942 37.97442 0 0 0 # C_R

366 1 4 0.1212 9.4576 32.8612 38.56261 0 0 0 # H_

367 1 3 0.0361 6.75251 30.78158 35.75829 0 0 0 # C_R

368 1 3 -0.1296 6.57038 29.68321 34.92551 0 0 0 # C_R

369 1 4 0.1132 7.27007 29.09032 34.77952 0 0 0 # H_

370 1 3 -0.1331 5.34079 29.47281 34.31239 0 0 0 # C_R

371 1 4 0.1381 5.22041 28.73891 33.75552 0 0 0 # H_

372 1 3 0.0233 4.29343 30.35989 34.53201 0 0 0 # C_R

373 1 3 -0.1351 4.47571 31.45782 35.36479 0 0 0 # C_R

374 1 4 0.1384 3.77682 32.0503 35.51159 0 0 0 # H_

375 1 3 -0.1297 5.70549 31.66871 35.97749 0 0 0 # C_R

376 1 4 0.1137 5.82649 32.40171 36.53559 0 0 0 # H_

377 1 3 0.5777 3.06927 30.16711 33.66661 0 0 0 # C_R

378 1 3 -0.1343 8.66432 33.7199 10.68729 0 0 0 # C_R

379 1 4 0.1568 9.46538 33.2532 10.76519 0 0 0 # H_

380 1 3 -0.136 7.47949 33.00848 10.6894 0 0 0 # C_R

381 1 4 0.1178 7.50943 32.0818 10.76519 0 0 0 # H_

382 1 3 -0.123 4.82483 31.51459 10.79058 0 0 0 # C_R

383 1 4 0.115 5.60282 31.05911 11.00639 0 0 0 # H_

384 1 3 -0.1224 3.64601 30.81988 10.6933 0 0 0 # C_R

385 1 4 0.1102 3.65452 29.89229 10.71018 0 0 0 # H_

386 1 3 -0.1376 -33.95999 40.23181 11.72961 1 0 0 # C_R

387 1 4 0.1583 -34.28247 39.85328 12.5128 1 0 0 # H_

388 1 3 -0.1395 -32.76091 40.95711 11.73042 1 0 0 # C_R

389 1 4 0.1324 -32.32127 41.10579 12.534 1 0 0 # H_

390 1 3 -0.1283 -30.8376 43.54582 10.2447 1 0 0 # C_R

391 1 4 0.129 -31.6242 44.0072 10.07121 1 0 0 # H_

392 1 3 -0.1296 -29.64729 44.19638 10.1643 1 0 0 # C_R

393 1 4 0.1161 -3.59471 0.009 9.8215 1 0 0 # H_

394 1 1 0.4538 -1.5404 16.0705 10.24098 0 0 0 # Zn3+2

395 1 1 0.439 0.31728 13.83252 9.0109 0 0 0 # Zn3+2

396 1 1 0.4482 -0.67837 31.72768 30.81028 0 0 0 # Zn3+2

397 1 2 -0.4846 -0.74548 14.61589 7.5551 0 0 0 # O_2

398 1 2 -0.4777 -2.0718 16.0832 8.33841 0 0 0 # O_2

399 1 2 -0.4782 -11.99781 10.5368 32.50762 1 0 0 # O_2

400 1 2 -0.4833 -11.19722 8.93749 33.85242 1 0 0 # O_2

401 1 2 -0.451 -0.8708 28.31722 34.23699 0 0 0 # O_2

402 1 2 -0.4521 -1.73358 27.55178 32.43729 0 0 0 # O_2

403 1 2 -0.4881 -1.0966 17.95882 10.64628 0 0 0 # O_2

404 1 2 -0.4895 0.24271 11.97812 9.49571 0 0 0 # O_2

405 1 3 0.0043 -4.61961 17.61638 40.32381 0 0 0 # C_R

406 1 3 -0.1146 -5.42409 16.71668 39.63439 0 0 0 # C_R

407 1 4 0.1053 -5.71719 15.93948 40.05209 0 0 0 # H_

408 1 3 0.007 -5.7926 16.97929 38.32048 0 0 0 # C_R

409 1 3 -0.1135 -5.35669 18.14169 37.69552 0 0 0 # C_R

410 1 4 0.1098 -5.6028 18.3183 36.81492 0 0 0 # H_

411 1 3 0.0027 -4.55219 19.04139 38.3848 0 0 0 # C_R

412 1 3 -0.118 -4.18358 18.77869 39.6988 0 0 0 # C_R

413 1 4 0.1125 -3.64672 19.38149 40.16212 0 0 0 # H_

414 1 3 0.0308 -3.3273 16.7243 2.0274 0 0 0 # C_R

415 1 3 -0.1297 -3.51671 15.68719 1.121 0 0 0 # C_R

416 1 4 0.1208 -3.36841 14.80872 1.3837 0 0 0 # H_

417 1 3 -0.1291 -3.92433 15.96611 42.13832 0 0 0 # C_R

418 1 4 0.1178 -4.0525 15.2728 41.5332 0 0 0 # H_

419 1 3 0.0297 -4.14298 17.28119 41.74469 0 0 0 # C_R

420 1 3 -0.1293 -3.95367 18.31739 0.3347 0 0 0 # C_R

421 1 4 0.1199 -4.10193 19.19682 0.0719 0 0 0 # H_

422 1 3 -0.1288 -3.54613 18.03942 1.6342 0 0 0 # C_R

423 1 4 0.1224 -3.41782 18.73282 2.2385 0 0 0 # H_

424 1 3 0.04 -2.04551 15.81248 6.0533 0 0 0 # C_R

425 1 3 -0.1384 -1.55098 15.10471 4.96409 0 0 0 # C_R

426 1 4 0.1534 -0.92079 14.43478 5.0948 0 0 0 # H_

427 1 3 -0.141 -1.99741 15.4021 3.6815 0 0 0 # C_R

428 1 4 0.1246 -1.66468 14.9304 2.9537 0 0 0 # H_

429 1 3 0.0284 -2.93771 16.40722 3.4885 0 0 0 # C_R

430 1 3 -0.1417 -3.43218 17.1149 4.5777 0 0 0 # C_R

431 1 4 0.1259 -4.06289 17.78672 4.44741 0 0 0 # H_

432 1 3 -0.135 -2.98632 16.8176 5.8603 0 0 0 # C_R

433 1 4 0.1563 -3.319 17.2911 6.5886 0 0 0 # H_

434 1 3 0.6177 -1.41011 15.59668 7.41381 0 0 0 # C_R

435 1 3 0.0327 -6.64971 16.05892 37.5491 0 0 0 # C_R

436 1 3 -0.1237 -6.74101 14.76911 38.05901 0 0 0 # C_R

437 1 4 0.1101 -6.30511 14.55192 38.85032 0 0 0 # H_

438 1 3 -0.1229 -7.4839 13.8068 37.3853 0 0 0 # C_R

439 1 4 0.1154 -7.54582 12.9435 37.72471 1 0 0 # H_

440 1 3 0.0349 -8.13519 14.13379 36.2018 0 0 0 # C_R

441 1 3 -0.1241 -8.04389 15.42368 35.6919 0 0 0 # C_R

442 1 4 0.1075 -8.47959 15.64218 34.89801 0 0 0 # H_

443 1 3 -0.1253 -7.3008 16.3864 36.36548 0 0 0 # C_R

444 1 4 0.1121 -7.2389 17.2506 36.02361 0 0 0 # H_

445 1 3 0.0332 -8.99541 12.9936 35.60811 1 0 0 # C_R

446 1 3 -0.1428 -9.1051 11.6528 35.95722 1 0 0 # C_R

447 1 4 0.119 -8.65649 11.32621 36.7049 1 0 0 # H_

448 1 3 -0.1406 -9.88652 10.7968 35.19041 1 0 0 # C_R

449 1 4 0.1641 -9.95961 9.89801 35.42268 1 0 0 # H_

450 1 3 0.033 -10.55839 11.28111 34.07411 1 0 0 # C_R

451 1 3 -0.1304 -10.44838 12.62228 33.72501 1 0 0 # C_R

452 1 4 0.15 -10.89858 12.9481 32.9769 1 0 0 # H_

453 1 3 -0.1293 -9.66699 13.47832 34.49181 1 0 0 # C_R

454 1 4 0.1131 -9.59551 14.37621 34.25899 1 0 0 # H_

455 1 3 0.6131 -11.3101 10.13678 33.42748 1 0 0 # C_R

456 1 3 0.0269 -4.11731 20.37759 37.72048 0 0 0 # C_R

457 1 3 -0.1276 -4.96032 21.04401 36.84031 0 0 0 # C_R

458 1 4 0.1188 -5.81354 20.71062 36.671 0 0 0 # H_

459 1 3 -0.1256 -4.53372 22.20631 36.2098 0 0 0 # C_R

460 1 4 0.1094 -5.09818 22.65229 35.62161 0 0 0 # H_

461 1 3 0.0383 -3.25918 22.70189 36.45951 0 0 0 # C_R

462 1 3 -0.1229 -2.41642 22.03512 37.34391 0 0 0 # C_R

463 1 4 0.1104 -1.56331 22.3685 37.51309 0 0 0 # H_

464 1 3 -0.1276 -2.84823 20.87281 37.97442 0 0 0 # C_R

465 1 4 0.1212 -2.28119 20.4313 38.56261 0 0 0 # H_

466 1 3 0.0362 -2.72959 23.81378 35.75829 0 0 0 # C_R

467 1 3 -0.1296 -3.5898 24.5207 34.92551 0 0 0 # C_R

468 1 4 0.1132 -4.45311 24.2111 34.77952 0 0 0 # H_

469 1 3 -0.1331 -3.15719 25.69071 34.31239 0 0 0 # C_R

470 1 4 0.1381 -3.73263 26.16191 33.75552 0 0 0 # H_

471 1 3 0.0233 -1.86522 26.1543 34.53201 0 0 0 # C_R

472 1 3 -0.1351 -1.00559 25.44738 35.36479 0 0 0 # C_R

473 1 4 0.1384 -0.14309 25.7564 35.51159 0 0 0 # H_

474 1 3 -0.1297 -1.43789 24.27692 35.97749 0 0 0 # C_R

475 1 4 0.1137 -0.86359 23.80572 36.53559 0 0 0 # H_

476 1 3 0.5777 -1.42023 27.3107 33.66661 0 0 0 # C_R

477 1 3 -0.1343 -1.14082 20.68891 10.68729 0 0 0 # C_R

478 1 4 0.1568 -1.94562 20.22852 10.76519 0 0 0 # H_

479 1 3 -0.136 -1.16451 22.07071 10.6894 0 0 0 # C_R

480 1 4 0.1178 -1.98201 22.50821 10.76519 0 0 0 # H_

481 1 3 -0.123 -1.13101 25.1167 10.79058 0 0 0 # C_R

482 1 4 0.115 -1.91441 24.67068 11.00639 0 0 0 # H_

483 1 3 -0.1224 -1.14321 26.48489 10.6933 0 0 0 # C_R

484 1 4 0.1102 -1.95083 26.94132 10.71018 0 0 0 # H_

485 1 3 -0.1376 -0.2003 9.2943 11.72961 0 0 0 # C_R

486 1 4 0.1583 -0.36678 9.7628 12.5128 0 0 0 # H_

487 1 3 -0.1395 -0.17171 7.89322 11.73042 0 0 0 # C_R

488 1 4 0.1324 -0.2627 7.4381 12.534 0 0 0 # H_

489 1 3 -0.1283 1.1086 4.93319 10.2447 0 0 0 # C_R

490 1 4 0.129 1.9014 5.3838 10.07121 0 0 0 # H_

491 1 3 -0.1296 1.0769 3.5772 10.1643 0 0 0 # C_R

492 1 4 0.1161 1.8052 3.1086 9.8215 0 0 0 # H_

493 1 1 0.4538 -11.32342 38.3511 10.24098 0 0 0 # Zn3+2

494 1 1 0.439 -14.19041 37.86129 9.0109 0 0 0 # Zn3+2

495 1 1 0.4482 1.80522 29.77601 30.81028 0 0 0 # Zn3+2

496 1 2 -0.4846 -12.9805 38.38998 7.5551 0 0 0 # O_2

497 1 2 -0.4777 -11.04659 38.80501 8.33841 0 0 0 # O_2

498 1 2 -0.4782 15.1241 5.122 32.50762 0 0 0 # O_2

499 1 2 -0.4833 13.33873 5.22828 33.85242 0 0 0 # O_2

500 1 2 -0.451 -1.05212 31.6479 34.23699 0 0 0 # O_2

501 1 2 -0.4521 -1.28362 32.7779 32.43729 0 0 0 # O_2

502 1 2 -0.4881 -9.90988 37.02269 10.64628 0 0 0 # O_2

503 1 2 -0.4895 -15.759 38.85321 9.49571 0 0 0 # O_2

504 1 3 0.0043 -8.44502 40.24479 40.32381 0 0 0 # C_R

505 1 3 -0.1146 -8.82189 41.39142 39.63439 0 0 0 # C_R

506 1 4 0.1053 -9.34843 42.03391 40.05209 0 0 0 # H_

507 1 3 0.007 -8.41008 41.57929 38.32048 0 0 0 # C_R

508 1 3 -0.1135 -7.62151 40.62062 37.69552 0 0 0 # C_R

509 1 4 0.1098 -7.34553 40.74541 36.81492 0 0 0 # H_

510 1 3 0.0027 -7.24458 39.47399 38.3848 0 0 0 # C_R

511 1 3 -0.118 -7.65629 39.28612 39.6988 0 0 0 # C_R

512 1 4 0.1125 -7.40268 38.51978 40.16212 0 0 0 # H_

513 1 3 0.0308 -9.86361 39.5718 2.0274 0 0 0 # C_R

514 1 3 -0.1296 -10.66711 40.2543 1.121 0 0 0 # C_R

515 1 4 0.1208 -11.50209 40.5652 1.3837 0 0 0 # H_

516 1 3 -0.1291 -10.22178 40.4678 42.13832 0 0 0 # C_R

517 1 4 0.1178 -10.75823 40.92562 41.5332 0 0 0 # H_

518 1 3 0.0297 -8.97351 39.9997 41.74469 0 0 0 # C_R

519 1 3 -0.1293 -8.17081 39.3177 0.3347 0 0 0 # C_R

520 1 4 0.1199 -7.33508 39.0063 0.0719 0 0 0 # H_

521 1 3 -0.1288 -8.61539 39.1037 1.6342 0 0 0 # C_R

522 1 4 0.1224 -8.07899 38.64588 2.2385 0 0 0 # H_

523 1 3 0.04 -11.29418 38.91759 6.0533 0 0 0 # C_R

524 1 3 -0.1384 -12.15439 38.8433 4.96409 0 0 0 # C_R

525 1 4 0.1534 -13.04972 38.63241 5.0948 0 0 0 # H_

526 1 3 -0.141 -11.67371 39.08109 3.6815 0 0 0 # C_R

527 1 4 0.1246 -12.24861 39.02892 2.9537 0 0 0 # H_

528 1 3 0.0284 -10.33308 39.39289 3.4885 0 0 0 # C_R

529 1 3 -0.1417 -9.47287 39.46719 4.5777 0 0 0 # C_R

530 1 4 0.1259 -8.5758 39.67758 4.44741 0 0 0 # H_

531 1 3 -0.1349 -9.95342 39.2298 5.8603 0 0 0 # C_R

532 1 4 0.1563 -9.37699 39.28121 6.5886 0 0 0 # H_

533 1 3 0.6177 -11.7988 38.47518 7.41381 0 0 0 # C_R

534 1 3 0.0327 -8.77869 42.78169 37.5491 0 0 0 # C_R

535 1 3 -0.1237 -9.85008 43.50568 38.05901 0 0 0 # C_R

536 1 4 0.1101 -10.25611 43.23681 38.85032 0 0 0 # H_

537 1 3 -0.1229 -10.31209 44.63019 37.3853 0 0 0 # C_R

538 1 4 0.1154 14.98232 0.0631 37.72471 0 0 0 # H_

539 1 3 0.0349 -9.70312 45.0307 36.2018 0 0 0 # C_R

540 1 3 -0.1241 -8.63178 44.3068 35.6919 0 0 0 # C_R

541 1 4 0.1075 -8.22473 44.57482 34.89801 0 0 0 # H_

542 1 3 -0.1254 -8.16948 43.1818 36.36548 0 0 0 # C_R

543 1 4 0.1121 -7.45218 42.69609 36.02361 0 0 0 # H_

544 1 3 0.0332 15.75041 1.2935 35.60811 0 0 0 # C_R

545 1 3 -0.1428 14.64419 2.0589 35.95722 0 0 0 # C_R

546 1 4 0.1189 14.137 1.8336 36.7049 0 0 0 # H_

547 1 3 -0.1405 14.2936 3.1636 35.19041 0 0 0 # C_R

548 1 4 0.1641 13.55172 3.6763 35.42268 0 0 0 # H_

549 1 3 0.033 15.04891 3.5033 34.07411 0 0 0 # C_R

550 1 3 -0.1304 16.15542 2.7374 33.72501 0 0 0 # C_R

551 1 4 0.15 16.66262 2.9644 32.9769 0 0 0 # H_

552 1 3 -0.1293 16.50611 1.6327 34.49181 0 0 0 # C_R

553 1 4 0.1131 17.2479 1.1218 34.25899 0 0 0 # H_

554 1 3 0.613 14.43379 4.7264 33.42748 0 0 0 # C_R

555 1 3 0.0269 -6.30478 38.42918 37.72048 0 0 0 # C_R

556 1 3 -0.1276 -5.30622 38.82609 36.84031 0 0 0 # C_R

557 1 4 0.1188 -5.16839 39.73169 36.671 0 0 0 # H_

558 1 3 -0.1256 -4.51288 37.87549 36.2098 0 0 0 # C_R

559 1 4 0.1094 -3.8444 38.14129 35.62161 0 0 0 # H_

560 1 3 0.0383 -4.72102 36.524 36.45951 0 0 0 # C_R

561 1 3 -0.1229 -5.71982 36.1275 37.34391 0 0 0 # C_R

562 1 4 0.1104 -5.8577 35.2219 37.51309 0 0 0 # H_

563 1 3 -0.1276 -6.51061 37.08261 37.97442 0 0 0 # C_R

564 1 4 0.1212 -7.17638 36.81229 38.56261 0 0 0 # H_

565 1 3 0.0361 -4.02291 35.50938 35.75829 0 0 0 # C_R

566 1 3 -0.1296 -2.9806 35.90088 34.92551 0 0 0 # C_R

567 1 4 0.1132 -2.81699 36.80328 34.77952 0 0 0 # H_

568 1 3 -0.1331 -2.1836 34.94131 34.31239 0 0 0 # C_R

569 1 4 0.1381 -1.4878 35.20388 33.75552 0 0 0 # H_

570 1 3 0.0233 -2.42807 33.5906 34.53201 0 0 0 # C_R

571 1 3 -0.1351 -3.4701 33.1995 35.36479 0 0 0 # C_R

572 1 4 0.1384 -3.63371 32.298 35.51159 0 0 0 # H_

573 1 3 -0.1297 -4.2676 34.15921 35.97749 0 0 0 # C_R

574 1 4 0.1137 -4.9629 33.89741 36.53559 0 0 0 # H_

575 1 3 0.5777 -1.64907 32.62688 33.66661 0 0 0 # C_R

576 1 3 -0.1343 -7.52342 35.6959 10.68729 0 0 0 # C_R

577 1 4 0.1568 -7.51981 36.62312 10.76519 0 0 0 # H_

578 1 3 -0.1359 -6.315 35.02552 10.6894 0 0 0 # C_R

579 1 4 0.1178 -5.52729 35.51479 10.76519 0 0 0 # H_

580 1 3 -0.123 -3.6938 33.47351 10.79058 0 0 0 # C_R

581 1 4 0.115 -3.68839 34.37501 11.00639 0 0 0 # H_

582 1 3 -0.1223 -2.5028 32.79988 10.6933 0 0 0 # C_R

583 1 4 0.1102 -1.70367 33.27118 10.71018 0 0 0 # H_

584 1 3 -0.1376 -17.86183 40.57872 11.72961 0 0 0 # C_R

585 1 4 0.1583 -17.37272 40.48861 12.5128 0 0 0 # H_

586 1 3 -0.1395 -19.0895 41.2545 11.73042 0 0 0 # C_R

587 1 4 0.1324 -19.43801 41.56081 12.534 0 0 0 # H_

588 1 3 -0.1283 -22.29299 41.62569 10.2447 0 0 0 # C_R

589 1 4 0.129 -22.29919 40.71378 10.07121 0 0 0 # H_

590 1 3 -0.1296 -23.45151 42.33121 10.1643 0 0 0 # C_R

591 1 4 0.1161 -24.22142 41.9347 9.8215 1 0 0 # H_

592 1 1 0.4538 -27.55142 28.98192 31.39898 1 0 0 # Zn3+2

593 1 1 0.439 -25.69369 31.2199 30.1689 1 0 0 # Zn3+2

594 1 1 0.4482 25.33258 13.32469 9.65228 0 0 0 # Zn3+2

595 1 2 -0.4846 -26.75648 30.43648 28.7131 1 0 0 # O_2

596 1 2 -0.4777 -28.0828 28.96908 29.49641 1 0 0 # O_2

597 1 2 -0.4782 14.01319 34.51548 11.34962 0 0 0 # O_2

598 1 2 -0.4833 14.81378 36.11488 12.69442 0 0 0 # O_2

599 1 2 -0.451 25.14018 16.7352 13.07899 0 0 0 # O_2

600 1 2 -0.4521 24.27742 17.50059 11.27929 0 0 0 # O_2

601 1 2 -0.4881 -27.10762 27.0936 31.80428 1 0 0 # O_2

602 1 2 -0.4895 -25.76832 33.0743 30.65371 1 0 0 # O_2

603 1 3 0.0043 21.39139 27.43599 19.16581 0 0 0 # C_R

604 1 3 -0.1147 20.58693 28.33569 18.47639 0 0 0 # C_R

605 1 4 0.1053 20.29381 29.1128 18.89409 0 0 0 # H_

606 1 3 0.007 20.21832 28.07299 17.16248 0 0 0 # C_R

607 1 3 -0.1135 20.65429 26.91068 16.53752 0 0 0 # C_R

608 1 4 0.1098 20.40823 26.73408 15.65692 0 0 0 # H_

609 1 3 0.0027 21.45881 26.01099 17.2268 0 0 0 # C_R

610 1 3 -0.118 21.82742 26.2736 18.5408 0 0 0 # C_R

611 1 4 0.1125 22.36428 25.6708 19.00412 0 0 0 # H_

612 1 3 0.0308 -29.3383 28.32799 23.1854 1 0 0 # C_R

613 1 3 -0.1296 -29.52771 29.36509 22.27899 1 0 0 # C_R

614 1 4 0.1208 -29.3794 30.2437 22.54169 1 0 0 # H_

615 1 3 -0.1291 22.08669 29.08631 20.98032 0 0 0 # C_R

616 1 4 0.1178 -30.06351 29.77962 20.3752 1 0 0 # H_

617 1 3 0.0297 21.86802 27.77118 20.58669 0 0 0 # C_R

618 1 3 -0.1294 22.05733 26.73498 21.49272 0 0 0 # C_R

619 1 4 0.1199 21.90909 25.8556 21.22989 0 0 0 # H_

620 1 3 -0.1288 22.46489 27.013 22.7922 0 0 0 # C_R

621 1 4 0.1224 22.59321 26.3196 23.39652 0 0 0 # H_

622 1 3 0.04 -28.05651 29.23989 27.2113 1 0 0 # C_R

623 1 3 -0.1384 -27.56201 29.94771 26.12209 1 0 0 # C_R

624 1 4 0.1534 -26.93179 30.61759 26.2528 1 0 0 # H_

625 1 3 -0.141 -28.00844 29.65032 24.83949 1 0 0 # C_R

626 1 4 0.1246 -27.6757 30.12202 24.1117 1 0 0 # H_

627 1 3 0.0284 -28.94868 28.6452 24.64649 1 0 0 # C_R

628 1 3 -0.1417 22.57882 27.93738 25.7357 0 0 0 # C_R

629 1 4 0.1259 21.94808 27.2657 25.60541 0 0 0 # H_

630 1 3 -0.1349 -28.9973 28.23482 27.0183 1 0 0 # C_R

631 1 4 0.1563 22.69197 27.76132 27.7466 0 0 0 # H_

632 1 3 0.6177 -27.42111 29.45569 28.57181 1 0 0 # C_R

633 1 3 0.0327 19.36131 28.9935 16.3911 0 0 0 # C_R

634 1 3 -0.1237 19.26999 30.2833 16.90101 0 0 0 # C_R

635 1 4 0.1101 19.70591 30.5005 17.69232 0 0 0 # H_

636 1 3 -0.1229 18.52709 31.24558 16.2273 0 0 0 # C_R

637 1 4 0.1154 18.46523 32.10878 16.56671 0 0 0 # H_

638 1 3 0.0349 17.87583 30.9185 15.0438 0 0 0 # C_R

639 1 3 -0.1241 17.9671 29.62869 14.5339 0 0 0 # C_R

640 1 4 0.1075 17.53141 29.41019 13.74001 0 0 0 # H_

641 1 3 -0.1254 18.71021 28.66588 15.20748 0 0 0 # C_R

642 1 4 0.1121 18.77209 27.80182 14.86561 0 0 0 # H_

643 1 3 0.0332 17.01556 32.05882 14.45011 0 0 0 # C_R

644 1 3 -0.1428 16.90577 33.39962 14.79922 0 0 0 # C_R

645 1 4 0.119 17.35449 33.72621 15.5469 0 0 0 # H_

646 1 3 -0.1406 16.12451 34.25562 14.03241 0 0 0 # C_R

647 1 4 0.1641 16.05141 35.15441 14.26468 0 0 0 # H_

648 1 3 0.033 15.45259 33.7713 12.91611 0 0 0 # C_R

649 1 3 -0.1304 15.56262 32.43001 12.56701 0 0 0 # C_R

650 1 4 0.15 15.11239 32.10432 11.8189 0 0 0 # H_

651 1 3 -0.1293 16.34399 31.5741 13.33381 0 0 0 # C_R

652 1 4 0.1131 16.41552 30.67621 13.10099 0 0 0 # H_

653 1 3 0.613 14.7009 34.91559 12.26948 0 0 0 # C_R

654 1 3 0.0269 21.89369 24.67469 16.56248 0 0 0 # C_R

655 1 3 -0.1276 21.0507 24.00841 15.68231 0 0 0 # C_R

656 1 4 0.1188 20.19749 24.3418 15.513 0 0 0 # H_

657 1 3 -0.1256 21.47731 22.8461 15.0518 0 0 0 # C_R

658 1 4 0.1094 20.91282 22.4 14.46361 0 0 0 # H_

659 1 3 0.0383 22.75182 22.35048 15.30151 0 0 0 # C_R

660 1 3 -0.1229 23.5946 23.0173 16.18591 0 0 0 # C_R

661 1 4 0.1104 24.44771 22.68392 16.35509 0 0 0 # H_

662 1 3 -0.1276 23.1628 24.17961 16.81642 0 0 0 # C_R

663 1 4 0.1212 23.72978 24.62112 17.40461 0 0 0 # H_

664 1 3 0.0362 23.28141 21.23859 14.60029 0 0 0 # C_R

665 1 3 -0.1296 22.42117 20.53172 13.76751 0 0 0 # C_R

666 1 4 0.1132 21.55789 20.84118 13.62152 0 0 0 # H_

667 1 3 -0.1331 22.85378 19.36171 13.15439 0 0 0 # C_R

668 1 4 0.1381 22.2784 18.89051 12.59752 0 0 0 # H_

669 1 3 0.0233 24.1458 18.89812 13.37401 0 0 0 # C_R

670 1 3 -0.1351 25.00541 19.60499 14.20679 0 0 0 # C_R

671 1 4 0.1384 25.86791 19.29589 14.35359 0 0 0 # H_

672 1 3 -0.1297 24.57309 20.77541 14.81949 0 0 0 # C_R

673 1 4 0.1137 25.14738 21.2467 15.37759 0 0 0 # H_

674 1 3 0.5777 24.59083 17.74158 12.50861 0 0 0 # C_R

675 1 3 -0.1343 24.87024 24.36338 31.84529 0 0 0 # C_R

676 1 4 0.1568 24.0654 24.8239 31.92319 0 0 0 # H_

677 1 3 -0.136 24.84651 22.98171 31.8474 0 0 0 # C_R

678 1 4 0.1178 24.02901 22.54421 31.92319 0 0 0 # H_

679 1 3 -0.123 24.87996 19.93572 31.94858 0 0 0 # C_R

680 1 4 0.115 24.09659 20.38169 32.16439 0 0 0 # H_

681 1 3 -0.1224 24.86779 18.56739 31.8513 0 0 0 # C_R

682 1 4 0.1102 24.0602 18.1111 31.86818 0 0 0 # H_

683 1 3 -0.1376 -26.21131 35.75811 32.88761 1 0 0 # C_R

684 1 4 0.1583 -26.37778 35.28948 33.6708 1 0 0 # H_

685 1 3 -0.1395 -26.18272 37.1592 32.88842 1 0 0 # C_R

686 1 4 0.1324 -26.27369 37.61418 33.692 1 0 0 # H_

687 1 3 -0.1283 -24.90239 40.11909 31.4027 1 0 0 # C_R

688 1 4 0.1291 -24.1096 39.66861 31.22921 1 0 0 # H_

689 1 3 -0.1296 -24.93409 41.47522 31.3223 1 0 0 # C_R

690 1 4 0.1161 -24.2058 41.9438 30.9795 0 0 0 # H_

691 1 1 0.4538 14.68763 6.70118 31.39898 0 0 0 # Zn3+2

692 1 1 0.439 11.82059 7.19108 30.1689 0 0 0 # Zn3+2

693 1 1 0.4482 27.81619 15.2764 9.65228 0 0 0 # Zn3+2

694 1 2 -0.4846 13.0305 6.6623 28.7131 0 0 0 # O_2

695 1 2 -0.4777 14.96439 6.24741 29.49641 0 0 0 # O_2

696 1 2 -0.4782 -10.88693 39.93041 11.34962 0 0 0 # O_2

697 1 2 -0.4833 -12.67233 39.824 12.69442 0 0 0 # O_2

698 1 2 -0.451 24.95893 13.40439 13.07899 0 0 0 # O_2

699 1 2 -0.4521 24.72741 12.27452 11.27929 0 0 0 # O_2

700 1 2 -0.4881 16.10112 8.02968 31.80428 0 0 0 # O_2

701 1 2 -0.4895 10.25198 6.19921 30.65371 0 0 0 # O_2

702 1 3 0.0043 17.56598 4.80749 19.16581 0 0 0 # C_R

703 1 3 -0.1146 17.18908 3.661 18.47639 0 0 0 # C_R

704 1 4 0.1053 16.6626 3.0185 18.89409 0 0 0 # H_

705 1 3 0.007 17.60091 3.4731 17.16248 0 0 0 # C_R

706 1 3 -0.1135 18.38952 4.4318 16.53752 0 0 0 # C_R

707 1 4 0.1098 18.6655 4.307 15.65692 0 0 0 # H_

708 1 3 0.0027 18.76642 5.57838 17.2268 0 0 0 # C_R

709 1 3 -0.118 18.35469 5.7663 18.5408 0 0 0 # C_R

710 1 4 0.1125 18.60832 6.53259 19.00412 0 0 0 # H_

711 1 3 0.0308 16.14737 5.48062 23.1854 0 0 0 # C_R

712 1 3 -0.1296 15.34389 4.79808 22.27899 0 0 0 # C_R

713 1 4 0.1208 14.50889 4.4872 22.54169 0 0 0 # H_

714 1 3 -0.1291 15.78922 4.58448 20.98032 0 0 0 # C_R

715 1 4 0.1178 15.2528 4.1268 20.3752 0 0 0 # H_

716 1 3 0.0297 17.03749 5.05258 20.58669 0 0 0 # C_R

717 1 3 -0.1293 17.84016 5.73472 21.49272 0 0 0 # C_R

718 1 4 0.1199 18.67592 6.04598 21.22989 0 0 0 # H_

719 1 3 -0.1288 17.39558 5.94872 22.7922 0 0 0 # C_R

720 1 4 0.1224 17.93201 6.4064 23.39652 0 0 0 # H_

721 1 3 0.04 14.71682 6.13478 27.2113 0 0 0 # C_R

722 1 3 -0.1384 13.85658 6.20912 26.12209 0 0 0 # C_R

723 1 4 0.1534 12.96131 6.42001 26.2528 0 0 0 # H_

724 1 3 -0.141 14.33729 5.9712 24.83949 0 0 0 # C_R

725 1 4 0.1246 13.76242 6.0235 24.1117 0 0 0 # H_

726 1 3 0.0284 15.67792 5.65948 24.64649 0 0 0 # C_R

727 1 3 -0.1417 16.53808 5.5851 25.7357 0 0 0 # C_R

728 1 4 0.1259 17.4352 5.3747 25.60541 0 0 0 # H_

729 1 3 -0.135 16.0576 5.82261 27.0183 0 0 0 # C_R

730 1 4 0.1563 16.63398 5.77121 27.7466 0 0 0 # H_

731 1 3 0.6177 14.2122 6.5772 28.57181 0 0 0 # C_R

732 1 3 0.0327 17.23231 2.2706 16.3911 0 0 0 # C_R

733 1 3 -0.1237 16.16092 1.5466 16.90101 0 0 0 # C_R

734 1 4 0.1101 15.75492 1.8156 17.69232 0 0 0 # H_

735 1 3 -0.1229 15.69891 0.4221 16.2273 0 0 0 # C_R

736 1 4 0.1154 -11.02872 44.9893 16.56671 0 0 0 # H_

737 1 3 0.0349 16.30792 0.0216 15.0438 0 0 0 # C_R

738 1 3 -0.1241 17.37921 0.7456 14.5339 0 0 0 # C_R

739 1 4 0.1075 17.7863 0.4776 13.74001 0 0 0 # H_

740 1 3 -0.1254 17.8415 1.8706 15.20748 0 0 0 # C_R

741 1 4 0.1121 18.55882 2.3562 14.86561 0 0 0 # H_

742 1 3 0.0332 -10.26061 43.75892 14.45011 0 0 0 # C_R

743 1 3 -0.1428 -11.36681 42.99348 14.79922 0 0 0 # C_R

744 1 4 0.1189 -11.874 43.2187 15.5469 0 0 0 # H_

745 1 3 -0.1405 -11.71738 41.8888 14.03241 0 0 0 # C_R

746 1 4 0.1641 -12.45932 41.3761 14.26468 0 0 0 # H_

747 1 3 0.033 -10.96208 41.5491 12.91611 0 0 0 # C_R

748 1 3 -0.1304 -9.85562 42.31499 12.56701 0 0 0 # C_R

749 1 4 0.15 -9.34838 42.08788 11.8189 0 0 0 # H_

750 1 3 -0.1293 -9.50491 43.41972 13.33381 0 0 0 # C_R

751 1 4 0.1131 -8.76313 43.93061 13.10099 0 0 0 # H_

752 1 3 0.6131 -11.57721 40.32588 12.26948 0 0 0 # C_R

753 1 3 0.0269 19.70622 6.6231 16.56248 0 0 0 # C_R

754 1 3 -0.1276 20.70478 6.22619 15.68231 0 0 0 # C_R

755 1 4 0.1188 20.84261 5.32069 15.513 0 0 0 # H_

756 1 3 -0.1256 21.49812 7.1768 15.0518 0 0 0 # C_R

757 1 4 0.1094 22.1666 6.91099 14.46361 0 0 0 # H_

758 1 3 0.0383 21.29 8.52841 15.30151 0 0 0 # C_R

759 1 3 -0.1229 20.29121 8.92492 16.18591 0 0 0 # C_R

760 1 4 0.1104 20.1533 9.83038 16.35509 0 0 0 # H_

761 1 3 -0.1276 19.50037 7.96981 16.81642 0 0 0 # C_R

762 1 4 0.1212 18.83462 8.24008 17.40461 0 0 0 # H_

763 1 3 0.0362 21.98809 9.54299 14.60029 0 0 0 # C_R

764 1 3 -0.1296 23.0304 9.15149 13.76751 0 0 0 # C_R

765 1 4 0.1132 23.19401 8.24909 13.62152 0 0 0 # H_

766 1 3 -0.1331 23.82738 10.1111 13.15439 0 0 0 # C_R

767 1 4 0.1381 24.5232 9.8484 12.59752 0 0 0 # H_

768 1 3 0.0233 23.5829 11.46182 13.37401 0 0 0 # C_R

769 1 3 -0.1351 22.5409 11.85278 14.20679 0 0 0 # C_R

770 1 4 0.1384 22.37729 12.75428 14.35359 0 0 0 # H_

771 1 3 -0.1297 21.74338 10.89321 14.81949 0 0 0 # C_R

772 1 4 0.1137 21.04808 11.15501 15.37759 0 0 0 # H_

773 1 3 0.5777 24.36188 12.4254 12.50861 0 0 0 # C_R

774 1 3 -0.1343 18.4876 9.35652 31.84529 0 0 0 # C_R

775 1 4 0.1568 18.49122 8.4293 31.92319 0 0 0 # H_

776 1 3 -0.1359 19.69602 10.0269 31.8474 0 0 0 # C_R

777 1 4 0.1178 20.48371 9.53759 31.92319 0 0 0 # H_

778 1 3 -0.123 22.31718 11.57891 31.94858 0 0 0 # C_R

779 1 4 0.115 22.32259 10.67741 32.16439 0 0 0 # H_

780 1 3 -0.1224 23.5082 12.2524 31.8513 0 0 0 # C_R

781 1 4 0.1102 24.30728 11.7812 31.86818 0 0 0 # H_

782 1 3 -0.1376 8.14919 4.4737 32.88761 0 0 0 # C_R

783 1 4 0.1583 8.63831 4.56381 33.6708 0 0 0 # H_

784 1 3 -0.1395 6.92148 3.7979 32.88842 0 0 0 # C_R

785 1 4 0.1324 6.57301 3.4916 33.692 0 0 0 # H_

786 1 3 -0.1283 3.718 3.4267 31.4027 0 0 0 # C_R

787 1 4 0.1291 3.7118 4.3385 31.22921 0 0 0 # H_

788 1 3 -0.1296 2.5595 2.7212 31.3223 0 0 0 # C_R

789 1 4 0.1161 1.7896 3.1176 30.9795 0 0 0 # H_

790 1 1 0.4538 -13.14721 9.36918 31.39898 1 0 0 # Zn3+2

791 1 1 0.439 -12.13801 6.6414 30.1689 1 0 0 # Zn3+2

792 1 1 0.4482 24.88423 16.45128 9.65228 0 0 0 # Zn3+2

793 1 2 -0.4846 -12.28502 7.9535 28.7131 1 0 0 # O_2

794 1 2 -0.4777 -12.89259 9.83579 29.49641 1 0 0 # O_2

795 1 2 -0.4782 -3.12629 15.6589 11.34962 0 0 0 # O_2

796 1 2 -0.4833 -2.14149 14.16582 12.69442 0 0 0 # O_2

797 1 2 -0.451 27.93399 14.91279 13.07899 0 0 0 # O_2

798 1 2 -0.4521 29.0283 15.2773 11.27929 0 0 0 # O_2

799 1 2 -0.4881 -15.00439 9.92909 31.80428 1 0 0 # O_2

800 1 2 -0.4895 -10.49468 5.77891 30.65371 1 0 0 # O_2

801 1 3 0.0043 -12.94648 12.8088 19.16581 1 0 0 # C_R

802 1 3 -0.1147 -11.76501 13.05568 18.47639 1 0 0 # C_R

803 1 4 0.1053 -10.94543 12.92102 18.89409 1 0 0 # H_

804 1 3 0.007 -11.80819 13.5063 17.16248 1 0 0 # C_R

805 1 3 -0.1135 -13.03281 13.70989 16.53752 1 0 0 # C_R

806 1 4 0.1098 -13.06267 14.01129 15.65692 1 0 0 # H_

807 1 3 0.0027 -14.21418 13.463 17.2268 1 0 0 # C_R

808 1 3 -0.118 -14.17113 13.01252 18.5408 1 0 0 # C_R

809 1 4 0.1125 -14.9615 12.84889 19.00412 1 0 0 # H_

810 1 3 0.0308 -12.81999 11.24372 23.1854 1 0 0 # C_R

811 1 3 -0.1296 -11.82723 10.8892 22.27899 1 0 0 # C_R

812 1 4 0.1208 -11.14051 10.3215 22.54169 1 0 0 # H_

813 1 3 -0.1291 -11.86487 11.38158 20.98032 1 0 0 # C_R

814 1 4 0.1178 -11.20031 11.146 20.3752 1 0 0 # H_

815 1 3 0.0297 -12.89441 12.22861 20.58669 1 0 0 # C_R

816 1 3 -0.1293 -13.88652 12.58272 21.49272 1 0 0 # C_R

817 1 4 0.1199 -14.57402 13.15079 21.22989 1 0 0 # H_

818 1 3 -0.1289 -13.84961 12.09071 22.7922 1 0 0 # C_R

819 1 4 0.1224 -14.51411 12.32628 23.39652 1 0 0 # H_

820 1 3 0.04 -12.67131 9.6777 27.2113 1 0 0 # C_R

821 1 3 -0.1384 -12.30549 8.89559 26.12209 1 0 0 # C_R

822 1 4 0.1534 -12.04049 8.01482 26.2528 1 0 0 # H_

823 1 3 -0.141 -12.33993 9.43081 24.83949 1 0 0 # C_R

824 1 4 0.1246 -12.09769 8.9069 24.1117 1 0 0 # H_

825 1 3 0.0284 -12.74019 10.74769 24.64649 1 0 0 # C_R

826 1 3 -0.1417 -13.1059 11.5298 25.7357 1 0 0 # C_R

827 1 4 0.1259 -13.37228 12.41188 25.60541 1 0 0 # H_

828 1 3 -0.1349 -13.07128 10.99499 27.0183 1 0 0 # C_R

829 1 4 0.1563 -13.31498 11.51989 27.7466 1 0 0 # H_

830 1 3 0.6177 -12.80209 9.01949 28.57181 1 0 0 # C_R

831 1 3 0.0327 -10.58258 13.78828 16.3911 1 0 0 # C_R

832 1 3 -0.1237 -9.41994 13.22242 16.90101 1 0 0 # C_R

833 1 4 0.1101 -9.4498 12.73631 17.69232 1 0 0 # H_

834 1 3 -0.1229 -8.21511 13.38461 16.2273 1 0 0 # C_R

835 1 4 0.1154 -7.43651 13.00662 16.56671 0 0 0 # H_

836 1 3 0.0349 -8.17271 14.11221 15.0438 1 0 0 # C_R

837 1 3 -0.1241 -9.33532 14.67811 14.5339 1 0 0 # C_R

838 1 4 0.1076 -9.30671 15.16458 13.74001 1 0 0 # H_

839 1 3 -0.1254 -10.54072 14.51592 15.20748 1 0 0 # C_R

840 1 4 0.1121 -11.31999 14.89431 14.86561 1 0 0 # H_

841 1 3 0.0332 -6.7551 14.28701 14.45011 0 0 0 # C_R

842 1 3 -0.1428 -5.539 13.71169 14.79922 0 0 0 # C_R

843 1 4 0.1189 -5.4805 13.1598 15.5469 0 0 0 # H_

844 1 3 -0.1406 -4.40699 13.96038 14.03241 0 0 0 # C_R

845 1 4 0.1641 -3.59209 13.57428 14.26468 0 0 0 # H_

846 1 3 0.033 -4.49049 14.78439 12.91611 0 0 0 # C_R

847 1 3 -0.1304 -5.7071 15.35971 12.56701 0 0 0 # C_R

848 1 4 0.15 -5.764 15.9125 11.8189 0 0 0 # H_

849 1 3 -0.1293 -6.83911 15.11102 13.33381 0 0 0 # C_R

850 1 4 0.1131 -7.65241 15.49802 13.10099 0 0 0 # H_

851 1 3 0.613 -3.12369 14.86318 12.26948 0 0 0 # C_R

852 1 3 0.0269 -15.58891 13.75449 16.56248 1 0 0 # C_R

853 1 3 -0.1276 -15.74448 14.81768 15.68231 1 0 0 # C_R

854 1 4 0.1188 -15.02921 15.38989 15.513 1 0 0 # H_

855 1 3 -0.1256 -16.9644 15.02952 15.0518 1 0 0 # C_R

856 1 4 0.1094 -17.06842 15.7413 14.46361 1 0 0 # H_

857 1 3 0.0383 -18.0308 14.17352 15.30151 1 0 0 # C_R

858 1 3 -0.1229 -17.87479 13.1102 16.18591 1 0 0 # C_R

859 1 4 0.1104 -18.59009 12.53812 16.35509 1 0 0 # H_

860 1 3 -0.1276 -16.65219 12.903 16.81642 1 0 0 # C_R

861 1 4 0.1212 -16.55343 12.19122 17.40461 1 0 0 # H_

862 1 3 0.0362 -19.25849 14.27079 14.60029 1 0 0 # C_R

863 1 3 -0.1296 -19.4406 15.36921 13.76751 1 0 0 # C_R

864 1 4 0.1132 -18.7409 15.9621 13.62152 1 0 0 # H_

865 1 3 -0.1331 -20.67019 15.57961 13.15439 1 0 0 # C_R

866 1 4 0.1381 -20.79062 16.31351 12.59752 1 0 0 # H_

867 1 3 0.0233 30.30443 14.69248 13.37401 0 0 0 # C_R

868 1 3 -0.1351 30.48669 13.5946 14.20679 0 0 0 # C_R

869 1 4 0.1384 29.7878 13.00212 14.35359 0 0 0 # H_

870 1 3 -0.1297 -20.30549 13.38371 14.81949 1 0 0 # C_R

871 1 4 0.1137 -20.18448 12.65071 15.37759 1 0 0 # H_

872 1 3 0.5777 29.0803 14.8853 12.50861 0 0 0 # C_R

873 1 3 -0.1343 -17.34671 11.33252 31.84529 1 0 0 # C_R

874 1 4 0.1568 -16.5456 11.79922 31.92319 1 0 0 # H_

875 1 3 -0.136 -18.53151 12.0439 31.8474 1 0 0 # C_R

876 1 4 0.1178 -18.50157 12.97058 31.92319 1 0 0 # H_

877 1 3 -0.123 30.83583 13.53779 31.94858 0 0 0 # C_R

878 1 4 0.115 -20.4082 13.99331 32.16439 1 0 0 # H_

879 1 3 -0.1224 29.65701 14.2325 31.8513 0 0 0 # C_R

880 1 4 0.1102 29.66552 15.16008 31.86818 0 0 0 # H_

881 1 3 -0.1376 -7.94901 4.8206 32.88761 1 0 0 # C_R

882 1 4 0.1583 -8.27152 5.199 33.6708 1 0 0 # H_

883 1 3 -0.1395 -6.74988 4.0953 32.88842 1 0 0 # C_R

884 1 4 0.1324 -6.31028 3.9466 33.692 1 0 0 # H_

885 1 3 -0.1283 -4.82658 1.5066 31.4027 1 0 0 # C_R

886 1 4 0.129 -5.61322 1.0452 31.22921 1 0 0 # H_

887 1 3 -0.1296 -3.63629 0.856 31.3223 1 0 0 # C_R

888 1 4 0.1161 -29.60569 45.04341 30.9795 1 0 0 # H_

889 1 1 0.4538 13.14721 9.36918 32.07502 0 0 0 # Zn3+2

890 1 1 0.439 12.13801 6.6414 33.3051 0 0 0 # Zn3+2

891 1 2 -0.4846 12.28502 7.9535 34.7609 0 0 0 # O_2

892 1 2 -0.4778 12.89259 9.83579 33.97759 0 0 0 # O_2

893 1 2 -0.4782 3.12629 15.6589 9.80838 0 0 0 # O_2

894 1 2 -0.4833 2.14149 14.16582 8.46358 0 0 0 # O_2

895 1 2 -0.4788 23.6466 13.75449 8.6579 0 0 0 # O_2

896 1 2 -0.4955 23.32203 15.89898 8.8779 0 0 0 # O_2

897 1 2 -0.4881 15.00439 9.92909 31.66972 0 0 0 # O_2

898 1 2 -0.4895 10.49468 5.77891 32.82029 0 0 0 # O_2

899 1 3 0.0043 12.94648 12.8088 1.9922 0 0 0 # C_R

900 1 3 -0.1147 11.76501 13.05568 2.6816 0 0 0 # C_R

901 1 4 0.1053 10.94538 12.92102 2.2639 0 0 0 # H_

902 1 3 0.007 11.80819 13.5063 3.9955 0 0 0 # C_R

903 1 3 -0.1135 13.03281 13.70989 4.62048 0 0 0 # C_R

904 1 4 0.1097 13.06272 14.01129 5.50108 0 0 0 # H_

905 1 3 0.0027 14.21418 13.463 3.9312 0 0 0 # C_R

906 1 3 -0.118 14.17108 13.01252 2.6172 0 0 0 # C_R

907 1 4 0.1125 14.9615 12.84889 2.1539 0 0 0 # H_

908 1 3 0.0308 12.81999 11.24372 40.2886 0 0 0 # C_R

909 1 3 -0.1297 11.82718 10.8892 41.19501 0 0 0 # C_R

910 1 4 0.1208 11.14051 10.3215 40.93231 0 0 0 # H_

911 1 3 -0.1291 11.86492 11.38158 0.17771 0 0 0 # C_R

912 1 4 0.1178 11.20031 11.146 0.7828 0 0 0 # H_

913 1 3 0.0297 12.89441 12.22861 0.5713 0 0 0 # C_R

914 1 3 -0.1294 13.88647 12.58272 41.98128 0 0 0 # C_R

915 1 4 0.1198 14.57402 13.15079 42.24411 0 0 0 # H_

916 1 3 -0.1289 13.84961 12.09071 40.6818 0 0 0 # C_R

917 1 4 0.1224 14.51411 12.32628 40.07748 0 0 0 # H_

918 1 3 0.04 12.67131 9.6777 36.2627 0 0 0 # C_R

919 1 3 -0.1384 12.30549 8.89559 37.35191 0 0 0 # C_R

920 1 4 0.1534 12.04049 8.01482 37.2212 0 0 0 # H_

921 1 3 -0.141 12.33988 9.43081 38.63451 0 0 0 # C_R

922 1 4 0.1247 12.09769 8.9069 39.3623 0 0 0 # H_

923 1 3 0.0284 12.74019 10.74769 38.82751 0 0 0 # C_R

924 1 3 -0.1418 13.1059 11.5298 37.7383 0 0 0 # C_R

925 1 4 0.1259 13.37233 12.41188 37.86859 0 0 0 # H_

926 1 3 -0.135 13.07128 10.99499 36.4557 0 0 0 # C_R

927 1 4 0.1562 13.31498 11.51989 35.7274 0 0 0 # H_

928 1 3 0.6177 12.80209 9.01949 34.90219 0 0 0 # C_R

929 1 3 0.0327 10.58263 13.78828 4.7669 0 0 0 # C_R

930 1 3 -0.1237 9.41988 13.22242 4.25699 0 0 0 # C_R

931 1 4 0.1101 9.4498 12.73631 3.4657 0 0 0 # H_

932 1 3 -0.1229 8.21511 13.38461 4.9307 0 0 0 # C_R

933 1 4 0.1154 7.43649 13.00662 4.59129 0 0 0 # H_

934 1 3 0.0349 8.17271 14.11221 6.1142 0 0 0 # C_R

935 1 3 -0.1241 9.33527 14.67811 6.6241 0 0 0 # C_R

936 1 4 0.1075 9.30671 15.16458 7.41799 0 0 0 # H_

937 1 3 -0.1254 10.54067 14.51592 5.95052 0 0 0 # C_R

938 1 4 0.112 11.31999 14.89431 6.29239 0 0 0 # H_

939 1 3 0.0332 6.75511 14.28701 6.70789 0 0 0 # C_R

940 1 3 -0.1428 5.53899 13.71169 6.35878 0 0 0 # C_R

941 1 4 0.1189 5.48052 13.1598 5.6111 0 0 0 # H_

942 1 3 -0.1406 4.40699 13.96038 7.12559 0 0 0 # C_R

943 1 4 0.1641 3.59212 13.57428 6.89332 0 0 0 # H_

944 1 3 0.033 4.49049 14.78439 8.24189 0 0 0 # C_R

945 1 3 -0.1304 5.70707 15.35971 8.59099 0 0 0 # C_R

946 1 4 0.15 5.76399 15.9125 9.3391 0 0 0 # H_

947 1 3 -0.1293 6.83907 15.11102 7.82419 0 0 0 # C_R

948 1 4 0.1131 7.65238 15.49802 8.05701 0 0 0 # H_

949 1 3 0.613 3.12369 14.86318 8.88852 0 0 0 # C_R

950 1 3 0.0268 15.58891 13.75449 4.59552 0 0 0 # C_R

951 1 3 -0.1278 15.74454 14.81768 5.47569 0 0 0 # C_R

952 1 4 0.1188 15.02921 15.38989 5.645 0 0 0 # H_

953 1 3 -0.1257 16.9644 15.02952 6.1062 0 0 0 # C_R

954 1 4 0.1093 17.06842 15.7413 6.69439 0 0 0 # H_

955 1 3 0.0395 18.0308 14.17352 5.85649 0 0 0 # C_R

956 1 3 -0.1228 17.87479 13.1102 4.97209 0 0 0 # C_R

957 1 4 0.1108 18.59009 12.53812 4.80291 0 0 0 # H_

958 1 3 -0.1278 16.65219 12.903 4.34158 0 0 0 # C_R

959 1 4 0.1213 16.55337 12.19122 3.7534 0 0 0 # H_

960 1 3 0.0356 19.25849 14.27079 6.55771 0 0 0 # C_R

961 1 3 -0.1346 19.4406 15.36921 7.39049 0 0 0 # C_R

962 1 4 0.1147 18.7409 15.9621 7.53648 0 0 0 # H_

963 1 3 -0.1429 20.67019 15.57961 8.00361 0 0 0 # C_R

964 1 4 0.1434 20.79062 16.31351 8.56048 0 0 0 # H_

965 1 3 0.0421 21.71762 14.69248 7.78399 0 0 0 # C_R

966 1 3 -0.1243 21.53531 13.5946 6.95121 0 0 0 # C_R

967 1 4 0.1595 22.2342 13.00212 6.80441 0 0 0 # H_

968 1 3 -0.1378 20.30549 13.38371 6.33851 0 0 0 # C_R

969 1 4 0.114 20.18448 12.65071 5.78041 0 0 0 # H_

970 1 3 0.6171 22.9417 14.8853 8.64939 0 0 0 # C_R

971 1 3 -0.1343 17.34671 11.33252 31.62871 0 0 0 # C_R

972 1 4 0.1568 16.5456 11.79922 31.55081 0 0 0 # H_

973 1 3 -0.136 18.53151 12.0439 31.6266 0 0 0 # C_R

974 1 4 0.1178 18.50162 12.97058 31.55081 0 0 0 # H_

975 1 3 -0.1229 21.18622 13.53779 31.52542 0 0 0 # C_R

976 1 4 0.115 20.4082 13.99331 31.30961 0 0 0 # H_

977 1 3 -0.1224 22.36499 14.2325 31.6227 0 0 0 # C_R

978 1 4 0.1102 22.35653 15.16008 31.60582 0 0 0 # H_

979 1 3 -0.1376 7.94901 4.8206 30.58639 0 0 0 # C_R

980 1 4 0.1583 8.27152 5.199 29.8032 0 0 0 # H_

981 1 3 -0.1395 6.74988 4.0953 30.58558 0 0 0 # C_R

982 1 4 0.1324 6.31031 3.9466 29.782 0 0 0 # H_

983 1 3 -0.1283 4.82662 1.5066 32.0713 0 0 0 # C_R

984 1 4 0.1291 5.61318 1.0452 32.24479 0 0 0 # H_

985 1 3 -0.1296 3.6363 0.856 32.1517 0 0 0 # C_R

986 1 4 0.1161 -22.41631 45.04341 32.4945 0 0 0 # H_

987 1 1 0.4538 -24.47063 28.98192 32.07502 1 0 0 # Zn3+2

988 1 1 0.439 -26.32831 31.2199 33.3051 1 0 0 # Zn3+2

989 1 2 -0.4846 -25.26547 30.43648 34.7609 1 0 0 # O_2

990 1 2 -0.4778 -23.9392 28.96908 33.97759 1 0 0 # O_2

991 1 2 -0.4782 -14.01319 34.51548 9.80838 0 0 0 # O_2

992 1 2 -0.4833 -14.81378 36.11488 8.46358 0 0 0 # O_2

993 1 2 -0.4788 26.09941 17.69662 8.6579 0 0 0 # O_2

994 1 2 -0.4955 28.11891 16.9055 8.8779 0 0 0 # O_2

995 1 2 -0.4881 -24.91438 27.0936 31.66972 1 0 0 # O_2

996 1 2 -0.4895 -26.25368 33.0743 32.82029 1 0 0 # O_2

997 1 3 0.0043 -21.39139 27.43599 1.9922 1 0 0 # C_R

998 1 3 -0.1147 -20.58687 28.33569 2.6816 1 0 0 # C_R

999 1 4 0.1053 -20.29381 29.1128 2.2639 1 0 0 # H_

1000 1 3 0.007 -20.21838 28.07299 3.9955 1 0 0 # C_R

1001 1 3 -0.1135 -20.65429 26.91068 4.62048 1 0 0 # C_R

1002 1 4 0.1097 -20.40818 26.73408 5.50108 1 0 0 # H_

1003 1 3 0.0027 -21.45881 26.01099 3.9312 1 0 0 # C_R

1004 1 3 -0.118 -21.82742 26.2736 2.6172 1 0 0 # C_R

1005 1 4 0.1125 -22.36428 25.6708 2.1539 1 0 0 # H_

1006 1 3 0.0308 -22.6837 28.32799 40.2886 1 0 0 # C_R

1007 1 3 -0.1296 -22.49429 29.36509 41.19501 1 0 0 # C_R

1008 1 4 0.1208 -22.6426 30.2437 40.93231 1 0 0 # H_

1009 1 3 -0.1291 -22.08669 29.08631 0.1777 1 0 0 # C_R

1010 1 4 0.1178 -21.95849 29.77962 0.7828 1 0 0 # H_

1011 1 3 0.0297 -21.86797 27.77118 0.5713 1 0 0 # C_R

1012 1 3 -0.1294 -22.05728 26.73498 41.98128 1 0 0 # C_R

1013 1 4 0.1198 -21.90909 25.8556 42.24411 1 0 0 # H_

1014 1 3 -0.1289 -22.46489 27.013 40.6818 1 0 0 # C_R

1015 1 4 0.1224 -22.59321 26.3196 40.07748 1 0 0 # H_

1016 1 3 0.04 -23.96549 29.23989 36.2627 1 0 0 # C_R

1017 1 3 -0.1384 -24.45999 29.94771 37.35191 1 0 0 # C_R

1018 1 4 0.1534 -25.09021 30.61759 37.2212 1 0 0 # H_

1019 1 3 -0.141 -24.01362 29.65032 38.63451 1 0 0 # C_R

1020 1 4 0.1247 -24.3463 30.12202 39.3623 1 0 0 # H_

1021 1 3 0.0284 -23.07332 28.6452 38.82751 1 0 0 # C_R

1022 1 3 -0.1418 -22.57877 27.93738 37.7383 1 0 0 # C_R

1023 1 4 0.1259 -21.94808 27.2657 37.86859 1 0 0 # H_

1024 1 3 -0.135 -23.0247 28.23482 36.4557 1 0 0 # C_R

1025 1 4 0.1562 -22.69202 27.76132 35.7274 1 0 0 # H_

1026 1 3 0.6177 -24.60089 29.45569 34.90219 1 0 0 # C_R

1027 1 3 0.0327 -19.36131 28.9935 4.7669 1 0 0 # C_R

1028 1 3 -0.1237 -19.26999 30.2833 4.25699 1 0 0 # C_R

1029 1 4 0.1101 -19.70591 30.5005 3.4657 1 0 0 # H_

1030 1 3 -0.123 -18.52709 31.24558 4.9307 1 0 0 # C_R

1031 1 4 0.1154 -18.46519 32.10878 4.59129 0 0 0 # H_

1032 1 3 0.0349 -17.87577 30.9185 6.1142 1 0 0 # C_R

1033 1 3 -0.1241 -17.9671 29.62869 6.6241 1 0 0 # C_R

1034 1 4 0.1075 -17.53141 29.41019 7.41799 1 0 0 # H_

1035 1 3 -0.1254 -18.71021 28.66588 5.95052 1 0 0 # C_R

1036 1 4 0.112 -18.77209 27.80182 6.29239 1 0 0 # H_

1037 1 3 0.0332 -17.01561 32.05882 6.70789 0 0 0 # C_R

1038 1 3 -0.1428 -16.90581 33.39962 6.35878 0 0 0 # C_R

1039 1 4 0.1189 -17.3545 33.72621 5.6111 0 0 0 # H_

1040 1 3 -0.1406 -16.12451 34.25562 7.12559 0 0 0 # C_R

1041 1 4 0.1641 -16.05141 35.15441 6.89332 0 0 0 # H_

1042 1 3 0.033 -15.4526 33.7713 8.24189 0 0 0 # C_R

1043 1 3 -0.1304 -15.5626 32.43001 8.59099 0 0 0 # C_R

1044 1 4 0.15 -15.11241 32.10432 9.3391 0 0 0 # H_

1045 1 3 -0.1293 -16.344 31.5741 7.82419 0 0 0 # C_R

1046 1 4 0.1131 -16.41551 30.67621 8.05701 0 0 0 # H_

1047 1 3 0.613 -14.7009 34.91559 8.88852 0 0 0 # C_R

1048 1 3 0.0268 -21.89369 24.67469 4.59552 1 0 0 # C_R

1049 1 3 -0.1278 -21.0507 24.00841 5.47569 1 0 0 # C_R

1050 1 4 0.1188 -20.19749 24.3418 5.645 1 0 0 # H_

1051 1 3 -0.1257 -21.47731 22.8461 6.1062 1 0 0 # C_R

1052 1 4 0.1093 -20.91282 22.4 6.69439 1 0 0 # H_

1053 1 3 0.0394 -22.75177 22.35048 5.85649 1 0 0 # C_R

1054 1 3 -0.1228 -23.5946 23.0173 4.97209 1 0 0 # C_R

1055 1 4 0.1108 -24.44771 22.68392 4.80291 1 0 0 # H_

1056 1 3 -0.1278 -23.1628 24.17961 4.34158 1 0 0 # C_R

1057 1 4 0.1213 -23.72984 24.62112 3.7534 1 0 0 # H_

1058 1 3 0.0356 -23.28141 21.23859 6.55771 1 0 0 # C_R

1059 1 3 -0.1346 -22.42122 20.53172 7.39049 1 0 0 # C_R

1060 1 4 0.1147 -21.55789 20.84118 7.53648 1 0 0 # H_

1061 1 3 -0.1429 -22.85378 19.36171 8.00361 1 0 0 # C_R

1062 1 4 0.1434 -22.2784 18.89051 8.56048 1 0 0 # H_

1063 1 3 0.0421 27.8762 18.89812 7.78399 0 0 0 # C_R

1064 1 3 -0.1243 27.01659 19.60499 6.95121 0 0 0 # C_R

1065 1 4 0.1595 26.15409 19.29589 6.80441 0 0 0 # H_

1066 1 3 -0.1378 -24.57309 20.77541 6.33851 1 0 0 # C_R

1067 1 4 0.114 -25.14738 21.2467 5.78041 1 0 0 # H_

1068 1 3 0.6171 27.43123 17.74158 8.64939 0 0 0 # C_R

1069 1 3 -0.1343 -24.87018 24.36338 31.62871 1 0 0 # C_R

1070 1 4 0.1568 -24.0654 24.8239 31.55081 1 0 0 # H_

1071 1 3 -0.136 -24.84651 22.98171 31.6266 1 0 0 # C_R

1072 1 4 0.1178 -24.02901 22.54421 31.55081 1 0 0 # H_

1073 1 3 -0.123 27.14198 19.93572 31.52542 0 0 0 # C_R

1074 1 4 0.115 -24.09659 20.38169 31.30961 1 0 0 # H_

1075 1 3 -0.1224 27.15421 18.56739 31.6227 0 0 0 # C_R

1076 1 4 0.1102 27.9618 18.1111 31.60582 0 0 0 # H_

1077 1 3 -0.1376 -25.81069 35.75811 30.58639 1 0 0 # C_R

1078 1 4 0.1583 -25.64417 35.28948 29.8032 1 0 0 # H_

1079 1 3 -0.1395 -25.83928 37.1592 30.58558 1 0 0 # C_R

1080 1 4 0.1324 -25.74831 37.61418 29.782 1 0 0 # H_

1081 1 3 -0.1283 -27.11961 40.11909 32.0713 1 0 0 # C_R

1082 1 4 0.1291 -27.9124 39.66861 32.24479 1 0 0 # H_

1083 1 3 -0.1296 -27.08791 41.47522 32.1517 1 0 0 # C_R

1084 1 4 0.1161 -27.81619 41.9438 32.4945 1 0 0 # H_

1085 1 1 0.4538 -14.68758 6.70118 32.07502 1 0 0 # Zn3+2

1086 1 1 0.439 -11.82059 7.19108 33.3051 1 0 0 # Zn3+2

1087 1 2 -0.4846 -13.0305 6.6623 34.7609 1 0 0 # O_2

1088 1 2 -0.4778 -14.96439 6.24741 33.97759 1 0 0 # O_2

1089 1 2 -0.4782 10.88688 39.93041 9.80838 0 0 0 # O_2

1090 1 2 -0.4833 12.67233 39.824 8.46358 0 0 0 # O_2

1091 1 2 -0.4788 28.28701 13.60131 8.6579 0 0 0 # O_2

1092 1 2 -0.4955 26.59211 12.24789 8.8779 0 0 0 # O_2

1093 1 2 -0.4881 -16.10107 8.02968 31.66972 1 0 0 # O_2

1094 1 2 -0.4895 -10.25203 6.19921 32.82029 1 0 0 # O_2

1095 1 3 0.0043 34.45602 4.80749 1.9922 0 0 0 # C_R

1096 1 3 -0.1147 34.83292 3.661 2.6816 0 0 0 # C_R

1097 1 4 0.1053 35.35941 3.0185 2.2639 0 0 0 # H_

1098 1 3 0.007 34.42108 3.4731 3.9955 0 0 0 # C_R

1099 1 3 -0.1135 33.63248 4.4318 4.62048 0 0 0 # C_R

1100 1 4 0.1097 33.35651 4.307 5.50108 0 0 0 # H_

1101 1 3 0.0027 33.25558 5.57838 3.9312 0 0 0 # C_R

1102 1 3 -0.118 33.66731 5.7663 2.6172 0 0 0 # C_R

1103 1 4 0.1125 33.41368 6.53259 2.1539 0 0 0 # H_

1104 1 3 0.0308 -16.14742 5.48062 40.2886 1 0 0 # C_R

1105 1 3 -0.1296 -15.34389 4.79808 41.19501 1 0 0 # C_R

1106 1 4 0.1208 -14.50887 4.4872 40.93231 1 0 0 # H_

1107 1 3 -0.1291 36.23283 4.58448 0.1777 0 0 0 # C_R

1108 1 4 0.1178 -15.25291 4.1268 0.7828 1 0 0 # H_

1109 1 3 0.0297 34.98451 5.05258 0.5713 0 0 0 # C_R

1110 1 3 -0.1294 34.18178 5.73472 41.98128 0 0 0 # C_R

1111 1 4 0.1198 33.34613 6.04598 42.24411 0 0 0 # H_

1112 1 3 -0.1289 34.62642 5.94872 40.6818 0 0 0 # C_R

1113 1 4 0.1224 34.08999 6.4064 40.07748 0 0 0 # H_

1114 1 3 0.04 -14.71676 6.13478 36.2627 1 0 0 # C_R

1115 1 3 -0.1384 -13.85663 6.20912 37.35191 1 0 0 # C_R

1116 1 4 0.1534 -12.96131 6.42001 37.2212 1 0 0 # H_

1117 1 3 -0.141 -14.33729 5.9712 38.63451 1 0 0 # C_R

1118 1 4 0.1247 -13.76242 6.0235 39.3623 1 0 0 # H_

1119 1 3 0.0284 -15.67787 5.65948 38.82751 1 0 0 # C_R

1120 1 3 -0.1418 35.48392 5.5851 37.7383 0 0 0 # C_R

1121 1 4 0.1259 34.5868 5.3747 37.86859 0 0 0 # H_

1122 1 3 -0.135 -16.0576 5.82261 36.4557 1 0 0 # C_R

1123 1 4 0.1562 35.38802 5.77121 35.7274 0 0 0 # H_

1124 1 3 0.6177 -14.2122 6.5772 34.90219 1 0 0 # C_R

1125 1 3 0.0327 34.78968 2.2706 4.7669 0 0 0 # C_R

1126 1 3 -0.1237 35.86108 1.5466 4.25699 0 0 0 # C_R

1127 1 4 0.1101 36.26709 1.8156 3.4657 0 0 0 # H_

1128 1 3 -0.123 36.32308 0.4221 4.9307 0 0 0 # C_R

1129 1 4 0.1154 11.02872 44.9893 4.59129 0 0 0 # H_

1130 1 3 0.0349 35.71411 0.0216 6.1142 0 0 0 # C_R

1131 1 3 -0.1241 34.64271 0.7456 6.6241 0 0 0 # C_R

1132 1 4 0.1075 34.2357 0.4776 7.41799 0 0 0 # H_

1133 1 3 -0.1254 34.18052 1.8706 5.95052 0 0 0 # C_R

1134 1 4 0.112 33.46317 2.3562 6.29239 0 0 0 # H_

1135 1 3 0.0333 10.26061 43.75892 6.70789 0 0 0 # C_R

1136 1 3 -0.1428 11.36681 42.99348 6.35878 0 0 0 # C_R

1137 1 4 0.1189 11.874 43.2187 5.6111 0 0 0 # H_

1138 1 3 -0.1405 11.71738 41.8888 7.12559 0 0 0 # C_R

1139 1 4 0.1641 12.45932 41.3761 6.89332 0 0 0 # H_

1140 1 3 0.033 10.96208 41.5491 8.24189 0 0 0 # C_R

1141 1 3 -0.1304 9.85562 42.31499 8.59099 0 0 0 # C_R

1142 1 4 0.15 9.34843 42.08788 9.3391 0 0 0 # H_

1143 1 3 -0.1293 9.50491 43.41972 7.82419 0 0 0 # C_R

1144 1 4 0.1131 8.76308 43.93061 8.05701 0 0 0 # H_

1145 1 3 0.613 11.57721 40.32588 8.88852 0 0 0 # C_R

1146 1 3 0.0268 32.31578 6.6231 4.59552 0 0 0 # C_R

1147 1 3 -0.1278 31.31722 6.22619 5.47569 0 0 0 # C_R

1148 1 4 0.1188 31.17939 5.32069 5.645 0 0 0 # H_

1149 1 3 -0.1257 30.52388 7.1768 6.1062 0 0 0 # C_R

1150 1 4 0.1093 29.8554 6.91099 6.69439 0 0 0 # H_

1151 1 3 0.0395 30.732 8.52841 5.85649 0 0 0 # C_R

1152 1 3 -0.1228 31.73079 8.92492 4.97209 0 0 0 # C_R

1153 1 4 0.1108 31.8687 9.83038 4.80291 0 0 0 # H_

1154 1 3 -0.1278 32.52158 7.96981 4.34158 0 0 0 # C_R

1155 1 4 0.1213 33.18743 8.24008 3.7534 0 0 0 # H_

1156 1 3 0.0356 30.03391 9.54299 6.55771 0 0 0 # C_R

1157 1 3 -0.1346 28.9916 9.15149 7.39049 0 0 0 # C_R

1158 1 4 0.1147 28.82799 8.24909 7.53648 0 0 0 # H_

1159 1 3 -0.1429 28.19462 10.1111 8.00361 0 0 0 # C_R

1160 1 4 0.1434 27.4988 9.8484 8.56048 0 0 0 # H_

1161 1 3 0.0421 28.4391 11.46182 7.78399 0 0 0 # C_R

1162 1 3 -0.1243 29.4811 11.85278 6.95121 0 0 0 # C_R

1163 1 4 0.1595 29.64471 12.75428 6.80441 0 0 0 # H_

1164 1 3 -0.1378 30.27857 10.89321 6.33851 0 0 0 # C_R

1165 1 4 0.114 30.97387 11.15501 5.78041 0 0 0 # H_

1166 1 3 0.6171 27.66012 12.4254 8.64939 0 0 0 # C_R

1167 1 3 -0.1343 33.5344 9.35652 31.62871 0 0 0 # C_R

1168 1 4 0.1567 33.53078 8.4293 31.55081 0 0 0 # H_

1169 1 3 -0.1359 32.32598 10.0269 31.6266 0 0 0 # C_R

1170 1 4 0.1178 31.53829 9.53759 31.55081 0 0 0 # H_

1171 1 3 -0.1229 29.70477 11.57891 31.52542 0 0 0 # C_R

1172 1 4 0.115 29.69941 10.67741 31.30961 0 0 0 # H_

1173 1 3 -0.1224 28.5138 12.2524 31.6227 0 0 0 # C_R

1174 1 4 0.1102 27.71472 11.7812 31.60582 0 0 0 # H_

1175 1 3 -0.1376 -8.14919 4.4737 30.58639 1 0 0 # C_R

1176 1 4 0.1583 -8.63831 4.56381 29.8032 1 0 0 # H_

1177 1 3 -0.1394 -6.92152 3.7979 30.58558 1 0 0 # C_R

1178 1 4 0.1324 -6.573 3.4916 29.782 1 0 0 # H_

1179 1 3 -0.1283 -3.71802 3.4267 32.0713 1 0 0 # C_R

1180 1 4 0.1291 -3.7118 4.3385 32.24479 1 0 0 # H_

1181 1 3 -0.1296 -2.5595 2.7212 32.1517 1 0 0 # C_R

1182 1 4 0.1161 -1.7896 3.1176 32.4945 0 0 0 # H_

1183 1 1 0.485 0 30.03489 33.43599 0 0 0 # Zn3+2

1184 1 1 0.485 26.011 15.01749 12.27799 0 0 0 # Zn3+2

1185 1 5 -0.2231 0 30.03489 31.737 0 0 0 # O_3

1186 1 5 -0.2231 26.011 15.01749 10.579 0 0 0 # O_3

1187 1 5 -0.2247 0 15.011 10.579 0 0 0 # O_3

1188 1 3 0.6237 0 18.48499 10.579 0 0 0 # C_R

1189 1 3 0.0297 0 19.97622 10.579 0 0 0 # C_R

1190 1 3 0.0287 2.6011E-5 22.82799 10.579 0 0 0 # C_R

1191 1 3 0.0287 0 24.41388 10.579 0 0 0 # C_R

1192 1 3 0.0295 0 27.17109 10.579 0 0 0 # C_R

1193 1 3 0.0082 2.6011E-5 28.67939 10.579 0 0 0 # C_R

1194 1 3 0.6191 0 11.4388 10.579 0 0 0 # C_R

1195 1 3 0.0322 2.6011E-5 9.9295 10.579 0 0 0 # C_R

1196 1 3 0.0173 0 7.1859 10.579 0 0 0 # C_R

1197 1 3 0.0205 0 5.6315 10.579 0 0 0 # C_R

1198 1 3 0.0305 0 2.9419 10.579 0 0 0 # C_R

1199 1 3 0.0048 0 1.3484 10.579 0 0 0 # C_R

1200 1 5 -0.2247 13.01107 37.54692 10.579 0 0 0 # O_3

1201 1 3 0.6237 10.00253 35.80988 10.579 0 0 0 # C_R

1202 1 3 0.0297 8.71111 35.06431 10.579 0 0 0 # C_R

1203 1 3 0.0287 6.24131 33.6384 10.579 0 0 0 # C_R

1204 1 3 0.0287 4.86798 32.84539 10.579 0 0 0 # C_R

1205 1 3 0.0295 2.48012 31.46679 10.579 0 0 0 # C_R

1206 1 3 0.0082 1.1739 30.7127 10.579 0 0 0 # C_R

1207 1 3 0.6191 16.10468 39.33302 10.579 0 0 0 # C_R

1208 1 3 0.0322 -34.61018 40.0876 10.579 1 0 0 # C_R

1209 1 3 0.0173 -32.23411 41.4594 10.579 1 0 0 # C_R

1210 1 3 0.0205 -30.88811 42.2366 10.579 1 0 0 # C_R

1211 1 3 0.0305 -28.55883 43.58141 10.579 1 0 0 # C_R

1212 1 3 0.0048 -27.17879 44.37821 10.579 1 0 0 # C_R

1213 1 5 -0.2247 -13.01112 37.54692 10.579 0 0 0 # O_3

1214 1 3 0.6237 -10.00248 35.80988 10.579 0 0 0 # C_R

1215 1 3 0.0297 -8.71111 35.06431 10.579 0 0 0 # C_R

1216 1 3 0.0287 -6.24131 33.6384 10.579 0 0 0 # C_R

1217 1 3 0.0287 -4.86798 32.84539 10.579 0 0 0 # C_R

1218 1 3 0.0295 -2.48007 31.46679 10.579 0 0 0 # C_R

1219 1 3 0.0082 -1.1739 30.7127 10.579 0 0 0 # C_R

1220 1 3 0.6191 -16.10474 39.33302 10.579 0 0 0 # C_R

1221 1 3 0.0322 -17.41182 40.0876 10.579 0 0 0 # C_R

1222 1 3 0.0173 -19.7879 41.4594 10.579 0 0 0 # C_R

1223 1 3 0.0205 -21.1339 42.2366 10.579 0 0 0 # C_R

1224 1 3 0.0305 -23.46321 43.58141 10.579 0 0 0 # C_R

1225 1 3 0.0048 -24.8432 44.37821 10.579 0 0 0 # C_R

1226 1 5 -0.2247 -26.01103 30.04142 31.737 1 0 0 # O_3

1227 1 3 0.6237 -26.011 26.56738 31.737 1 0 0 # C_R

1228 1 3 0.0297 -26.01097 25.0762 31.737 1 0 0 # C_R

1229 1 3 0.0287 26.01103 22.22429 31.737 0 0 0 # C_R

1230 1 3 0.0287 26.011 20.63849 31.737 0 0 0 # C_R

1231 1 3 0.0295 26.011 17.88129 31.737 0 0 0 # C_R

1232 1 3 0.0082 26.01103 16.37289 31.737 0 0 0 # C_R

1233 1 3 0.6191 -26.01103 33.61362 31.737 1 0 0 # C_R

1234 1 3 0.0322 -26.01097 35.12279 31.737 1 0 0 # C_R

1235 1 3 0.0173 -26.011 37.86652 31.737 1 0 0 # C_R

1236 1 3 0.0205 -26.01097 39.42078 31.737 1 0 0 # C_R

1237 1 3 0.0305 -26.01097 42.1105 31.737 1 0 0 # C_R

1238 1 3 0.0048 -26.01103 43.704 31.737 1 0 0 # C_R

1239 1 5 -0.2247 12.99991 7.5055 31.737 0 0 0 # O_3

1240 1 3 0.6237 16.00852 9.24249 31.737 0 0 0 # C_R

1241 1 3 0.0297 17.29992 9.98811 31.737 0 0 0 # C_R

1242 1 3 0.0287 19.76971 11.41402 31.737 0 0 0 # C_R

1243 1 3 0.0287 21.14302 12.2069 31.737 0 0 0 # C_R

1244 1 3 0.0295 23.53088 13.5855 31.737 0 0 0 # C_R

1245 1 3 0.0082 24.83707 14.33972 31.737 0 0 0 # C_R

1246 1 3 0.6191 9.90629 5.7194 31.737 0 0 0 # C_R

1247 1 3 0.0322 8.59921 4.96482 31.737 0 0 0 # C_R

1248 1 3 0.0173 6.2231 3.5929 31.737 0 0 0 # C_R

1249 1 3 0.0205 4.8771 2.8158 31.737 0 0 0 # C_R

1250 1 3 0.0305 2.5478 1.471 31.737 0 0 0 # C_R

1251 1 3 0.0048 1.1678 0.6742 31.737 0 0 0 # C_R

1252 1 5 -0.2247 -12.99991 7.5055 31.737 1 0 0 # O_3

1253 1 3 0.6237 -16.00852 9.24249 31.737 1 0 0 # C_R

1254 1 3 0.0297 -17.29992 9.98811 31.737 1 0 0 # C_R

1255 1 3 0.0287 32.25229 11.41402 31.737 0 0 0 # C_R

1256 1 3 0.0287 30.87898 12.2069 31.737 0 0 0 # C_R

1257 1 3 0.0295 28.49112 13.5855 31.737 0 0 0 # C_R

1258 1 3 0.0082 27.18488 14.33972 31.737 0 0 0 # C_R

1259 1 3 0.6191 -9.90629 5.7194 31.737 1 0 0 # C_R

1260 1 3 0.0322 -8.59921 4.96482 31.737 1 0 0 # C_R

1261 1 3 0.0173 -6.22312 3.5929 31.737 1 0 0 # C_R

1262 1 3 0.0205 -4.87708 2.8158 31.737 1 0 0 # C_R

1263 1 3 0.0305 -2.5478 1.471 31.737 1 0 0 # C_R

1264 1 3 0.0048 -1.16781 0.6742 31.737 1 0 0 # C_R

1265 1 3 -0.1075 1.17341 29.35748 10.579 0 0 0 # C_R

1266 1 4 0.0958 1.97939 28.89209 10.579 0 0 0 # H_

1267 1 3 -0.1074 0 31.38979 10.579 0 0 0 # C_R

1268 1 4 0.0958 -2.6011E-5 32.32062 10.579 0 0 0 # H_

1269 1 3 -0.1075 -1.17341 29.35748 10.579 0 0 0 # C_R

1270 1 4 0.0958 -1.97939 28.89209 10.579 0 0 0 # H_

1271 1 3 -0.1075 24.83759 15.6949 31.737 0 0 0 # C_R

1272 1 4 0.0958 24.03161 16.16029 31.737 0 0 0 # H_

1273 1 3 -0.1075 26.011 13.66258 31.737 0 0 0 # C_R

1274 1 4 0.0958 26.011 12.7318 31.737 0 0 0 # H_

1275 1 3 -0.1075 27.18441 15.6949 31.737 0 0 0 # C_R

1276 1 4 0.0958 27.99039 16.16029 31.737 0 0 0 # H_

1277 1 3 -0.1129 1.1767 0.6794 10.579 0 0 0 # C_R

1278 1 4 0.1045 1.982 1.1443 10.579 0 0 0 # H_

1279 1 3 -0.1128 -1.17669 0.6794 10.579 1 0 0 # C_R

1280 1 4 0.1045 -1.98202 1.1443 10.579 1 0 0 # H_

1281 1 3 -0.1128 -26.011 43.69359 10.579 1 0 0 # C_R

1282 1 4 0.1045 -26.011 42.76371 10.579 1 0 0 # H_

1283 1 3 -0.1128 -27.18769 44.37298 31.737 1 0 0 # C_R

1284 1 4 0.1045 -27.99301 43.908 31.737 1 0 0 # H_

1285 1 3 -0.1128 -24.83429 44.37298 31.737 0 0 0 # C_R

1286 1 4 0.1045 -24.029 43.908 31.737 0 0 0 # H_

1287 1 3 -0.1128 -2.601E-6 1.3588 31.737 0 0 0 # C_R

1288 1 4 0.1045 -2.601E-6 2.2887 31.737 0 0 0 # H_
